# When self-assembly meets interfacial polymerization

**DOI:** 10.1126/sciadv.adf6122

**Published:** 2023-05-03

**Authors:** Qin Shen, Qiangqiang Song, Zhaohuan Mai, Kueir-Rarn Lee, Tomohisa Yoshioka, Kecheng Guan, Ralph Rolly Gonzales, Hideto Matsuyama

**Affiliations:** ^1^ Research Center for Membrane and Film Technology, Kobe University, Kobe 657-8501, Japan.; ^2^ Department of Chemical Science and Engineering, Kobe University, Kobe 657-8501, Japan.; ^3^ R&D Center for Membrane Technology, Department of Chemical Engineering, Chung Yuan Christian University, Chung Li 32023, Taiwan.

## Abstract

Interfacial polymerization (IP) and self-assembly are two thermodynamically different processes involving an interface in their systems. When the two systems are incorporated, the interface will exhibit extraordinary characteristics and generate structural and morphological transformation. In this work, an ultrapermeable polyamide (PA) reverse osmosis (RO) membrane with crumpled surface morphology and enlarged free volume was fabricated via IP reaction with the introduction of self-assembled surfactant micellar system. The mechanisms of the formation of crumpled nanostructures were elucidated via multiscale simulations. The electrostatic interactions among *m*-phenylenediamine (MPD) molecules, surfactant monolayer and micelles, lead to disruption of the monolayer at the interface, which in turn shapes the initial pattern formation of the PA layer. The interfacial instability brought about by these molecular interactions promotes the formation of crumpled PA layer with larger effective surface area, facilitating the enhanced water transport. This work provides valuable insights into the mechanisms of the IP process and is fundamental for exploring high-performance desalination membranes.

## Teaser

Incorporating self-assembled micellar system into interfacial polymerization generates high-performance desalination membranes.

## INTRODUCTION

**Fig. 1.**
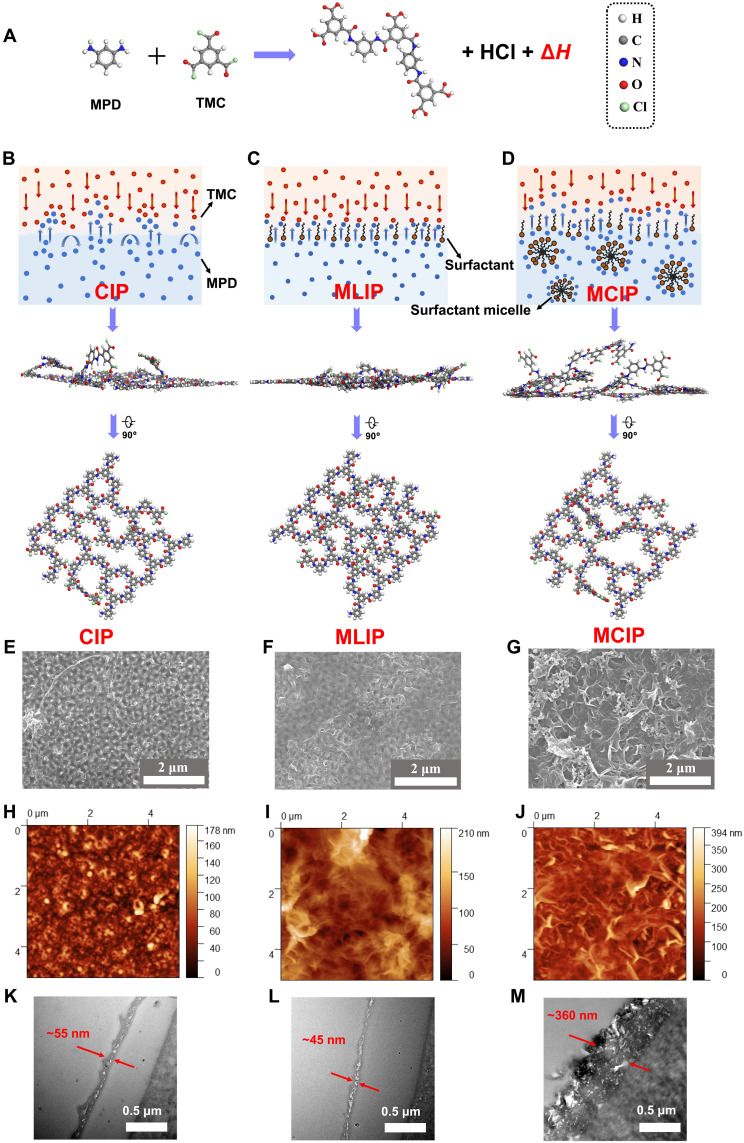
Self-assembly induced free-standing IP processes. (**A**) IP reaction and symbol representations of different atoms in this work. (**B** to **D**) Artificial schematic diagrams of three approaches (top: illustration of three systems; middle: the incipient PA films in stage I of the IP process depicted in fig. S7, time scale: ns, length scale: nm) from cross-sectional view. Bottom: Incipient PA films from top view. (B) Conventional IP process (CIP), where MPD in water and TMC in *n*-hexane react at the interface and form a thin-film PA layer. (C) Monolayer-induced IP process (MLIP), where surfactant molecules self-assemble into a stable monolayer at the interface and accelerate MPD trans-interface diffusion to form a smooth and dense PA layer. (D) MCIP, where surfactant molecules self-assemble into a monolayer (at the interface) and micelles (in aqueous solution) and the equilibrium is disrupted by the nonequilibrium diffusion-reaction IP process. The fluctuated interface that served as the platform for IP in turn shapes the initial pattern formation of the PA layer. (**E** to **G**) FESEM images of the final PA membranes in three approaches: (E) CIP, (F) MLIP, and (G) MCIP fabricated at a free-support interface and then transferred onto an AAOI (pore size, 100 nm). (**H** to **J**) AFM images of PA nanofilms in three approaches: (H) CIP, (I) MLIP, and (J) MCIP fabricated at a free-support interface and then transferred onto a silicon wafer. (**K** to **M**) Cross-sectional TEM images of PA nanofilms in three approaches: (K) CIP, (L) MLIP, and (M) MCIP fabricated at a free-support interface and then transferred onto a PAN membrane (50 kDa).

Self-assembling processes occur in every scale, from molecules to planetary systems (*1*–*3*). The emerging concepts on aggregate science have contributed to advanced material development and technological innovation (*4*–*6*). Surfactant, as an amphiphilic molecule, contains both a hydrophilic group and a hydrophobic group in its molecule, and can self-assemble into a wide variety of complex structures to minimize the interfacial tension (*7*). When the surfactant concentration is below its critical micelle concentration (CMC), a monolayer of the surfactant molecules will be formed at the interface; aside from the monolayer, micelles will also appear when the surfactant concentration reaches or surpasses its CMC (fig. S6). In a solution containing both a monolayer and micelles, the system is in thermodynamic equilibrium where surfactant molecules exchange between the monolayer and micelles equivalently and simultaneously (*7*). However, this equilibrium is very sensitive to external conditions and might be disrupted when the surfactant solution is introduced into the interfacial polymerization (IP) process, in which two reactive monomers in two immiscible phases [e.g., *m*-phenylenediamine (MPD) as the aqueous phase monomer and trimesoyl chloride (TMC) as the organic phase monomer for reverse osmosis (RO) membranes; Fig. 1, A and B] vigorously polymerize into a thin film at the interface on top of a porous support (*8*, *9*). IP is a diffusion-reaction process (fig. S7) far from thermodynamic equilibrium and has been adopted as the predominant method for fabricating polyamide (PA) layers of thin-film composite (TFC) desalination membranes to address the global challenge of water scarcity (*10*). The interactions between reactive monomers and surfactant molecules, in addition to the heat release from the exothermic IP reaction, may bring about considerable fluctuations in the self-assembly system containing both monolayer and micelles, especially at the interface. The destabilization of the interface, which serves as the reaction platform for the IP process, will reversely induce nanoscale or even subnanoscale (angstrom-scale) morphological and structural transformation of the PA layer (Fig. 1D), resulting in changes in mesoscopic or even macroscopic properties and performances of the desalination membranes based on the structure-property-performance relationship. In this case, the equilibrium self-assembly system and the nonequilibrium IP process are mutually interdependent and restrictive. Consequently, the thermodynamic state of the interface acting as the reaction zone for the IP process is of great importance.

The strategy of combining two thermodynamically totally different systems, e.g., self-assembly and polymerization, has been applied to tailor interfacial self-assembly of Janus nanoparticles in the polymerization of block copolymers to control the spatial distribution at nanoscale and manipulate macroscopic performance of the materials (*11*, *12*). This strategy is definitely inspiring for the IP process, as the manipulation of the polymer structure at the molecular level is one of the most viable strategies to overcome the intrinsic permeability-selectivity trade-off of the PA-based TFC desalination membranes, which remains one of the biggest challenges in membrane fabrication and applications (*13*–*16*).

Different from other strategies, such as reducing the thickness of the PA layer (*17*), increasing the effective surface area (*18*) or porosity (*19*), and incorporating two-dimensional materials to provide internal nanochannels for fast water transport or ion selectivity (*20*, *21*), surfactants have been extensively used in IP processes for the fabrication of high-performance desalination membranes, and most of these studies have emphasized the important role of surfactants on the support layer wettability (*22*, *23*). A recent work used surfactants as emulsifier to form nanovehicles for carrying amine monomers to the *n*-hexane phase during the IP process (*24*). However, the critical role of the thermodynamic state of the water-organic phase interface induced by surfactant self-assembly has thus far not been investigated.

A recent study has achieved sub-1-Å precision separation of ions and small molecules with the addition of surfactant interfacial network to the piperazine (PIP)–PA layer for nanofiltration (*25*), and a similar work has reported ultrahigh ion selectivity (*26*). However, they attributed the enhanced performance to the control of PIP diffusion by the surfactant network without mentioning the impact on the interface. Moreover, only the interfacial network (monolayer) has been analyzed because surfactant solutions under their CMCs were used (*25*, *26*). The exact mechanism of the micelle-assisted IP process remains to be elucidated. Besides, multiple mechanisms may affect the IP reaction in surfactant-assisted IP (*27*, *28*), and the origin of membrane surface roughness as well as its impact on desalination performance are still poorly understood. As the PA layer exhibited subnanoscale internal free volumes and nanoscale thickness, and the IP process involves multidisciplinary knowledge, the heterogeneous nature of desalination membranes (*29*) calls for multiscale investigation on the fundamental aspect to gain a thorough understanding of the IP process.

Inspired by their interfacial activities, herein, we first presumed that the addition of surfactants at concentrations higher than their CMCs to the IP process [designated as micelle-modulated IP (MCIP) in Fig. 1D] would generate PA layers that are quite different from those fabricated via monolayer-assisted IP (MLIP) with the addition of surfactants at concentrations slightly lower than their CMCs (Fig. 1C). To validate this hypothesis, we compared the morphologies of PA nanofilms at the support-free interface fabricated via conventional IP (without surfactant addition, designated as CIP; Fig. 1B), MLIP, and MCIP with an anionic surfactant, sodium dodecyl sulfate (SDS) (figs. S8 and S9). The fundamental mechanisms on the formation of different PA nanofilm morphologies with various surfactant concentrations involving interfacial monolayer and liquid phase micelles were demonstrated via multiscale simulations [i.e., molecular dynamics (MD) and dissipative particle dynamics (DPD) simulations]. Then, PA nanofilms were created by CIP, MLIP, and MCIP methods on the support membrane, and their physical-chemical properties as well as desalination performances were investigated. An ultrapermeable PA TFC RO membrane was obtained with crumpled surface and enlarged internal cavity from MCIP. This work provides an alternative perspective regarding the impact of interfacial instability induced by the combination of two thermodynamically different systems on the IP process.

## RESULTS

### Self-assembly induced freestanding IP processes

The addition of surfactants in previous studies has been considered to improve the wettability of the support and facilitate uniform MPD diffusion and promote IP reaction for denser and narrower pore structures of PA TFC membranes (*22*, *23*). To rule out the effect from the support, freestanding PA nanofilms were first fabricated at a support-free water/*n*-hexane interface in three approaches (CIP, MLIP, and MCIP, respectively) to identify the impact of surfactant self-assembly on the IP processes. The PA films were prepared from the IP reaction between MPD and TMC monomers, with different amount of SDS in the aqueous solution for CIP, MLIP, and MCIP (described in section S1.4). The CMC of SDS in this work was measured at the turning point of the interfacial tension-concentration curve as 0.01 weight % (wt %) (figs. S8 and S9). We chose 0.01 wt % SDS solution for MLIP because there was no micelle in this case, and 0.4 wt % SDS solution with an average micelle size of 1.5 nm for MCIP (fig. S9).

The surface morphological structures of freestanding PA nanofilms were examined using field-emission scanning electron microscopy (FESEM) and atomic force microscopy (AFM). The CIP PA nanofilm (Fig. 1, E and H, magnified in fig. S10), covering the anodic aluminum oxide isotropic (AAOI) support, suggests a rather thin and transparent network structure with several dispersed granular nodulations. Compared with CIP PA nanofilm, the MLIP PA nanofilm exhibits relatively smooth morphology, indicating that the MLIP PA layer is more homogeneous (Fig. 1, F and I, magnified in fig. S10). Impressively, the freestanding MCIP PA nanofilm has extensive irregular and crumpled architectures with substantially increased surface roughness (Fig. 1, G and J, magnified in fig. S10) and the AAOI support was completely unobservable. In addition, transmission electron microscope (TEM) characterizations (Fig. 1, K to M) were conducted to investigate the cross-sectional morphologies of the freestanding PA nanofilms in the three approaches, which were transferred onto a polyacrylonitrile (PAN) ultrafiltration membrane. As shown in Fig. 1K, the CIP-based PA nanofilms exhibited a thickness of ~55 nm, while the MLIP-based PA nanofilms showed a slightly decreased thickness of ~45 nm (Fig. 1L). In contrast, the MCIP PA nanofilm exhibits multilayered characteristics with larger internal voids (Fig. 1M). This is possibly explained by extensive irregular and crumpled architectures with substantially intrinsic interconnected nanocavity structure, which will be discussed in detail later. Similar results were observed in the AFM characterizations (fig. S11). Such crumpled structures are considered to increase the effective surface area and the internal free volume of the PA layer and are beneficial to enhanced water permeance (*18*).

The distinct morphological differences in the self-assembly incorporated PA nanofilms highlight the vital role of SDS self-assembly. The IP process is a diffusion-reaction process far from equilibrium, and the reaction stage is supposed to proceed in the organic phase (*25*). The IP reaction zone of a pair of MPD and TMC molecules could be roughly defined at the molecular level as the vicinity of the two reactive molecules after the MPD molecule transports from the water phase into the *n*-hexane phase and enters the contact range of any TMC molecule that is possible to conduct the subsequent IP reaction. For CIP cases, the vicinities of all MPD molecules that can transfer into the *n*-hexane phase and form a PA fragment with TMC molecule(s) constitute the whole reaction zone, which is dependent on the diffusivity, solubility in organic phase, and reactivity of the MPD molecules and the properties of the interface (*8*, *9*). In the presence of the SDS monolayer at the interface, the reaction zone is located slightly above the end of SDS alkane chain in the *n*-hexane phase (*25*). Thus, the position of the SDS monolayer as well as the depth of MPD molecules into the *n*-hexane phase are crucial to define the IP reaction zone and the final structure of the PA layer.

To illustrate the underlying mechanisms of the formation of freestanding PA nanofilm structures modulated by the self-assembly systems, we decoupled the diffusion and reaction stages of the IP process by investigating the diffusion of MPD in interfacial systems without TMC molecules via MD simulations (section S2.1) (*30*–*38*) and the reaction stage via DPD simulations (section S2.2), respectively.

**Fig. 2.**
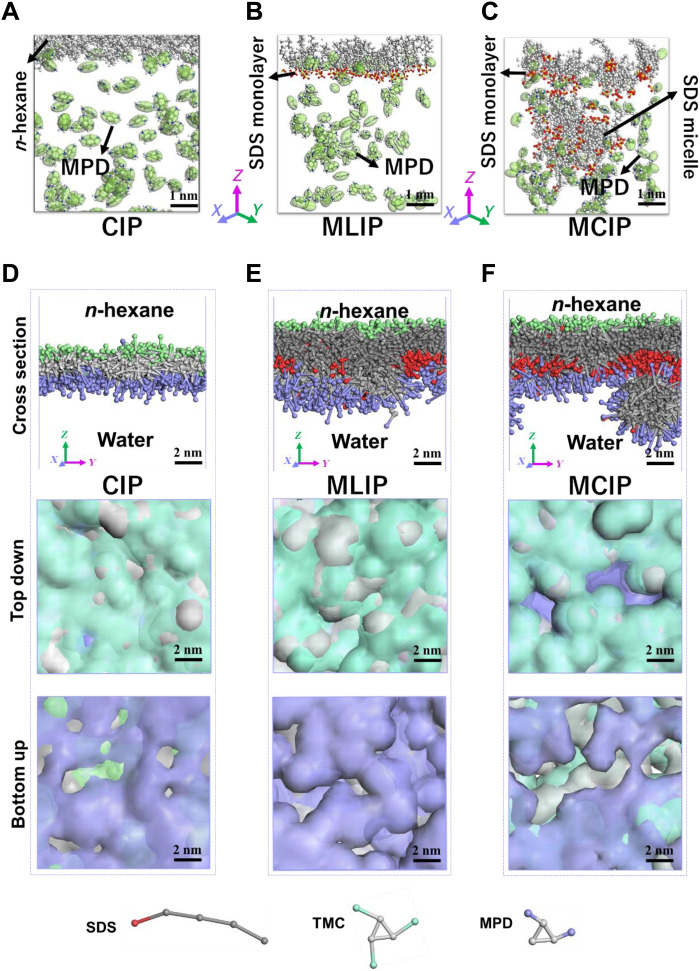
MD and DPD simulations on the morphology formation in CIP, MLIP, and MCIP. (**A** to **C**) MD snapshots of the interfaces after 1 ns (without TMC) in molecular systems during MPD diffusion in free-standing CIP (A), MLIP (B), and MCIP (C) systems. White, gray, blue, red, and yellow balls represent H, C, N, O, and S atoms, respectively. The excessive water, *n*-hexane molecules, and Na^+^ ions are set as invisible for clarity (specifically, the *n*-hexane near the interface is kept in the CIP system to identify the water/*n*-hexane interface). The green ellipsoids represent MPD molecules. Areal density of the SDS monolayer: 1.1 molecules nm^−2^, SDS micelle size: 1.5 nm. (**D** to **F**) DPD snapshots of the PA layers at *t* = 10 ns in CIP (D), MLIP (E), and MCIP (F) systems. Top, cross-sectional views of the PA layers; middle, perspective views of the top surfaces of the PA layers; bottom, perspective views of the rear surfaces. The disconnected voids with no bead coverage could be regarded as the free volume of the PA layer. Cyan, light gray, lavender, red, and dark gray beads represent -COCl group, benzene groups in MPD and TMC, -NH_2_ group, and -SO_4_^−^ and hydrocarbon group in the IP process. The excessive water and *n*-hexane beads are set as invisible for clarity.

**Fig. 3.**
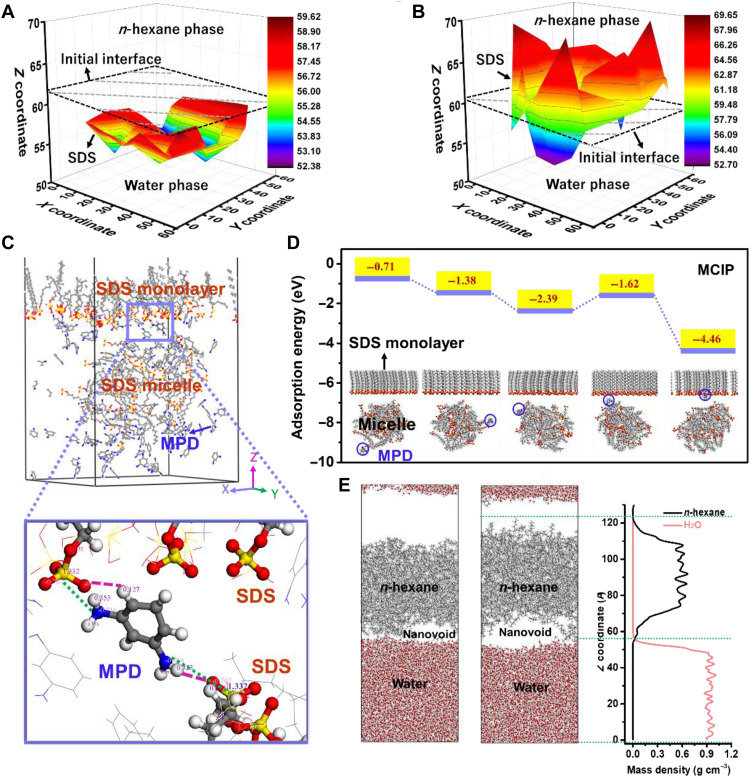
In-depth MD analysis on the thermodynamic properties of the interfaces during MPD diffusion in freestanding CIP, MLIP, and MCIP. (**A** and **B**) Three-dimensional map of S atoms in the SDS monolayer at equilibrium (color code represents *z* coordinate of S atoms): (A) in the MLIP system and (B) in the MCIP system, respectively. (**C**) Example of a molecular model of MPD in the MCIP system (top) with magnified view (bottom). Interactions between MPD and SDS molecules (both in the monolayer and in the micelle) are marked with dashed purple (hydrogen bonding between O atom in SDS and H atom in MPD) and dotted green lines (electrostatic attraction between S atom in SDS and N atom in MPD). (**D**) Adsorption energy of an MPD molecule at different positions in the MCIP system. Areal density of the SDS monolayer: 1.1 molecules nm^−2^, SDS micelle size: 1.5 nm. (**E**) Heating on the nanovoid formation at the water/*n*-hexane interface at equilibrium. Left, at 298 K; middle, at 318 K; right, mass density distributions of water and *n*-hexane in the heated system (318 K).

The interfacial stability during the MPD diffusion of the IP process was checked via MD simulations. Figure 2 (A to C) shows the MD snapshots of different diffusion systems after 1 ns (figs. S12 and S13). At the initial state, the SDS monolayers in MLIP and MCIP were organized in a well-defined manner, with sulfate head groups facing toward the water phase and the carbon chain groups directed to the *n*-hexane phase. After 1 ns, the water/*n*-hexane interface and SDS monolayer are smooth and stable in freestanding CIP and MLIP systems, indicating a relatively homogeneous interface for IP reaction in both systems (Fig. 2, A and B, and fig. S13). In contrast, the interface of the MCIP system is quite disordered, with the destabilization of both the monolayer and the micelle (Fig. 2C and fig. S13). It can be predicted that the disruption of the monolayer at the interface in the MCIP system will result in a rougher and looser incipient PA network at the initial stage of the IP process, and after the long diffusion-limited stage II, much rougher, looser, and thicker final PA membrane will be formed at the final stage (fig. S7). These morphological characteristics are in line with experimental observations (Fig. 1, E to G). Intuitively, the fluctuations of the interface that occurred in the MCIP system are primarily due to the presence of micelle, as compared with the MLIP system. The in-depth analysis of the driving force of the interfacial fluctuations will be provided later (Fig. 3).

In addition, the presence of micelles also results in larger free volumes in the MCIP system. Results from DPD simulations (*39*–*42*) of the freestanding PA nanofilms (Fig. 2, D to F) revealed that the addition of the SDS monolayer in MLIP systems facilitates the formation of a PA layer with narrower free volumes and more uniform free-volume size distribution (Fig. 2E), which has been validated previously (*25*). However, the micelles in MCIP can compensate this effect because some MPD molecules adsorb onto the SDS micelles (Fig. 2F). Therefore, less abundance of MPD molecules is expected at the interface to react with TMC molecules in the MCIP system than in the MLIP system and larger free volume is observed in the MCIP system according to the DPD simulations, as will be confirmed experimentally later.

The origin of the surface morphologies and internal free volumes was further analyzed by in-depth MD simulations. To outline the IP reaction zone, we take a closer look at the SDS monolayers in MLIP and MCIP systems. Because the hydrophilic-lipophilic balance value of SDS is 40, SDS molecules must overcome an extremely high energy penalty to enter the *n*-hexane phase (*26*). During the destabilization of the MCIP interface (Fig. 2C), the sulfate groups of SDS molecules with higher free energy can penetrate more deeply into the *n*-hexane phase and broaden the IP reaction zone. We used the depth into the *n*-hexane phase of the head group of each SDS molecule as an indirect indicator of its free energy and compared the thermodynamic states of the SDS monolayer after equilibrium in MLIP and MCIP systems (Fig. 3, A and B, and fig. S14). The positions of S atoms confirm that the SDS monolayer in MCIP experienced substantial reshuffling during the simulation process (fig. S15). These high energy state molecules could drag MPD molecules further into the *n*-hexane phase for the subsequent IP reaction and shape the initial PA fragment formation (fig. S7). The crumpled incipient PA network in the initial IP stage facilitates more heterogeneous diffusion and distribution of MPD molecules into the *n*-hexane phase in stage II of fig. S7. The characteristic for this process is the pronounced formation of crumpled PA layers with obvious inhomogeneity as observed in Fig. 1G.

The driving force of the destabilization in the SDS monolayer is further identified by analyzing the molecular interactions in the MCIP system. The interactions between MPD and SDS molecules include electrostatic attraction and hydrogen bonding (Fig. 3C and fig. S16). According to the extended Derjaguin-Landau-Verwey-Overbeek (XDLVO) theory, the electrostatic interaction energy (∆*E_EL_*, J) can be calculated by the following equation (*43*)

$$\text{Δ}E_{EL}\;(d)=\varepsilon \pi r\;\{{\left(\varphi_{1}+\varphi_{2}\right)}^{2}\;\ln\;[1+\exp\;(-\kappa d)]+{\left(\varphi_{1}-\varphi_{2}\right)}^{2}\;\ln\;[1-\exp\;(-\kappa d)]\}$$ (1)

where *r* (nm) is the radius of amine monomer, *d* (nm) is the distance between the two interacting bodies, κ is the Debye-Huckel parameter (κ^−1^ = 1.1 nm), ε is the electrical permittivity of the solution, and φ (mV) is the zeta potential.

However, there is no rule-based method for the calculation of hydrogen bonds. Because of the complexity of the self-assembly system in MLIP and MCIP, the adsorption energy (including both electrostatic interactions and hydrogen bonding) was calculated by using a simplified model (section S2.1.2 and fig. S4), assuming that the SDS monolayer and the micelle would maintain their structures as a fixed substrate. The relationship between the position of the MPD molecule relative to the monolayer and the adsorption energy is obtained in fig. S17 for the MLIP system and in Fig. 3D for the MCIP system, respectively. The negative adsorption energy indicates that the diffusive transport of an MPD molecule in the aqueous phase toward the monolayer of the MLIP/MCIP system is energetically favorable. The energy penalty for an MPD molecule to enter the *n*-hexane phase from the surfactant-free water/*n*-hexane interface (i.e., CIP system) is reported to be ~0.24 eV at 25°C (*44*). The presence of an SDS monolayer substantially transforms this repulsion (energy penalty > 0) into adsorption (energy penalty < 0) and facilitates the accumulation of MPD molecules at the water/*n*-hexane interface. For the MLIP system (fig. S17), faster and uniform transport of MPD molecules with a reduced energy barrier is responsible for the formation of a PA membrane with a smooth surface and uniform pore size distribution (*25*). In the MCIP system, the adsorption strength experiences a decrease (from 2.39 to 1.62 eV in Fig. 3D) when the MPD molecule approaches a position between the monolayer and the micelle, probably because the repulsion between the SDS monolayer and micelle compensates part of the electrostatic attraction. In the real MCIP system, the attraction from MPD molecules might lead to directional movement of the micelle as a dynamic entity toward the SDS monolayer, which shows strong repulsion to the micelle. When the repulsion from SDS micelles outweighs others (e.g., attraction between the MPD and SDS monolayer and attraction between MPD and SDS micelles), the SDS monolayer would reorganize its structure in response. Reversely, the micelle also tends to experience disintegration when exposed to this environment. Therefore, the dynamic interplay of electrostatic interactions among SDS monolayer, micelles, and MPD molecules dominates the migration of SDS molecules at the interface, thereby determining the thermodynamic stability of the IP reaction zone.

On the other hand, the IP reaction is exothermic and vigorous (Fig. 1A). The heat release from the IP reaction of MPD and TMC is reported to be 7.0 × 10^−7^ kcal mol^−1^ ps^−1^ with an interfacial area of 16.77 nm^2^, estimated from the bonding energy between MPD and TMC (*44*). To mimic the heating effect on the interface, we simplified this process by excluding MPD and TMC from the simulation systems and increased the temperature of the water-SDS/*n*-hexane system from 298 to 318 K. Different from the CIP system, where nanovoids and nanointerfaces of the water/*n*-hexane interface contribute to the roughness of the PA layer and are obviously responsive to heating (Fig. 3E and fig. S18), the monolayers in both MLIP and MCIP systems are relatively stable (figs. S19 to S21). Therefore, the driving force of the fluctuations in the interface is primarily attributed to the electrostatic interactions among different species (i.e., MPD, monolayer, and micelle) in the MCIP system.

Despite the stability of the interface, the IP reaction zone is also determined by the availability and distribution of MPD molecules after their diffusion into the *n*-hexane phase. The effect of the self-assembly system on MPD diffusion is examined by comparing diffusional characteristics in the three different MD systems. The diffusion coefficients of MPD were estimated from mean square displacement (MSD) curves in these MD systems (fig. S22 and table S1). This value in the CIP system agrees well with the experimentally determined diffusion coefficient of MPD (*45*), further validating the reliability of the MD simulations. In addition, the amount of MPD molecules diffused into the water/*n*-hexane interface in different systems can be observed directly as in the order of MLIP > MCIP > CIP (fig. S23), qualitatively consistent with experimental measurements (fig. S24). The promoted diffusion of MPD in MLIP and MCIP, compared with that in the CIP system, is ascribed to the presence of the SDS monolayer, which facilitates the accumulation of MPD monomers near the water/*n*-hexane interface via electrostatic attraction between the hydrophilic group of SDS and the amino group of MPD, similar to a previous work with PIP-PA membrane (*25*). Specifically, the attraction and physical hindrance from the SDS micelles to MPD molecules may offset the promoted diffusion toward the SDS monolayer to some extent, leading to less MPD molecules into the MCIP interface for subsequent IP reaction, as compared with the MLIP system.

**Fig. 4.**
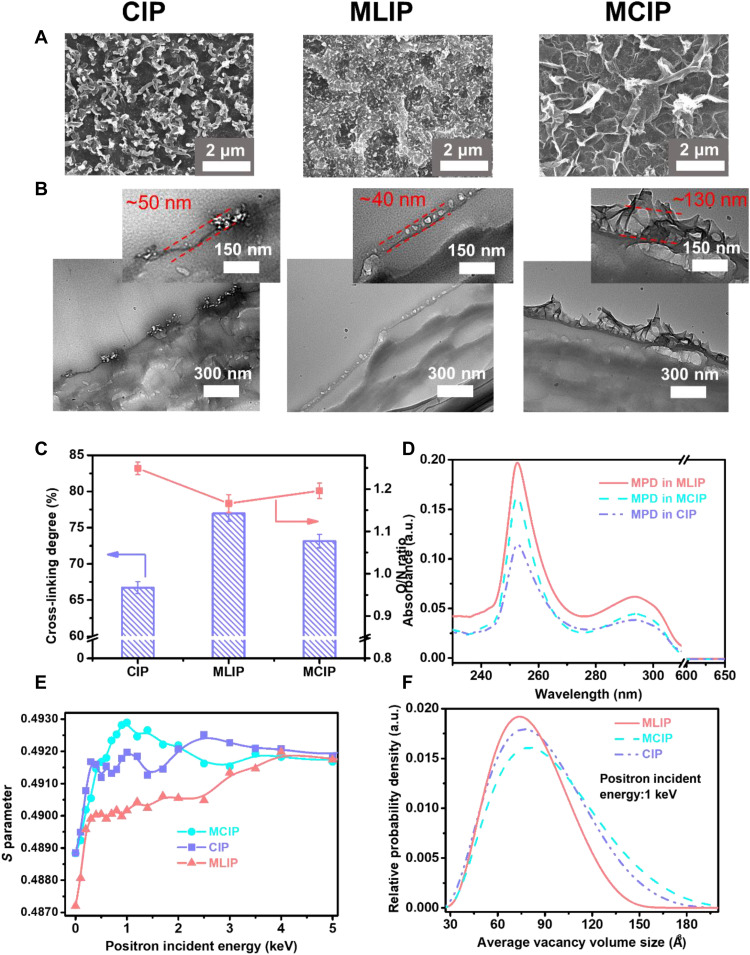
Morphological and structural properties of the PA TFC RO membranes in CIP, MLIP, and MCIP. (**A**) FESEM images of membrane surfaces. (**B**) TEM images of cross-sectional morphologies. (**C**) Cross-linking degrees and O/N ratios. (**D**) Ultraviolet-absorption spectra of MPD (maximum absorption peak: ~252.1 nm) in *n*-hexane for the measurement of MPD diffusion rate from the aqueous phase to the organic phase. (**E**) Evolutions of *S* parameters. (**F**) Free-volume size distributions calculated from PALS results.

Combined with the observations in both the position of SDS monolayer and the diffusion of MPD molecules in the three MD systems, the surface morphologies and densities of PA layers of these systems could be predicted. In the CIP case, the quantity of MPD molecules that diffused into the *n*-hexane phase is the smallest and the IP reaction zone is relatively narrow along the direction perpendicular to the water/*n*-hexane interface. The PA layer formed in CIP thereafter might be relatively thin and dense. The PA layer in the MLIP system might be the smoothest and densest as the reaction zone is the narrowest and the quantity of MPD molecules available to react with TMC molecules is the largest. This agrees well with the surface and cross-sectional morphologies characterized by AFM and TEM in Fig. 1 (I and L). As for the MCIP system, the PA layer formed thereafter might be the most crumpled because the reaction zone is the broadest and most heterogeneous in depth, yet the density of it might be the lowest because the quantity of MPD molecules that have diffused into the *n*-hexane phase is relatively small. Although the simulation time is far less than needed for the real IP processes, the present simulation results could provide valuable information on the initial formation of the PA nanofilms (fig. S7), which is a decisive factor of the final morphologies of the PA layers. The predictions on the surface morphologies and densities coincide with the AFM and TEM images of the MCIP PA network (Fig. 1, J and M) and DPD simulations (Fig. 2, D to F), respectively. The predicted densities could also be compared with the free volumes that will be provided later (Fig. 4F).

### Self-assembly induced supported IP processes

The PA TFC RO membranes via CIP, MLIP, and MCIP were fabricated with a polyketone (PK; molecular weight: 200 kg mol^−1^) support due to its high hydrophilicity compared with other commercial substrates (*46*). The PK substrate exhibits a narrow pore size distribution with pore sizes around 100 nm (fig. S25). The morphological properties of the PA TFC membranes prepared with the PK support in CIP, MLIP, and MCIP were analyzed using FESEM, TEM, and AFM (Fig. 4, A and B, and fig. S26). Compared with the freestanding PA nanofilms that yielded different textures in Fig. 1, the PA layers formed on the PK substrates in the three approaches exhibit more crumpled nanostructures (Fig. 4A and fig. S26A) with higher surface roughness (fig. S26B), while the fibrous structure of PK could still be clearly observed beneath the PA layer in the membrane prepared in MLIP. Although the cross-sectional TEM characterizations imply no obvious distinction in the intrinsic thickness of the PA films (Fig. 4B), the apparent thicknesses of the PA layers (i.e., the thickness of the crumpled PA structure) of the PA layers in these TFC membranes follow the order of MLIP < CIP < MCIP (Fig. 4B), possibly caused by the presence of internal nanovoids covered by surface crumpled nanostructures. The nanovoids emerged and became growingly prominent following the same order as MLIP < CIP < MCIP (Fig. 4B). These observations confirm our predictions in MD and DPD simulations (Figs. 2 and 3).

As explained in previous studies, the promotion of the wetting ability of the support layer via surfactant addition is favorable for creating crumpled PA surface morphologies (*25*). SDS solutions in this work rendered the PK membrane more hydrophilic as confirmed by a decrease in water contact angle (fig. S27). However, it is hard to quantify to which extent the wetting of PK by surfactant has contributed to the crumpled morphologies, because PK substrates have relatively notable intrinsic larger-scale morphology and roughness that may eclipse the smaller-scale morphology of the PA layers. Moreover, the PK substrates wetted by SDS solutions presented similar contact angles (~28.7° as shown in fig. S27) with increasing SDS concentration above the CMC. The PA layers formed on top of these PK membranes with similar wettability manifested different contact angles in MLIP (~87.6°) and MCIP (~78.2°), respectively (fig. S28), possibly due to different surface roughness resulting from the crumpled morphologies. It indirectly highlights the critical role of surfactant self-assembly on the formation of crumpled structures in these TFC RO membranes.

It can be observed from the Fourier transform infrared spectroscopy (FTIR) and x-ray photoelectron spectroscopy (XPS) (fig. S29) that amide bonds were successfully formed and the chemical composition was the same in the PA layers of the three approaches. In addition, the cross-linking degree calculated from oxygen/nitrogen (O/N) ratio probed by XPS (Fig. 4C and fig. S30) shows that the MLIP PA layer has higher cross-linking degree (77%) as compared with its counterparts (67% for CIP and 73% for MCIP, respectively). The cross-linking degrees of the PA layers agree well with the experimental measurement of MPD diffusion from the aqueous phase to the organic phase (Fig. 4D and fig. S24) and simulation results as discussed in Fig. 3 and figs. S22 and S23.

The free volumes of PA TFC RO membranes in the three approaches were measured by both positron annihilation lifetime spectroscopy (PALS) of the PA layers (Fig. 4, E and F; fig. S31; and table S2) and the rejection of neutral solutes with different molecular weights (fig. S32). The distribution of the *S* parameter (Fig. 4E) and the average vacancy volume sizes of the PA-TFC membranes (Fig. 4F) in the three approaches verify that MLIP yields the smallest free-volume cavities with the most uniform size distribution and MCIP is characterized with the largest free-volume cavities with the broadest size distribution, respectively. This free-volume tendency agrees well with the results from MD and DPD simulations (Figs. 2 and 3). Notably, the enlarged internal cavity in MCIP was not caused by the embedding of micelles, evidenced by the differences in the sizes of internal cavity (~0.27 nm; table S2) and SDS micelles (~1.5 nm; fig. S9). Full-scan XPS results of the sulfur content in the front and rear surfaces of the exfoliated PA active layers obtained from the three approaches (fig. S8, E and F) further validated that no SDS molecules were integrated into the PA active layers.

It is worth noting that a higher cross-linking degree generally indicates a denser PA layer (*17*, *25*). This is validated in MLIP PA membranes, which have the highest cross-linking degree and the narrowest free-volume distribution (Fig. 4, C and F). However, the MCIP PA membrane with a higher cross-linking degree shows a broader free-volume distribution than CIP PA membrane (Fig. 4. C and F). The inconsistency between cross-linking degree and free-volume size in MCIP and CIP systems could be explained by two reasons. First, XPS is commonly used to analyze the elemental composition of the top 5 to 10 nm of the PA surface and it is not expected to give detailed information on the internal structure of the PA layer (*47*). In addition, the crumpled structures in MCIP membrane sufficiently increase the average internal free volume of the PA layer. Therefore, a more heterogeneous structure along the thickness of the PA layer is expected in MCIP membrane.

### Desalination performances of TFC RO membranes from self-assembly induced IP

**Fig. 5.**
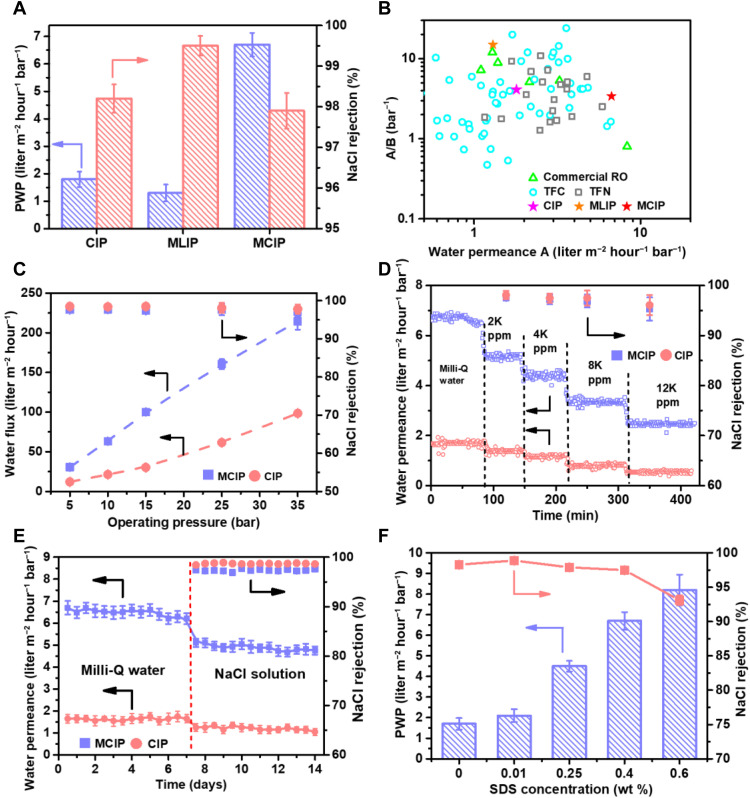
Desalination performances of the PA TFC membranes in CIP, MLIP, and MCIP. (**A**) PWP (liters m^−2^ hour^−1^ bar^−1^) and NaCl rejection with a 2000-ppm NaCl solution with an operating pressure of 15 bar. (**B**) Water/NaCl selectivity A/B (bar^−1^) and water permeance (liters m^−2^ hour^−1^ bar^−1^) of the PA RO membranes as compared with literature data (table S3). (**C**) Separation properties of PA-TFC membranes in CIP and MCIP under different operating pressures with a 2000-ppm NaCl solution. (**D**) Separation properties of PA-TFC membranes in CIP and MCIP with different concentrations of NaCl feed solutions under 15 bar. (**E**) Long-term operation of PA-TFC membranes fabricated from CIP and MCIP with a 2000-ppm NaCl solution under 15 bar. (**F**) Separation performances of the PA TFC membranes with different SDS concentrations. All PA-TFC membranes were fabricated with 0.25 wt % MPD and 0.15 wt % TMC. All tests were conducted using a cross-flow filtration cell and a cross-flow velocity of 0.6 liter hour^−1^ (25°C).

The performances of PA-TFC RO membranes from the three approaches are compared in Fig. 5A. As a control, the PA-TFC membrane formed via CIP had a relatively low water permeance of 1.8 liters m^−2^ hour^−1^ bar^−1^, accompanied with a NaCl rejection ratio of 98.2% (IP condition effect in fig. S33). The PA-TFC membrane of MLIP showed an improved NaCl rejection ratio of 99.5% and a decreased water permeance of 1.3 liters m^−2^ hour^−1^ bar^−1^. In contrast, a remarkable enhancement in water permeance (6.7 liters m^−2^ hour^−1^ bar^−1^) was observed for PA membranes formed via MCIP, nearly three- or fourfold higher as compared with those from CIP and MLIP, respectively, with satisfactory NaCl rejection. The remarkable improvement in water permeance is attributed to the increased effective surface area and the enlarged internal free volumes that resulted from the crumpled surface structures, as confirmed by both experimental observations (Fig. 4) and simulation results (Fig. 2). Meanwhile, the MCIP PA-TFC membranes outperformed commercial and laboratory-scale RO membranes, transcending the performance upper bound on the salt rejection versus water permeance/salt permeance plot (Fig. 5B). The influences of applied pressure on the water flux and salt rejection were examined using 2000 parts per million (ppm) NaCl solutions for CIP and MCIP TFC membranes. Both membranes showed a nearly linear increase in water flux with an applied pressure increase from 5 to 35 bar, and the MCIP TFC membrane showed higher water fluxes than CIP TFC membranes with similar NaCl rejection throughout the tested pressure range (Fig. 5C). Furthermore, the MCIP membrane exhibited a favorable physicochemical stability and long-term stability (Fig. 5, D and E, and figs. S34 and S35). It confirms that the strategy of incorporating the micellar SDS system into the IP process is beneficial to the generation of crumpled PA TFC membranes with increased effective surface area and enlarged free volume for enhanced water permeation. However, it is not suggested to increase the surfactant concentration in MCIP unlimitedly because an excessive amount of SDS will cause a marked decline in NaCl rejection ratio, though at a higher water permeance (Fig. 5F). It was explained that the formation of micelles created free volume in the PA layer and led to defects after the removal of the surfactant once the IP reaction was terminated (*22*, *23*).

## DISCUSSION

The morphology of PA layers is found to play a critical role in the permeance of PA TFC desalination membranes (*48*), although the precise control of surface morphology relies on in-depth knowledge of the formation mechanisms of membrane surface morphologies and roughness, which is still unclarified. The strategy of incorporating the two thermodynamically different systems, i.e., self-assembly and IP, has proven to be effective for the formation of PA layers in TFC RO membranes with enhanced permeance due to crumpled surface morphologies and enlarged internal free volumes via MCIP method described here. The crumpled nanostructures were irregular as compared with Turing structures reported frequently in high-performance desalination membrane materials (*48*). According to dissipative structure theories, the regular Turing pattern formation in PA layers is generated in thermodynamically nonequilibrium system under specific conditions (*18*). However, irregular crumpled structures have not been thoroughly investigated because of the complexity of the IP processes. Moreover, surfactant has long been regarded as an additive in the IP processes to improve the support wettability to enable more homogeneous distribution and diffusion of amine monomers from the water phase to the organic phase. However, its influence on the water-organic phase interface during IP processes has been ignored. In this work, we found that surfactants serve as not only a facilitator for promoting the wetting of the support membrane but also the initiator for the interfacial instability, which is the key to generate a thin-film MPD-based PA membrane with extensive crumpled nanostructures with unprecedented high permeance without sacrificing high salt rejection. This strategy has been applicable to other IP systems with alternative monomers [e.g., PIP, polyethyleneimine in (*24*), and 1,6-hexanediamine in fig. S36], aromatic organic solvents (e.g., toluene in fig. S37), and surfactants (e.g., CTAB in fig. S38 and Tween 80 in fig. S39), except that the mechanisms of the formation of crumpled nanostructures induced by interfacial instability during the IP process were thoroughly elucidated in this work. Therefore, the universality of this strategy has been validated.

In summary, we investigated the IP process incorporated with the self-assembly surfactant system containing both a monolayer and micelles, obtaining a highly permeable PA layer for RO membranes with roughened surface and enlarged free volume of the internal cavities. The underlying mechanism of the formation of the texture structures was revealed via multiscale (both MD and DPD) simulations. From MD simulations, we found that the thermodynamic stability of the interface that served as the reaction platform plays a key role on the IP process. The thermodynamic fluctuations in the monolayer at the interface, caused by the strong electrostatic repulsion of SDS micelles, lead to structural reorganization of the SDS molecules and determine the initial stage of the formation of the PA layer in the MCIP system. The transformation of the ordered SDS monolayer at the interface into disordered SDS molecules is the main cause of the roughened surface morphology. Furthermore, the results from DPD simulations explain that the enlarged free volume of the internal cavities in MCIP originates from the competitive adsorption of MPD molecules to SDS micelles and to the monolayer. Overall, both experimental and simulation investigations uncovered in this work provide alternative insights into the importance of the thermodynamic state of the interface on the PA membranes and shed light on the fundamental mechanism of the IP process for existing and future desalination membranes. From the viewpoint of practical application, high-performance PA TFC membranes are very promising to address the global challenge of water scarcity.

## MATERIALS AND METHODS

### General

Detailed information on the experimental and simulation sections is provided in the Supplementary Materials.

### Fabrication of PA-TFC membranes

PA-TFC membranes were prepared via IP on a PK substrate, which was prepared by non–solvent-induced phase separation method (*49*, *50*). For MLIP and MCIP, the aqueous solution containing MPD, (±)-10-camphorsulfonic acid (CSA) (4 wt %), triethylamine (TEA) (2 wt %), and SDS with certain concentration was prepared under stirring for 40 min. After that, the as-prepared aqueous solution was gently poured onto the PK substrate and soaked for 3 min, and then the residual aqueous solution was removed by an air knife. Subsequently, the saturated substrate was submerged in a TMC/*n*-hexane solution for 2 min, and then the freshly prepared TFC membrane was treated by a heat curing process at 100°C for 5 min. Last, the TFC membrane was placed into the Milli-Q water (4°C) before further study. The CIP PA TFC membrane was prepared by the aqueous solution without SDS. The IP condition is selected as 0.25 wt % of MPD in the aqueous phase and 0.15 wt % of TMC in the *n*-hexane phase (fig. S33). During the preparation of PA-TFC membranes, unless otherwise specified, all MPD solutions contained 2 wt % TEA and 4 wt % CSA in this work. More details regarding the influence of additives CSA-TEA can be found in the Supplementary Materials (figs. S8 and S40).

### Fabrication of freestanding PA nanofilm

The free-substrate PA nanofilms of three approaches (CIP, MLIP, and MCIP) were fabricated via a support-free IP method (section S1.4) (*51*). The resulting free-substrate PA nanofilms were prepared with identical conditions as the supported PA-TFC membranes but at a free interface, and then transferred onto an AAOI (pore size, 100 nm; Alliance Bio. Com. Japan) membrane filter as the membrane support.

### Characterization methods

The functional groups of different PA TFC membranes were analyzed by FTIR (Spectrum One System, PerkinElmer, USA). Each sample was lyophilized and cut into small pieces and then was detected independently three times with a scan range from 400 to 4000 cm^−1^. The chemical composition of the front or the rear surface of different PA TFC membranes was analyzed independently by XPS (JPS-9010 MC, JEOL, Japan) four times. Carbon, nitrogen, oxygen, and sulfur were detected 10 times in the narrow scan. XPS peak fitting was performed with Spec. Surf. v1.9 software. The surface and cross-sectional morphologies of different samples were characterized by FESEM (JEM 2100 F, JEOL, Japan). A thin layer of osmium tetroxide was sputter-coated onto the surface of the sample before characterization. TEM (JEM 2100 F, JEOL, Japan) was used to observe the cross-sectional morphologies of the different samples. All the samples for TEM measurement were first entrapped in resin and then were allowed to solidify at 60°C for 2 days. After that, the solidified samples were cut into 100-nm-thick slides using a microtome (Ultramicrotome, Leica EM UC7, Germany). The two- and three-dimensional surface microstructures of different PA TFC membranes were investigated by AFM (XE-100, Park System). The water contact angle (surface hydrophilicity) of all the samples was measured with a goniometer (Drop Master 300, Kyowa Interface Science Company, Japan). The surface charge characteristics of different samples were evaluated using a surpass streaming potential analyzer (Anton Paar GmbH, Austria). The surface charge values were measured from pH 9 to 4 using 1 mM KCl solution at 22°C (fig. S41). Free-volume cavity and size distribution of the PA membranes were investigated by PALS (PALS-200A, Toray Research Center, Japan and EG&G Ortec, Chung Yuan University).

### Membrane performance evaluation

Pure water permeance (PWP) and solute rejection (2000 ppm of NaCl) evaluation tests were conducted using a laboratory-scale cross-flow filtration setup with an effective membrane area of 7.4 cm^2^ under an operational pressure of 15.0 bar. Each sample was evaluated independently for at least three times (especially, the PA-CIP and PA-MCIP membranes were repeated five times). All the samples were precompacted at 15 bar for 4 to 6 hours. PWP, *P* (liters m^−2^ h^−1^ bar^−1^), was calculated using Eq. 2

$$P=\frac{\text{Δ}V}{\text{Δ}P\times \text{Δ}t\times A}$$ (2)

where ∆*V* is the collected permeate volume (liters) during a certain time (∆*t*, hours), ∆*P* is the applied pressure (bar), and *A* is the effective permeating area (square meters).

The solute rejection ratio (*R*) was calculated using Eq. 3

$$R=(1-C_{p}/C_{f})\times 100\%$$ (3)

where *C*_f_ and *C*_p_ are the solute concentration of the feed and permeate solutions, respectively, analyzed using a conductivity meter (Ultrameter II 4P, Myron L Company, Japan).

The water permeance (*A*, liters m^−2^ h^−1^ bar^−1^)/NaCl permeance (*B*, liters m^−2^ h^−1^) selectivity (*A*/*B*, bar^−1^) was calculated using Eq. 4

$$A/B=\frac{R}{\left[(1-R)(\text{Δ}P-\text{Δ}\pi )\right]}$$ (4)

where *R* is the NaCl rejection ratio (%), ∆*P* is the applied pressure (bar), and ∆π is the osmotic pressure (bar). For the tests of neutral molecules, the concentrations of neutral molecules in the feed solution and permeate solution were analyzed by the total organic carbon analyzer (TOC-VCSN, Shimadzu Company, Japan).

### Computational simulations

MD simulations were carried out to reveal the interfacial properties during the diffusion process of MPD molecules across the interface in CIP, MLIP, and MCIP systems, respectively. All MD simulations were performed using the Forcite module with the Condensed-phase Optimized Molecular Potential for Atomistic Simulation Studies II (COMPASS II) force field in Materials Studio 2020. A simulation box with periodic boundary conditions applied in all three dimensions was built in this work. The MD models of the water/*n*-hexane interfaces in CIP, MLIP, and MCIP were composed of the same numbers of H_2_O (5000 molecules), MPD (100 molecules), and *n*-hexane (500 molecules) in a rectangular box (*XYZ* parameters: 56 Å × 56 Å × 150 Å) with the interface parallel to the *XY* plane (fig. S3). A monolayer composed of 42 uniformly distributed SDS molecules was placed at the water/*n*-hexane interface in both MLIP and MCIP systems. An SDS micelle containing 50 SDS molecules with a radius of 15 Å underneath the SDS monolayer with a distance of 25 Å was constructed in the MCIP system. The numbers of SDS molecules in the monolayer and in the micelle, as well as the distance between the monolayer and the micelle, is consistent with the literature (*7*, *25*, *52*). By appropriately setting the positions of all molecules, a geometry optimization process was first performed, followed by a dynamic run for 20 ps with constant number of molecules, volume, and temperature (NVT ensemble) at 298 K. To ensure that all systems have reached equilibrium, another run for 20 ps with constant number of molecules, volume, and energy (NVE ensemble) followed by 20 ps with NVT ensemble was applied, and the energy as well as temperature of all systems reached the steady values. The simulation results of the final 10 ps run in NVT were used for data analysis.

To clarify the heating effects, the monomer-free CIP, MLIP, and MCIP models were simulated with NVT thermodynamic ensemble at 298 and 318 K, respectively. The configurations and the thermodynamic properties of the interfaces were captured and analyzed after 20 ps when the systems reached the steady state.

The diffusion coefficients (*D*, m^2^ s^−1^) of MPD and SDS molecules in different systems were analyzed from the slope of MSD curves by Einstein relationship (*53*)

$$\mathrm{MSD}=\frac{1}{N}\sum_{1}^{N}\{{\left[r\;(t)-r\;(0)\right]}^{2}\}$$ (5)

$$D=\frac{1}{6}\lim_{N\to \infty }\frac{d}{dx}\{{\left[r\;(t)-r\;(0)\right]}^{2}\}$$ (6)

where *N* is the total number of targeted molecules (i.e., MPD or SDS herein this work) and *r* (0) and *r* (*t*) represent the initial position (m) and position at *t* (m) of the targeted molecule, respectively.

Specifically, the adsorption energy of a single molecule of MPD onto the SDS monolayer and/or the SDS micelle was obtained using the Adsorption Locator module in Materials Studio 2020. An SDS monolayer (containing 42 uniformly distributed SDS molecules) parallel to the *XY* plane was built in the rectangular box (*XYZ* parameters: 56 Å × 56 Å × 150 Å) to calculate the adsorption energy of a single molecule of MPD at different positions in the MLIP system. An additional SDS micelle composed of 50 SDS molecules with the same monolayer structure as in the MLIP system was constructed in the MCIP system. The head groups of SDS molecules were set as the surface region, and the MPD molecule was set as the adsorbate. A maximum adsorption distance of 10 Å was selected for the adsorption calculation between the MPD molecule and SDS molecules (in monolayer and/or micelles). The force field COMPASS II was also applied in the calculation and analysis of the energetic properties of the MPD. The negative binding energy usually indicates an energetically favorable adsorption between the adsorbate and the target surface.

DPD simulations were carried out to investigate the IP process of MPD and TMC monomers at the water/*n*-hexane interface (*54*). Five different components were modeled in the IP system: water, MPD, SDS in the water phase, *n*-hexane, and TMC in the organic phase. The coarse graining of all molecules into DPD beads (fig. S5) and the repulsion parameters between DPD beads (table S4) were calculated from Flory-Huggins parameters as reported previously (*54*, *55*). In the CIP system, a cubic simulation box of 100 Å × 100 Å × 100 Å was constructed and divided into two slabs (100 Å × 100 Å × 50 Å). In the upper slab, a layer of TMC beads mixed with *n*-hexane beads (*n*-hexane/TMC = 0.925:0.075) was introduced as the oil phase. In the water phase, water and MPD beads (water/MPD = 0.925:0.075) were added. In particular, the SDS monolayer (composed of 102 SDS molecules in the middle of the simulation box, slab dimensions: 100 Å × 100 Å × 20 Å) was included perpendicular to the z axis in MLIP and MCIP box (lattice parameters, 100 Å × 100 Å × 120 Å). Moreover, an SDS micelle was contained in the water phase in the MCIP system, with a radius of 15 Å. The density of the whole system is 3.0 in reduced units. Each slab was independently equilibrated before the system was assembled for simulations. After geometry optimization, an initial period of 10,000 steps was carried out for equilibrating the system, and the simulation productions were performed for an additional 50,000 steps (~10 ns). The cross-sectional configurations of the IP interface were extracted on the last 20,000 (~4 ns) steps at every 500 steps.

## Supplementary Materials

### This PDF file includes:

Supplementary Text Figs. S1 to S41 Tables S1 to S4 References
