# Supplementary material for: When self-assembly meets interfacial polymerization
Source: Sci Adv. 2023 May 3;9(18):eadf6122. doi: 10.1126/sciadv.adf6122 (PMC10156122; doi:10.1126/sciadv.adf6122)
Supplement: Supplementary file 1 — Supplementary Text Figs. S1 to S41 Tables S1 to S4 References [file sciadv.adf6122_sm.pdf]

Science Advances
AAAS

Supplementary Materials for

When self-assembly meets interfacial polymerization

Qin Shen *et al.*

Corresponding author: Zhaohuan Mai, zhaohuan.mai@people.kobe-u.ac.jp;
Hideto Matsuyama, matuyama@kobe-u.ac.jp

Sci. Adv. **9**, eadf6122 (2023)
DOI: 10.1126/sciadv.adf6122

This PDF file includes:

Supplementary Text
Figs. S1 to S41
Tables S1 to S4
References

Supplementary Text

1. Experimental section

1.1. Materials and chemicals

Polyketone (PK, molecular weight: 200 k g mol^{-1}) was provided by Asahi Kasei Company, Japan. The ethanol (EtOH, 99.5%), sodium chloride (NaCl, 99.0%), N, N-dimethylformamide (DMF, 99.5%), sodium dodecyl sulfate (SDS, 99.0%) were purchased from FUJIFILM Wako Pure Chemical Company, Japan. Resorcinol, triethylamine (TEA, >99.0%), cetyltrimethylammonium bromide (CTAB, 98.0%), Tween 80, anhydrous *n*-hexane (99.0%), and (±)-10-camphorsulfonic acid (CSA, >98.0%) were purchased from Tokyo Chemical Industry, Japan, and 1, 3-phenylene diamine (MPD, 99.0%), 1, 3, 5-benzenetricarbonyl trichloride (TMC, 98.0%) were purchased from Sigma-Aldrich, USA.

1.2. Preparation of PK support and different polyamide (PA) TFC membranes

PA membranes were prepared by interfacial polymerization (IP) on a PK substrate, which was prepared via the non-solvent induced phase separation method (*50*). The aqueous solution containing MPD, CSA (4 wt%), TEA (2 wt%), and SDS with certain concentration was prepared under stirring for 40 min. After that, the as-prepared aqueous solution was gently poured onto the PK substrate and soaked for 3 min, and then the residual aqueous solution was removed by an air knife. Subsequently, the saturated substrate was submerged in a TMC/*n*-hexane solution for 2 min, and then the freshly-prepared TFC membrane was treated by a heat curing process at 100 °C for 5 min. Finally, the TFC membrane was soaked into the Milli-Q water (4 °C) for further study. The conventional IP (CIP) fabricated PA TFC membrane was prepared by the aqueous solution without SDS. The abbreviation MLIP indicates that the PA membrane was fabricated in the IP process with the addition of SDS in the form of a monolayer (with a concentration below or equivalent to its critical micelle concentration, CMC). Moreover, MCIP represents the IP process with SDS in the form of both a monolayer at the water/*n*-hexane interface and micelles in the aqueous solution (with a concentration above its CMC).

1.3. Measurement of MPD diffusion rate from aqueous phase to organic phase

First, the aqueous solution (30 mL) containing MPD (0.25 wt%), CSA (4 wt%), TEA (2 wt%), and SDS with certain concentration was poured into a beaker (100 mL), and then *n*-hexane (30 mL) was gently injected to the surface of the aqueous phase. After 120 s, *n*-hexane (~3 mL) was slightly

collected from the air/*n*-hexane interface and placed into the disposable cell (4.5 mL, 2-478-06, AS ONE Corporation, Japan). The absorbance variation of MPD (maximum absorption peak: ~252.1 nm) in *n*-hexane was detected by ultraviolet–visible (UV–vis) spectroscopy (V-650KE, JASCO Company, Japan). Then, the absorbance variation of MPD with time was analyzed by the fixed-wavelength UV–vis absorption spectra (Wavelength: ~252.1 nm). The as-prepared aqueous solution (1 mL) was gently injected into a 4.5-mL disposable cell. The height of aqueous solution (~1 cm) should remain below the light path of the spectrometer. And then *n*-hexane (1.5 mL) was slowly added on the surface of aqueous phase within 15 s. The diffused amount of MPD into *n*-hexane phase during 15 to 120 s was collected by UV–vis absorption spectroscopy (*45*).

1.4. Fabrication of free-substrate PA nanofilm

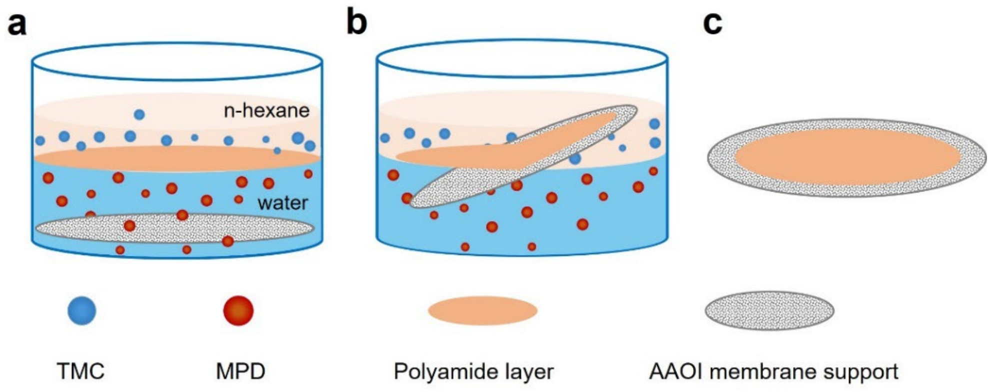

Fig. S1. Schematic of freestanding PA nanofilms fabricated at the free interface. (a) PA film formed at the water/*n*-hexane interface; (b) PA film transferred onto anodic aluminium oxide isotropic (AAOI) support; (c) Drying of PA film on AAOI support.

The free-substrate PA nanofilms of three approaches (CIP, MLIP, and MCIP) were fabricated via a support-free IP method (**Fig. S1**) (*51*). The resulting free-substrate PA nanofilms were prepared with identical conditions at a free interface, and then transferred onto an anodic aluminium oxide isotropic (AAOI, Pore size 100 nm, Alliance Bio. Com. Japan) as the membrane support.

1.5. Exfoliating PA active layer from substrate

As shown in **Fig. S2**, the newly fabricated PA TFC membranes were carefully fixed on a silicon wafer (2-960-02, AS ONE Corporation, Japan) by reverse-action tweezers, where the PA layer was placed on the wafer surface. Then, the fixed sample was completely immersed in 150 ×

3 mL organic solution for 12 h to completely dissolve the interbedded polymer support. Subsequently, 20 mins after the tweezers were removed, the nonwoven fabric floated and then was detached from the PA active layer. Finally, the exfoliated sample was dried at room temperature in air for 2–3 days prior to characterization (Supplementary Materials 1.6.2).

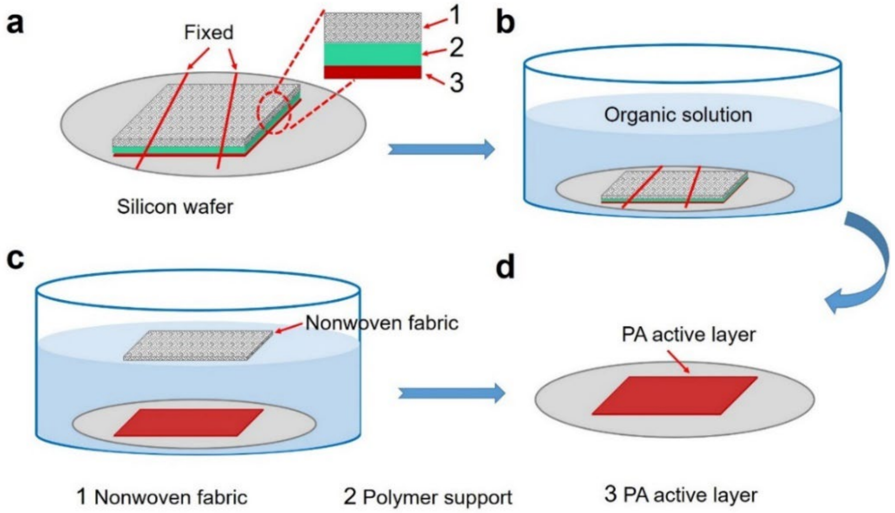

Fig. S2. **Schematic of exfoliating the PA active layer from the substrate.** (a) PA TFC membrane fixed on a silicon wafer; (b) The fixed sample immersed in organic solution to dissolve the polymer support; (c) Nonwoven fabric detached from the PA active layer; (d) PA active layer dried in air.

1.6. Characterization methods

1.6.1. Fourier transform infrared spectrometry (FTIR)

The functional groups of different PA TFC membranes were analyzed by FTIR (Spectrum One System, Perkin-Elmer, USA). Each sample was lyophilized and cut into small pieces, and then was detected independently for 3 times with the scan range from 400 to 4000 cm^{-1}.

1.6.2. X-ray photoelectron spectroscopy (XPS)

The chemical compositions of the front and/or the rear surfaces of the exfoliated PA active layers from different TFC membranes was analyzed independently by XPS (JPS-9010 MC, JEOL, Japan) for 4 times. Carbon, nitrogen, oxygen, sulfur elements were detected for 10 times in the narrow scan. XPS peak fitting was performed with Spec. Surf. V 1.9 software.

1.6.3. Field emission scanning electron microscopy (FESEM)

The surface and cross-sectional morphologies of different samples were characterized by FESEM (JEM 2100 F, JEOL, Japan). A thin layer of osmium tetroxide was sputter-coated onto the surface of the sample prior to characterization.

1.6.4. Transmission electron microscopy (TEM)

TEM (JEM 2100 F, JEOL, Japan) was used to observe the cross-sectional morphologies of the different samples. All the samples for TEM measurement were first entrapped in resin and then were allowed to solidify at 60 °C for 2 days. After that, the solidified samples were cut into 100 nm-thickness slides by using a microtome (Ultramicrotome, Leica EM UC7, Germany).

1.6.5. Atomic force microscope (AFM)

The two- and three-dimensional surface microstructures of different PA TFC membranes were investigated by AFM (XE-100, Park System).

1.6.6. Water contact angle measurement

The water contact angle (surface hydrophilicity) of all the samples was measured by a goniometer (Drop Master 300, Kyowa Interface Science Company, Japan).

1.6.7. Membrane surface charge measurement

The surface charge characteristics of different samples were evaluated using a surpass streaming potential analyzer (Anton Paar GmbH, Austria). The surface charge values were measured from pH 9 to 4 using 1 mM KCl solution at 22°C.

1.6.8. Interfacial tension measurement

The interfacial tension between *n*-hexane and MPD aqueous solution with different SDS concentrations was evaluated using the Du Noüy ring method with Sigma tensiometer system (Sigma 700, Biolin Scientific, Sweden). Firstly, 20 ml MPD aqueous solution was added into disposable transparent container as heavy phase, and then *n*-hexane was slightly poured onto the MPD aqueous solution as light phase. The Du Noüy ring was rinsed by EtOH and *n*-hexane, respectively, and then cauterized by flame (~1300 °C) for 60 s to completely remove the contaminants on its surface. The ring was brought close to the interface but still kept into the heavy phase, and then slightly elevated by Sigma tensiometer across the interface and into the light phase.

The data were collected 5 times and processed by computer. Each sample was repeated independently for 3 times.

1.6.9. Micelle size distribution measurement

The size distribution of micelles in the surfactant solution were evaluated by dynamic light scattering (DLS; ELSZ-1000ZA, Otsuka Electronics, Japan).

1.6.10. Positron annihilation lifetime spectroscopy (PALS)

Free-volume cavity and size distribution of the PA membranes were investigated by PALS (PALS-200A, Toray Research Center, Japan). The radioactive source ^{22}Na (18 μCi) was sandwiched between two Kapton foils with a time resolution of 278-280 ps at room temperature. PALS data were recorded with total counts of 5 million. In this work, the free-volume voids are assumed as spherical, and the average vacancy volume size (V_a) of the free-volume voids can be estimated by the following equation (*56*):

$$V_a = \frac{4}{3}\pi r^3 \quad \text{(S1)}$$

A semi-empirical equation based on the spherical-cavity model of the *o*-Ps lifetime was suggested to correlate the *o*-Ps (τ_i, ns) lifetime with the free volume radius (r, Å) in the PA active layer as follows (*57, 58*):

$$\tau_i = 0.5\left[1 - \frac{r_i}{r_i+1.66} + \frac{1}{2\pi}\sin\left(\frac{2\pi r_i}{r_i+1.66}\right)\right]^{-1};\ i = 2, 3 \quad \text{(S2)}$$

The fractional free volume (FFV) of the PA active layer is calculated according to the Williams-Landel-Ferry (WFL) equation (*59*):

$$FFV = \sum_i 0.00181 I_i \left\langle V_f\left(\frac{4}{3}\pi r_i^3\right)\right\rangle \ ;\ i = 2, 3 \quad \text{(S3)}$$

where I_i (%) is the *o*-Ps intensity of the estimated pick-off lifetime τ_i, and V_f represents the mean free volume (Å^3) of the cavity in the PA active layer calculated by the mean free volume radius r.

The Doppler energy spectroscopy (DBES) spectra were determined using PALS with a variable monoenergy slow positron beam (0-5 k eV) and recorded using an HP Ge detector (EG&G Ortec, R&D Center for Membrane Technology Chung Yuan University, Taiwan). The positron source is of a 50 mCi of ^{22}Na radioisotope beam. The S parameter was reported from the DBES measurement. The S parameter, which was from the o-Ps 2g pick-off annihilation in free volume,

yielded information about the depth profile of the free volume (Å to nm) in the polyamide layer (*25, 60*).

1.7. Evaluation of membrane filtration property

Pure water permeance and solute rejection (2000 ppm NaCl) evaluation tests were conducted by using a lab-scale cross-flow filtration setup with a 7.4-cm^2 effective permeating area under an operational pressure of 15.0 bar. Each sample was evaluated independently for at least 3 times (especially, the PA-CIP and PA-MCIP membranes were repeated for 5 times). All the samples were pre-compacted at 15 bar for 4-6 h. The pure water permeance (PWP), *P* (L m^{-2} h^{-1} bar^{-1}), was calculated using Equation S4:

$$P = \frac{\Delta V}{\Delta P \times \Delta t \times A} \quad \text{(S4)}$$

where ΔV is the collected permeate volume (L) during a certain time (Δt, h); ΔP is the applied pressure (bar); and A is the effective permeating area (m^2).

The solute rejection ratio (*R*) was calculated using Equation S5:

$$R = \left(1 - C_p/C_f\right) \times 100\% \quad \text{(S5)}$$

where C_f and C_p are solute concentration of the feed and permeate solution, respectively, which were analyzed using a conductivity meter (Ultrameter II^{TM} 4P, Myron L Company, Japan).

For the tests of neutral molecules, the concentrations of neutral molecules in the feed solution and permeate solution were analyzed by the total organic carbon analyzer (TOC-VCSN, Shimadzu Company, Japan).

1.8. Crosslinking degree of PA TFC membranes

The crosslinking degree of the TFC membranes was investigated by calculating the atomic concentrations at the membrane surface using Equation S6 (*61, 62*).

$$Crosslinking\ degree\ (\%) = \frac{m}{m+n} \times 100 \quad \text{(S6)}$$

where the values of *m* and *n* were calculated from the O/N ratio from the XPS data using Equations S7 and S8:

$$m + n = 1 \quad \text{(S7)}$$

$$\frac{O}{N} = \frac{3m + 4n}{3m + 2n} \tag{S8}$$

2. Computational simulations

2.1 Molecular dynamics (MD) simulations

2.1.1 Thermodynamic properties of the interface during the diffusion of MPD

The diffusion-reaction IP process was decoupled in MD simulations, and for simplicity, we investigated the interfaces of the three systems with 3 models containing no TMC molecules. MD simulations were carried out to reveal the interfacial properties during the diffusion process of MPD molecules across the interface in CIP, MLIP, MCIP systems, respectively. All MD simulations were performed using the Forcite module with the Condensed-phase Optimized Molecular Potential for Atomistic Simulation Studies II (COMPASS II) force field in Materials Studio 2020. A simulation box with periodic boundary conditions applied in all three dimensions was built in this work. The MD models of the water/ *n*-hexane interfaces in CIP, MLIP and MCIP were composed of the same numbers of H_2O (5000 molecules), MPD (100 molecules) and *n*-hexane (500 molecules) in a rectangular box (*XYZ* parameters: 56 × 56 × 150 Å^3, the vacuum above the *n*-hexane phase was introduced to avoid the interaction between the *n*-hexane molecules and the periodic image of the bottom layer of water molecules in the surface) with the interface parallel to the *XY* plane (**Fig. S3**). In particular, a monolayer comprised of 42 uniformly distributed SDS molecules was placed at the water/*n*-hexane interface in both MLIP and MCIP systems. In addition, an SDS micelle containing 50 SDS molecules with a radius of 15 Å underneath the SDS monolayer with a distance of 25 Å were constructed in MCIP system. The numbers of SDS molecules in the monolayer and in the micelle, as well as the distance between the monolayer and the micelle, is consistent with literature (*7, 25, 52*). By appropriately setting the positions of all molecules, a geometry optimization process was first performed, followed by a dynamic run for 20 ps with NVT (constant number of molecules, volume and temperature) ensemble at 298 K. To make sure all systems have reached equilibrium, another run for 20 ps with NVE (constant number of molecules, volume and energy) ensemble followed by 1 ns with NVT ensemble were applied, and the energy as well as temperature of all systems reached the steady values. The simulation results of the final 20 ps run in NVT were used for data analysis.

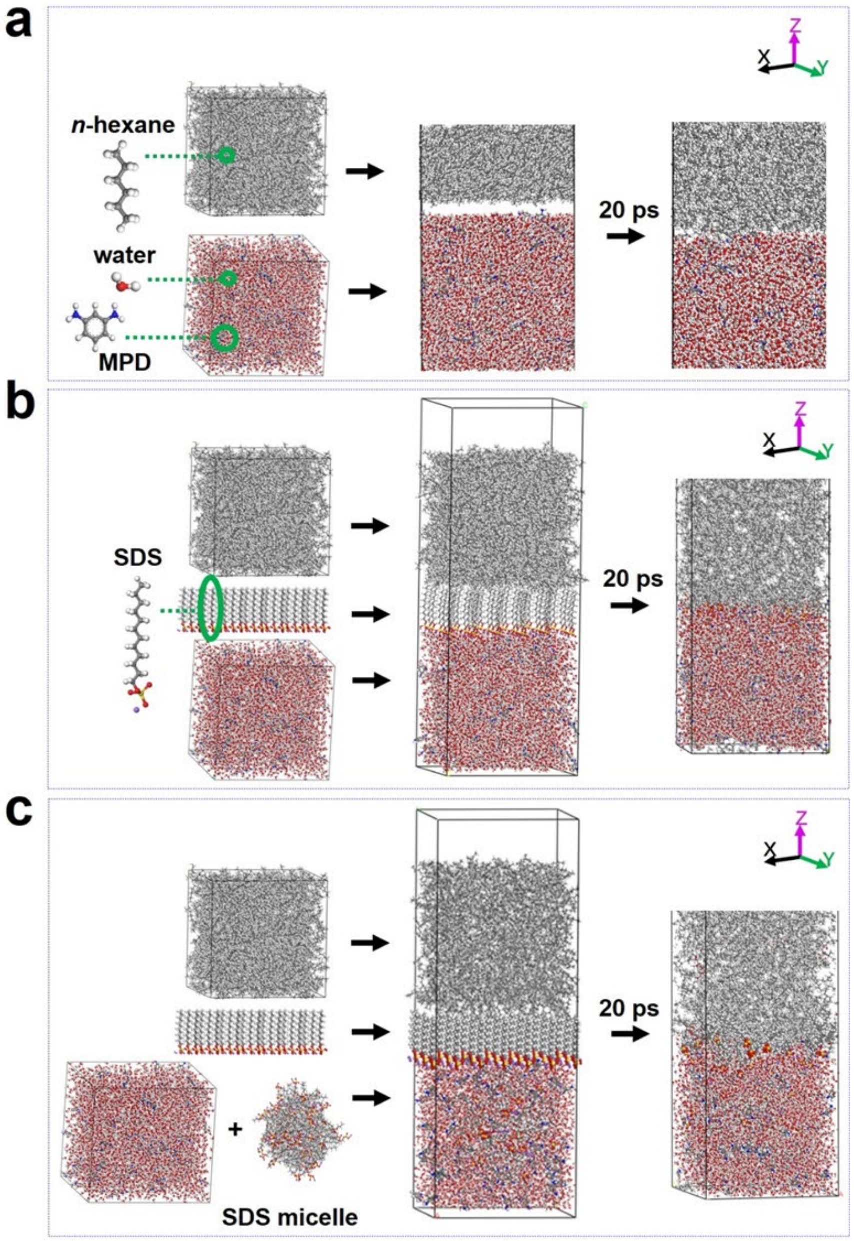

Fig. S3. Construction and equilibration of MD models. (a) CIP; (b) MLIP; (c) MCIP. White, grey, blue, red and yellow balls represent H, C, N, O and S atoms, respectively. Areal density of SDS monolayer at the water/*n*-hexane interface: 1.1 molecule nm^{-2}. Radius of SDS micelle in the aqueous solution: 1.5 nm (50 SDS molecules). Water phase is at the bottom of the box, and *n*-hexane phase is on the upper layer. The *Z*-axis is perpendicular to the interface.

The water/*n*-hexane interfaces in the three MD systems were determined at around 60 Å in the *Z* direction with two different definitions. For CIP system, the interface was determined by the minimum density of O atoms in water molecules. For MLIP and MCIP systems, the crest of S atoms concentration could be considered as the interface due to the presence of the monolayer.

The diffusion coefficients (D, m^2 s^{-1}) of MPD and SDS molecules in different systems were analyzed from the slope of mean square displacement (*MSD*) curves by Einstein relationship (*53*):

$$MSD = \frac{1}{N}\sum_{1}^{N}\{[r\ (t) - r\ (0)]^2\} \tag{S9}$$

$$D = \frac{1}{6}\lim_{N\to\infty}\frac{d}{dx}\ \{[r\ (t) - r\ (0)]^2\} \tag{S10}$$

where N is the total number of targeted molecules (i.e., MPD or SDS in this work), r (0) and r (t) represent the initial position (m) and position at t (m) of the targeted molecule.

2.1.2 Interactions between MPD and SDS molecules

As the interactions between SDS and MPD molecules include electrostatic interactions and hydrogen bonding, we used adsorption/binding energy to represent their interactions. Specifically, the adsorption energy of a single molecule of MPD onto SDS monolayer and/or the SDS micelle was obtained using Adsorption Locator module in Materials Studio 2020. The models in both MLIP and MCIP systems were constructed without water and *n*-hexane, as presented in **Fig. S4**. An SDS monolayer (containing 42 uniformly distributed SDS molecules) parallel to the *XY* plane was built in the rectangular box (*XYZ* parameters: 56 × 56 × 150 $Å^3$) to calculate the adsorption energy of a single molecule of MPD at different positions in the MLIP system. An additional SDS micelle composed of 50 SDS molecules with the same monolayer structure as in the MLIP system was constructed in the MCIP system. The head groups of SDS molecules were set as the surface region and the MPD molecule was set as the adsorbate. A maximum adsorption distance of 10 Å was selected for the adsorption calculation between the MPD molecule and SDS molecules (in monolayer and/or micelles). The force field COMPASS II was also applied in the calculation and analysis of the energetic properties of the MPD. The negative binding energy usually indicates an energetically favorable adsorption between the adsorbate and the target surface.

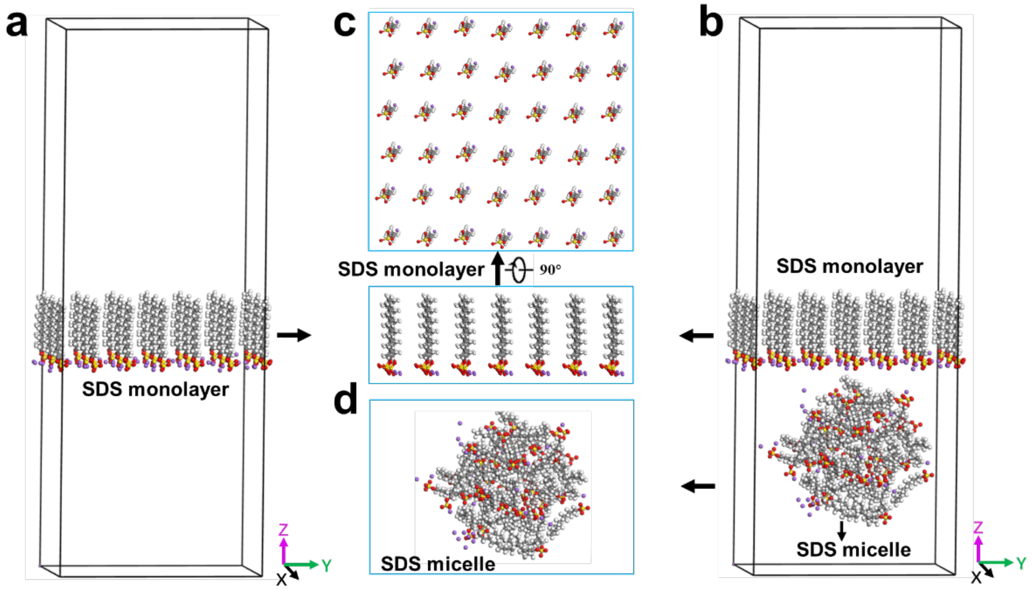

Fig. S4. Construction of the MPD adsorption models. (a) MLIP system; (b) MCIP system; (c) SDS monolayer; (d) SDS micelle. The *Z*-axis is chosen to be perpendicular to the monolayer interface.

2.1.3 Effect of heating on thermodynamic properties of the interface

The heat released during IP process has been measured with a microfluidic methodology and the temperature generated near the reaction zone was reported to achieve as high as 80 °C in MPD-TMC based IP system (*63*). In this work, heating effect was explored by simply investigating the interfacial properties of water/*n*-hexane interface with and without SDS. Therefore, no MPD and TMC molecules were included in this part. The construction of SDS monolayer in MLIP as well as SDS micelle in MCIP system, and MD simulations were performed similarly as described in Supplementary **Section 2.1.1** and **Fig. S3**, except that the MD models were constructed without MPD molecules. To clarify the heating effects, the monomer-free CIP, MLIP and MCIP models were simulated with NVT thermodynamic ensemble at 298 K and 318 K, respectively. The configurations and the thermodynamic properties of the interfaces were captured and analyzed after 20 ps when the systems reached the steady state.

2.2 Dissipative particle dynamics (DPD) simulations

DPD method is a mesoscopic simulation method and can provide thermodynamic properties of the system with a larger spatial scale and longer time scale, compared to MD simulation method. The theories of DPD simulations can be found in the literature (*39, 64*). In this work, DPD simulations were carried out to investigate the IP process of the MPD and TMC monomers at the water/*n*-hexane interface [13]. Five different components were modeled in the IP system: water, MPD, SDS in water phase, and *n*-hexane, TMC in organic phase. The coarse-graining of all molecules into DPD beads **(Fig. S5)** and the repulsion parameters between DPD beads **(Table S4)** were calculated from Flory-Huggins parameters as reported previously (*54*). In CIP system, a cubic simulation box of 100 × 100 × 100 $Å^3$ was constructed and divided into two slabs (100 × 100 × 50 $Å^3$) representing the oil phase and the water phase, respectively. In the upper slab, a layer of TMC beads mixed with *n*-hexane beads (*n*-hexane/TMC = 0.925: 0.075) were introduced as the oil phase. In the water phase, water and MPD beads (water/MPD = 0.925: 0.075) were added. In particular, the SDS monolayer (composed of 102 SDS molecules in the middle of the simulation box, slab dimensions: 100 × 100 × 20 $Å^3$) was included perpendicular to *Z*-axis in MLIP and MCIP box (lattice parameters 100 × 100 × 120 $Å^3$). Moreover, an SDS micelle was contained in water phase in MCIP system, with a radius of 15 Å. The density of the whole system is 3.0 in reduced units. Each slab was independently equilibrated before the system was assembled for simulations.

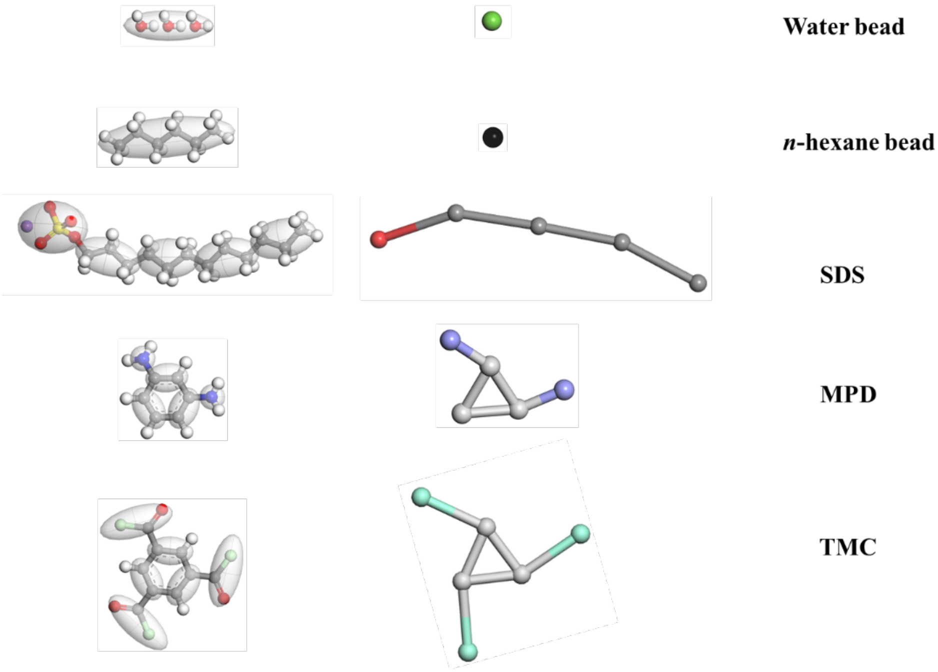

Fig. S5. Coarse-graining of different molecules (water, *n*-hexane, SDS, MPD and TMC) into DPD bead based meso-molecules.

After geometry optimization, an initial period of 10 000 steps was carried out for equilibrating the system, and the simulation productions were performed for an additional 50 000 steps (~10 ns). The cross-sectional configurations of IP interface were extracted on the last 20 000 (~ 4 ns) steps at every 500 steps.

In most cases, DPD methods can provide qualitative description rather than quantitative analysis of the system, since the chemical bond formation could not be depicted (*54*). Therefore, the amide bond formation is represented by the close contact of the amino beads in MPD molecules and benzoyl chloride beads in TMC molecules.

Supplementary Figures

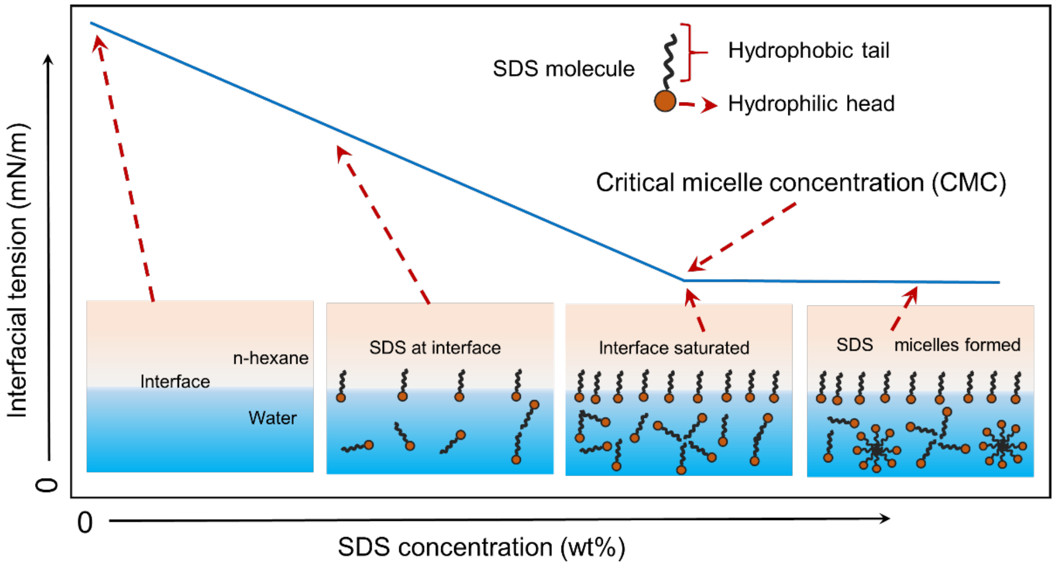

Fig. S6. Interfacial tension and surfactant states with increasing its concentration.

As shown in **Fig. S6**, surfactant molecules tend to arrange themselves at the biphasic interface at low concentrations. As more surfactant molecules were added, interfacial tension can gradually decrease, until the interface is saturated with surfactant molecules (monolayer) and the interfacial tension has reached its maximal reduction. At this point, the interface has become so crowded that the surfactant molecules have to arrange themselves into micelles in the bulk solution. The surfactant concentration at this point is called the critical micelle concentration (CMC), above which any further additions have to arrange as micelles in the bulk solution (*55*).

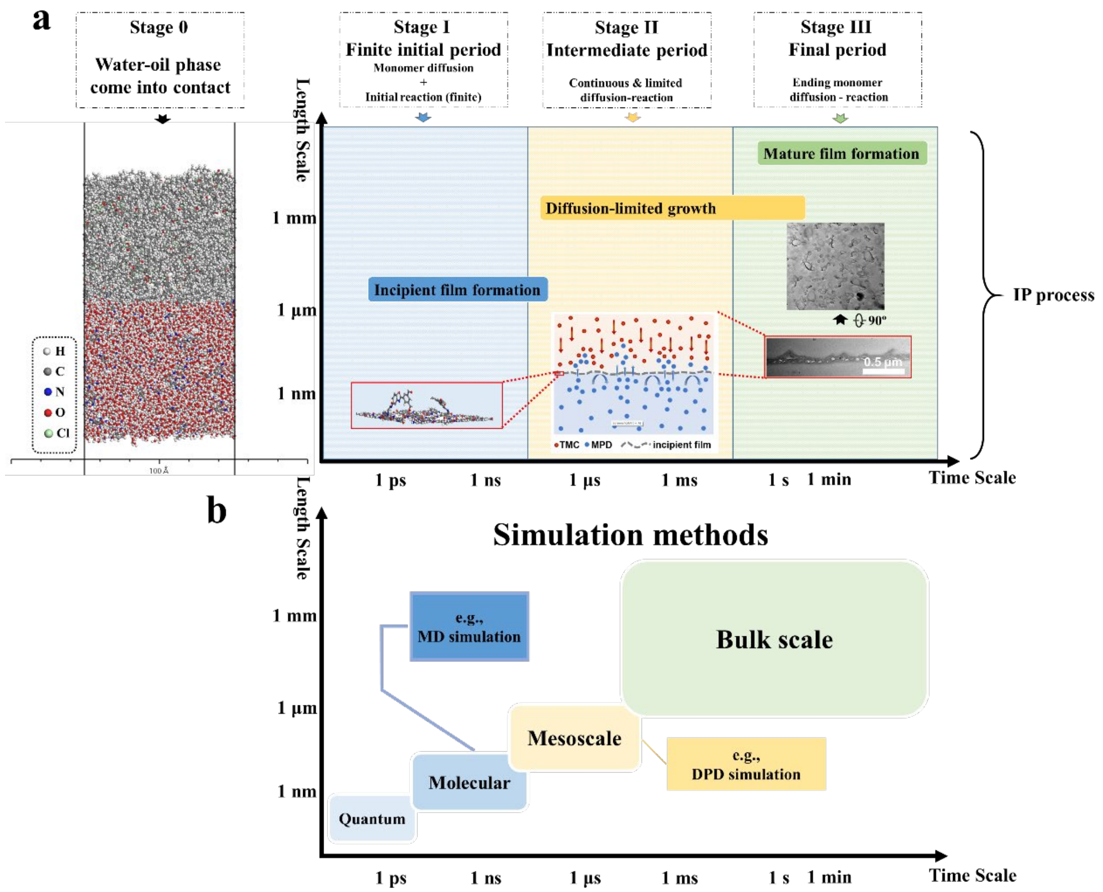

Fig. S7. **Incorporation of time and length scales.** (a) PA membrane formation process; (b) Different simulation methods.

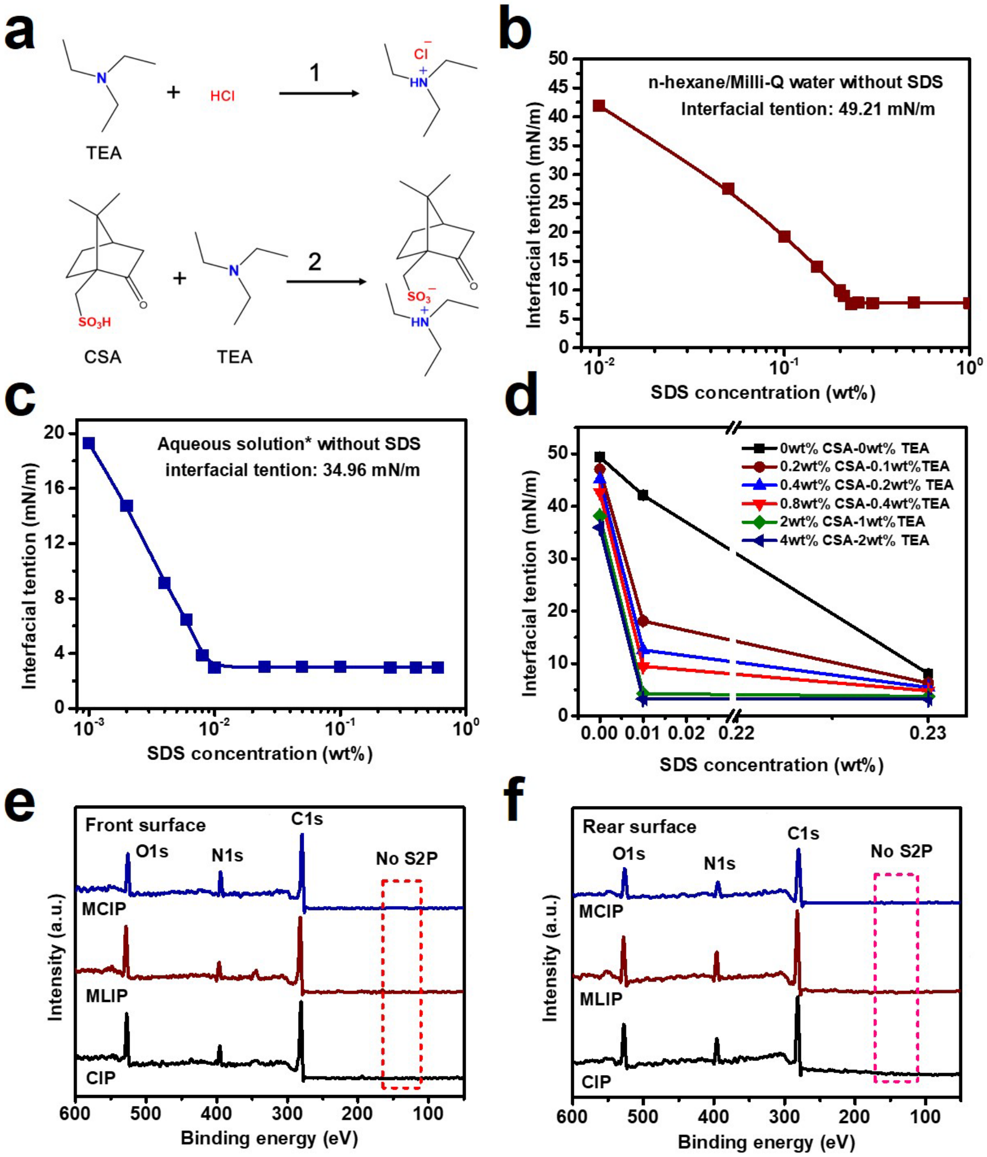

Fig. S8. Determination of CMC for SDS in different systems and elemental analysis of CIP, MLIP and MCIP membranes. (a) The underlying interactions: (1) between TEA and HCl generated as byproduct during IP process, and (2) between TEA and CSA; (b) Interfacial tension between *n*-hexane and Milli-Q water with different concentrations of SDS; (c) Interfacial tension between *n*-hexane and aqueous solution (including 2 wt% TEA, 4 wt% CSA, and 0.25 wt% MPD) with different concentrations of SDS; (d) Interfacial tension between *n*-hexane and SDS aqueous solution (including 0.25 wt% MPD) with different concentrations of CSA and TEA; full-scan XPS spectra of the (e) front and (f) rear surfaces of CIP, MLIP, and MCIP membranes.

The TEA, CSA, and TEA-CSA organic salt additives have been explored in numerous previous studies about RO membrane, but their effects on interfacial polymerization are controversial (*47, 65, 66*). In this work, the additives used in aqueous solution were 2 wt% TEA

and 4 wt% CSA, respectively. In our opinion, TEA has two effects in interfacial polymerization process: 1) TEA (2.6 mmol, 12.9% of the total, **Fig. S8a**) acts as a catalyst, accelerating the TMC–MPD polymerization reaction by neutralizing HCl generated during amide bound formation (*66*), 2) TEA (17.2 mmol, 87.1% of total, **Fig. S8a**) forms organic salt between its amine group and the sulfonic group of CSA, indicating the resultant TEA-CSA organic salt can affect the CMC of SDS. As shown in **Fig. S8b**, the interfacial tension-concentration curve indicates that the CMC of the SDS surfactant in the Milli-Q water is about 0.23 wt% (8.1 mmol L-1, 25 °C), which is approximate to the values reported by previous studies (*67, 68*). However, the CMC of the SDS in the TEA-CSA aqueous solution will dramatically decrease from ~ 0.23 wt% to ~ 0.01 wt% (**Fig. S8c**). As shown in **Fig. S8d**, the CMC of the SDS gradually decreases with increasing the concentration of the resultant TEA-CSA, which may be due to the counter-ion effect (*55, 69*). The counter-ion effect weakens the electrostatic repulsion between the negative-charge SDS molecules, reducing the free energy of micellization (ΔG_m^0<0). As a result, more SDS micelles with lower energy will generate and modulate the interfacial polymerization compared with those in the system without the counter ions. In the case of CSA, CSA can react with TEA to form the organic salt, which is believed to help increase the porosity of the PA selective layer (*70*). In addition to this effect, TEA-CSA organic salts can affect the CMC of SDS (referring to the effect of TEA).

Notably, TEA molecules don't participate in the reaction with TMC molecules during the interfacial polymerization due to lacking an active amino group in its structure (**Fig. S8e**). In addition, the XPS results indicate there is no obvious S element signal on the front and rear sides of the exfoliated PA active layer (**Fig. S8f**), confirming our hypothesis that CSA molecules also are not involved in the reaction with TMC molecules during the interfacial polymerization.

Therefore, in this work, we used 0.01 wt% SDS solution containing CSA-TEA for the MLIP process, considering this concentration as the CMC of the mixed surfactant solution, and 0.4 wt% SDS in the CSA-TEA aqueous solution for the MCIP process. The SDS solutions referred hereafter are mixed solutions with SDS and CSA-TEA. The micelle size distribution of different SDS solutions are presented in **Fig. S9**. It could be found that all SDS micelles are with a size below 10 nm.

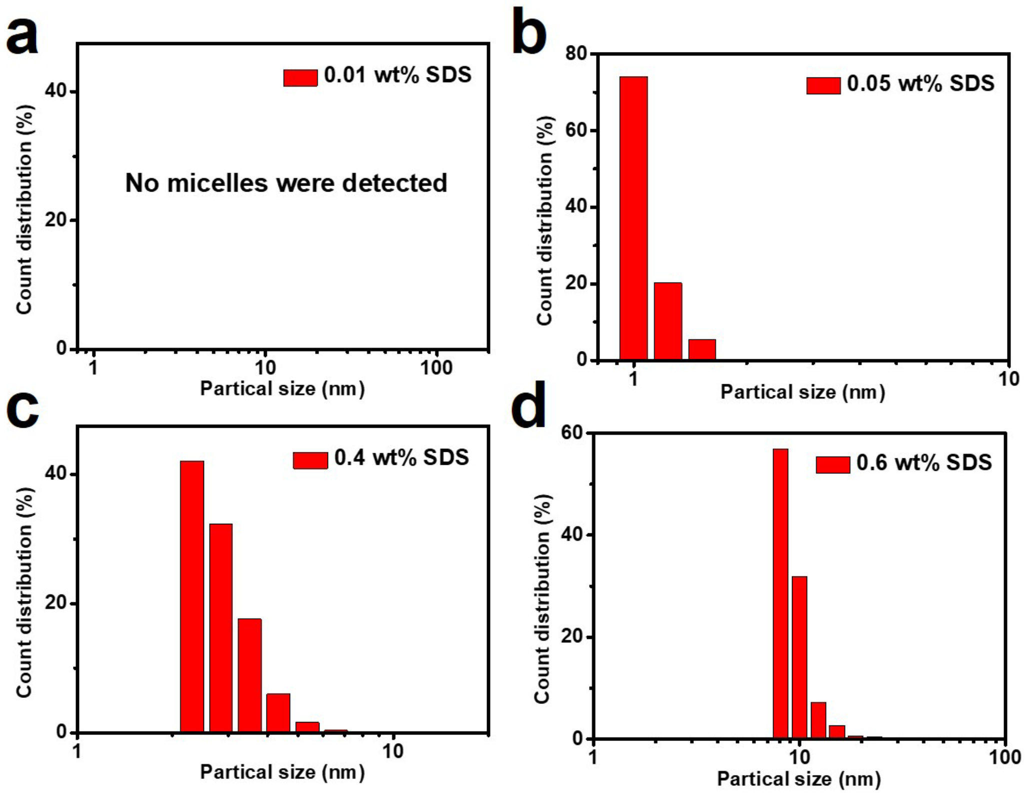

Fig. S9. Micelle size distribution of different SDS solutions (containing 4 wt% CSA and 2 wt% TEA) obtained from DLS characterization. (a) 0.01 wt% SDS; (b) 0.25 wt% SDS; (c) 0.4 wt% SDS; (d) 0.6 wt% SDS.

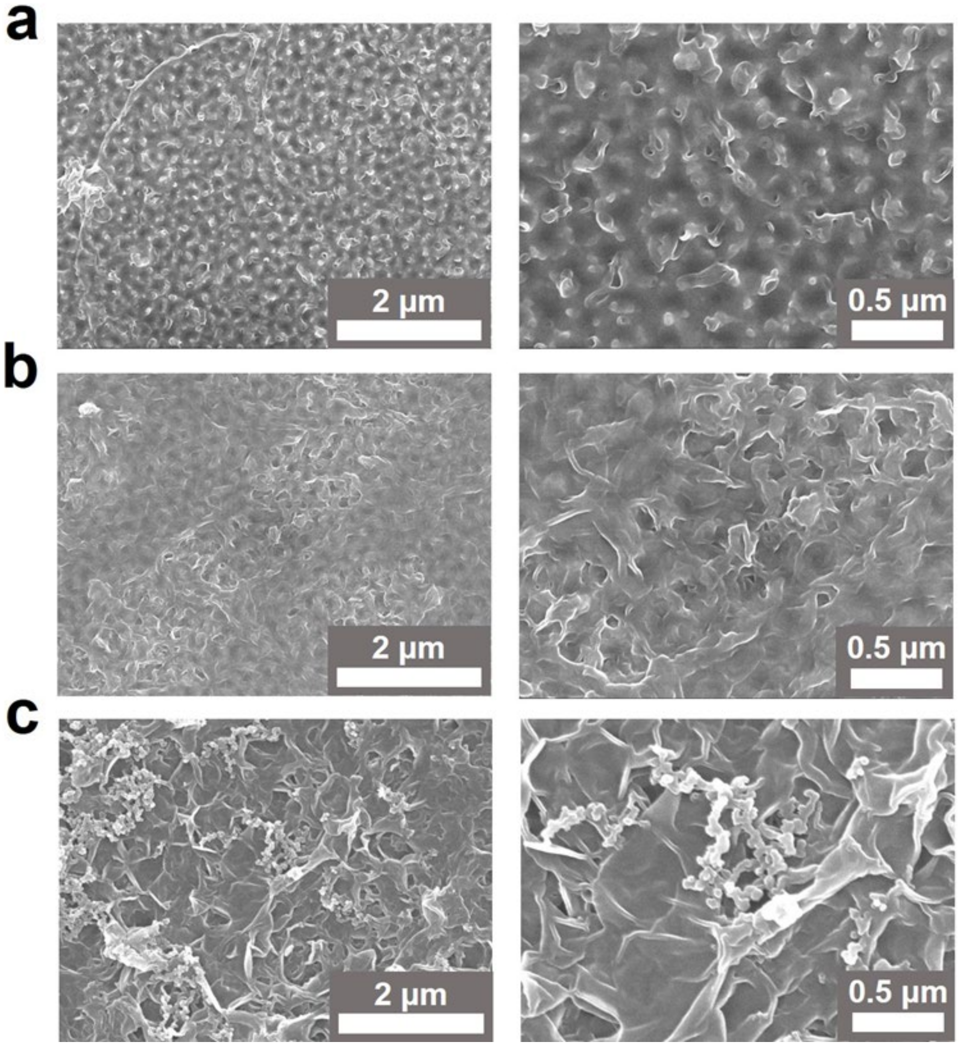

Fig. S10. FESEM images of PA nanofilms in three approaches. PA nanofilms in (a) CIP, (b) MLIP and (c) MCIP fabricated at a free-support interface and then transferred onto an AAOI (pore size 100 nm) as the support.

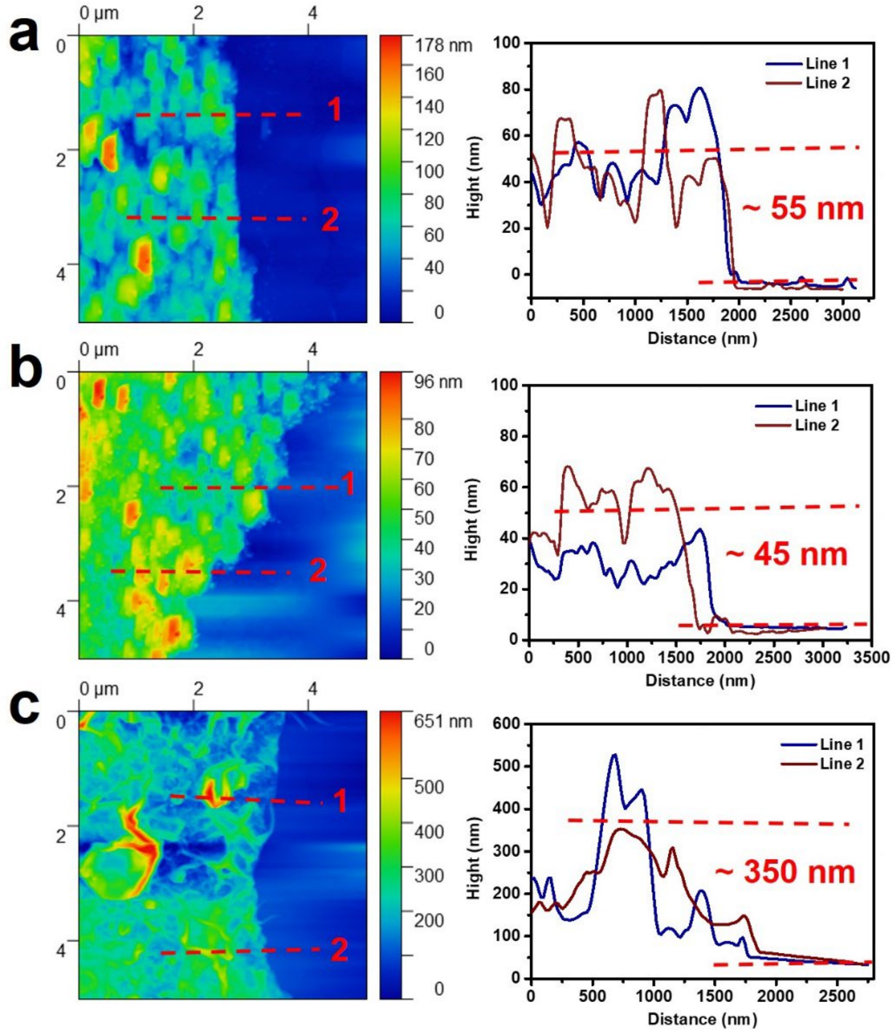

Fig. S11. Surface morphology AFM images (left-hand panel) and corresponding height profiles (right-hand panel) of PA nanofilms in three approaches. PA nanofilms in (a) CIP, (b) MLIP and (c) MCIP fabricated at a free-support interface and then transferred onto silica wafer.

Specifically, the three different free-standing PA nanofilms were transferred onto silicon wafers, so that the vertical drop between the surface of the PA nanofilm and that of the silicon wafer, characterized by AFM, can be regarded as the thickness of the free-standing PA nanofilms. The CIP-based PA nanofilms exhibited a thickness of ~55 nm, while the CIP-based PA nanofilms showed a slightly decreased thickness of ~45 nm. In contrast, the free-standing MCIP PA nanofilm revealed a remarkable increased thickness of ~350 nm. AFM characterizations exhibited similar results with those of TEM characterizations (Fig. 1 k-m), which has been discussed in the main text in detail.

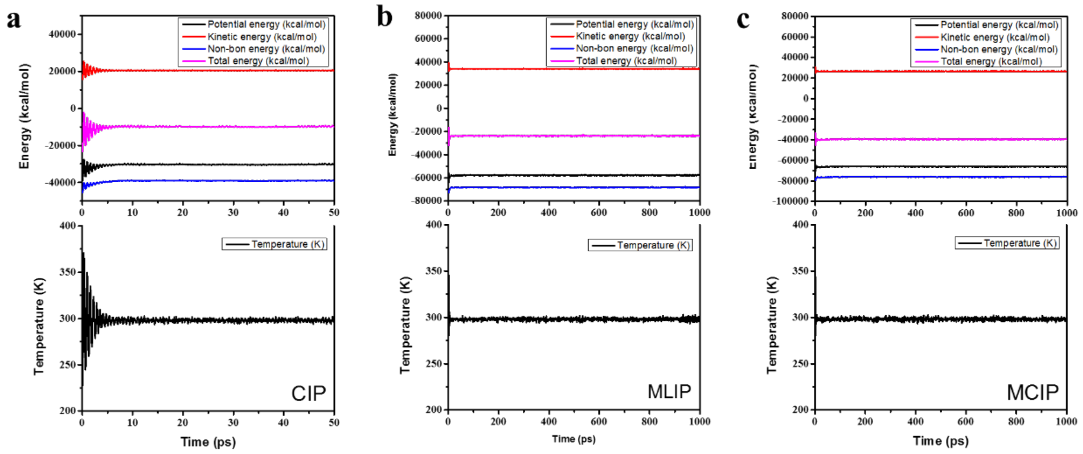

Fig. S12. Reference energy and temperature profiles in different systems. (a) CIP; (b) MLIP; (c) MCIP.

In the MD simulations, the simulation time is tested for the determination of whether the three systems reach a steady state. The energy as well as temperature evolutions were presented in Fig. S12. Specifically, the CIP system (Fig. S12a) was monitored to 50 ps for better observation of the fluctuations in energy and temperature during the initial stage. According to Fig. S12, despite the fluctuations at the very beginning, all the four reference energies (potential, kinetic, non-bond and total energy) have reached the steady values after 20 ps for the three systems. Meanwhile, the temperatures of the three systems also remained constant at around 298 K after 20 ps. Hereinafter, unless specifically stated, we used the data for the first 20 ps for analysis.

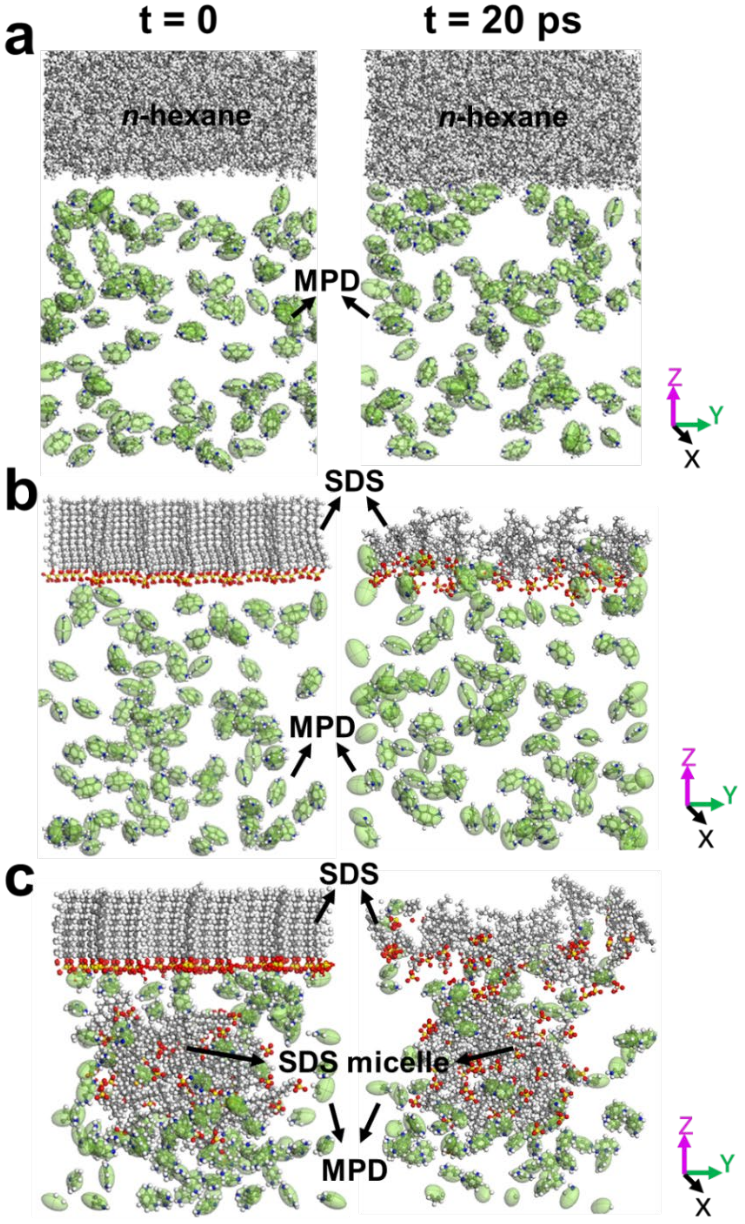

Fig. S13. Snapshots of the molecular systems (without TMC). (a) CIP system; (b) MLIP system; (c) MCIP system. Left panel: at the initial stage of the simulation (t = 0); right panel: after relaxation for 20 ps. The excessive water, *n*-hexane molecules and Na^+ ions are set as invisible for clarity. The green ellipsoids represent MPD molecules. The construction of the systems is presented in detail in **Fig. S3.**

The initial and final configurations in all MD systems were depicted in **Fig. S13**. The positions of S atoms in SDS monolayers (only in MLIP and MCIP systems) were extracted as shown in **Figs. S14** and **S15**.

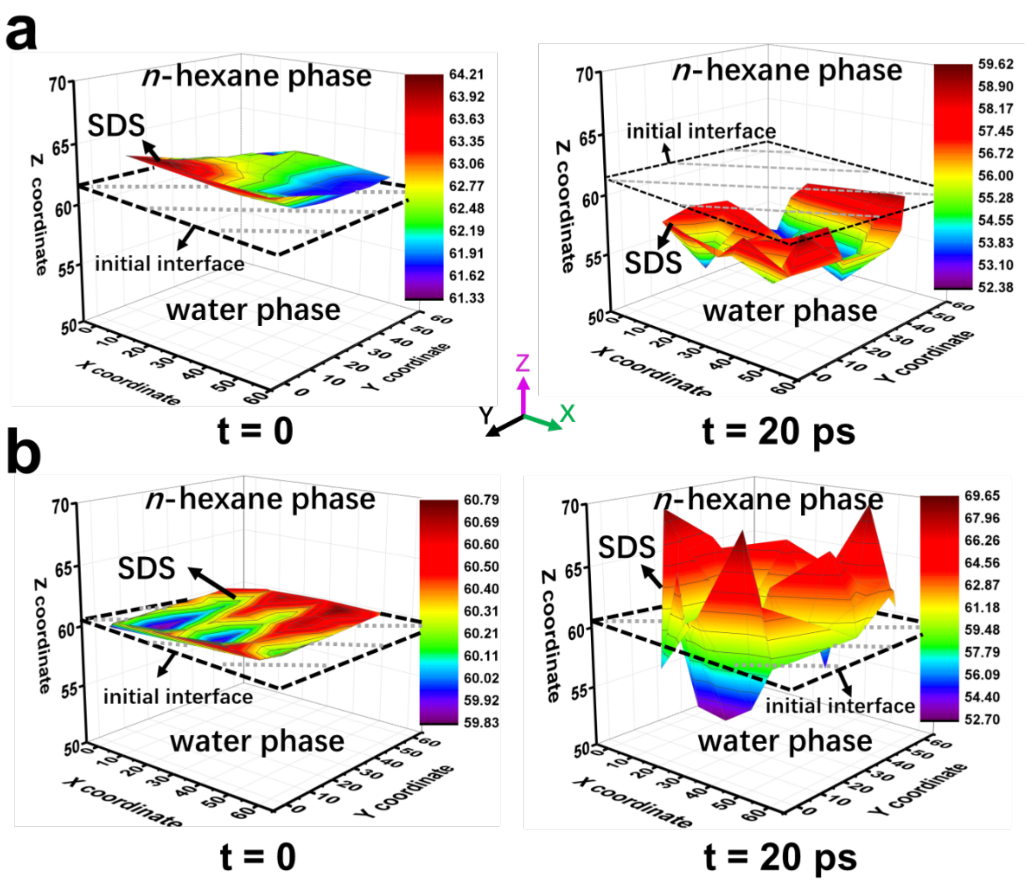

Fig. S14. Thermodynamic states of interfaces. (a) 3D map of S atoms in SDS monolayer in MLIP system (as shown in **Fig. S13b**), t = 0 (left) and t = 20 ps (right). (b) 3D map of S atoms in SDS monolayer in MCIP system (as shown in **Fig. S13c**), t = 0 (left) and t = 20 ps (right). The *Z*-coordinate indicates the energy state of the SDS molecules, as SDS with higher free energy can penetrate into the *n*-hexane phase more deeply with a higher value of *Z*-coordinate.

In a previous study, the potential of mean force with the MPD molecule at different locations along the direction perpendicular to the water/*n*-hexane interface was reported to be roughly linear near the interface (*44*), i.e., the potential of mean force with the MPD molecule from aqueous solution (~ 5 Å from the interface) into the water/*n*-hexane interface is linearly decreased from 0 to - 4 kT, and linearly increased to ~ 5 kT when it gradually penetrates into the *n*-hexane phase from

the interface until it reaches a certain distance (~ 7 Å). The relationship between the depth into the *n*-hexane phase and the potential energy of MPD could be applied to SDS head group which is also insoluble in *n*-hexane and exhibits similar polar characteristics (compared to the non-polar *n*-hexane) with MPD.

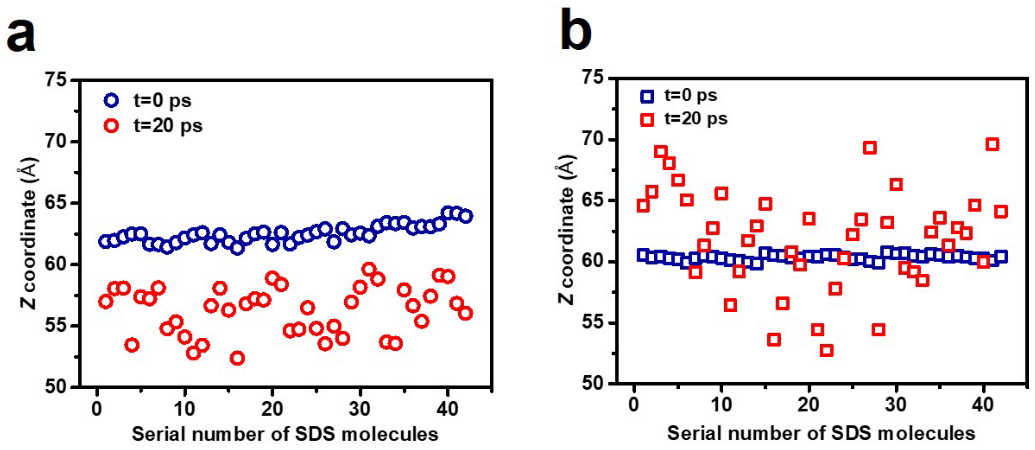

Fig. S15. Distribution of S atoms of SDS along *Z*-axis (perpendicular to the interface) in the two MD models at t = 0 and t = 20 ps. (a) MLIP system; (b) MCIP system. Each of the 42 SDS molecules in the monolayer was identified with a specific serial number from 1 to 42, according to their positions. (This figure is an analogue of the transformation of **Fig. S14** from 3D to 2D, for better visualization of their trans-interface diffusion along *Z*-axis during the simulation period.)

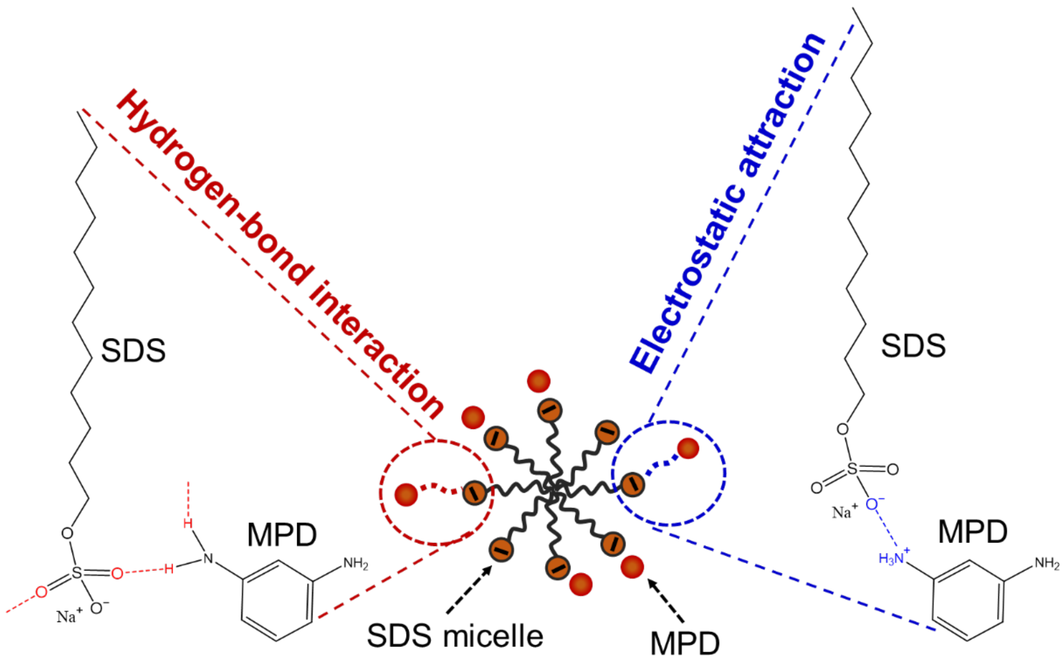

Fig. S16. The underlying molecular interactions between an SDS micelle and MPD molecules.

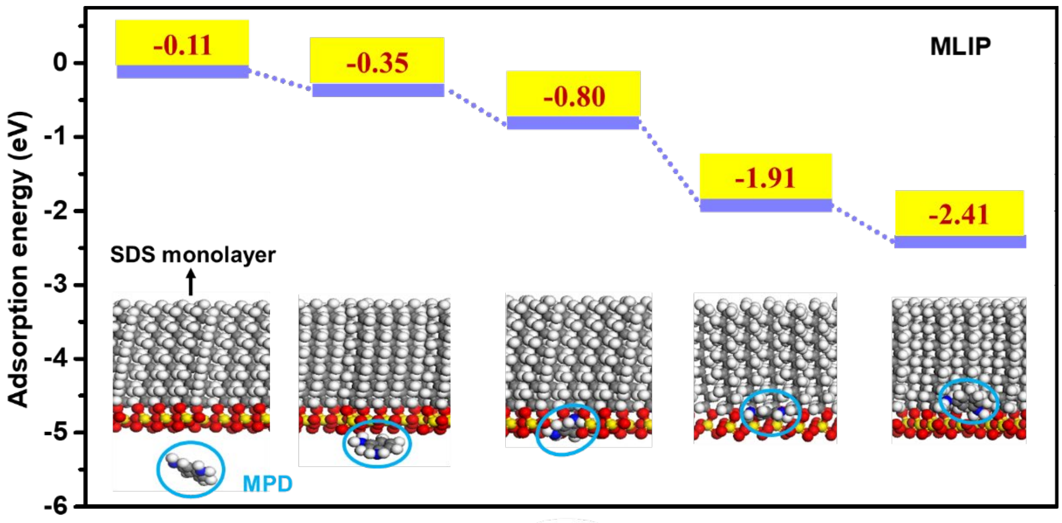

Fig. S17. Adsorption energy (values in yellow boxes) of an MPD molecule at different positions to an SDS monolayer in MLIP system. The positions of the MPD molecule are marked with blue circles. The construction of the simplified adsorption models is presented in detail in **Fig. S4.**

The adsorption energy is calculated to be negative for the MPD molecule from the near interface bulk solution to the hydrocarbon tail group of SDS monolayer, indicating the transport of a single MPD molecule from bulk solution towards the interface is an energetically favorable process at the presence of a monolayer.

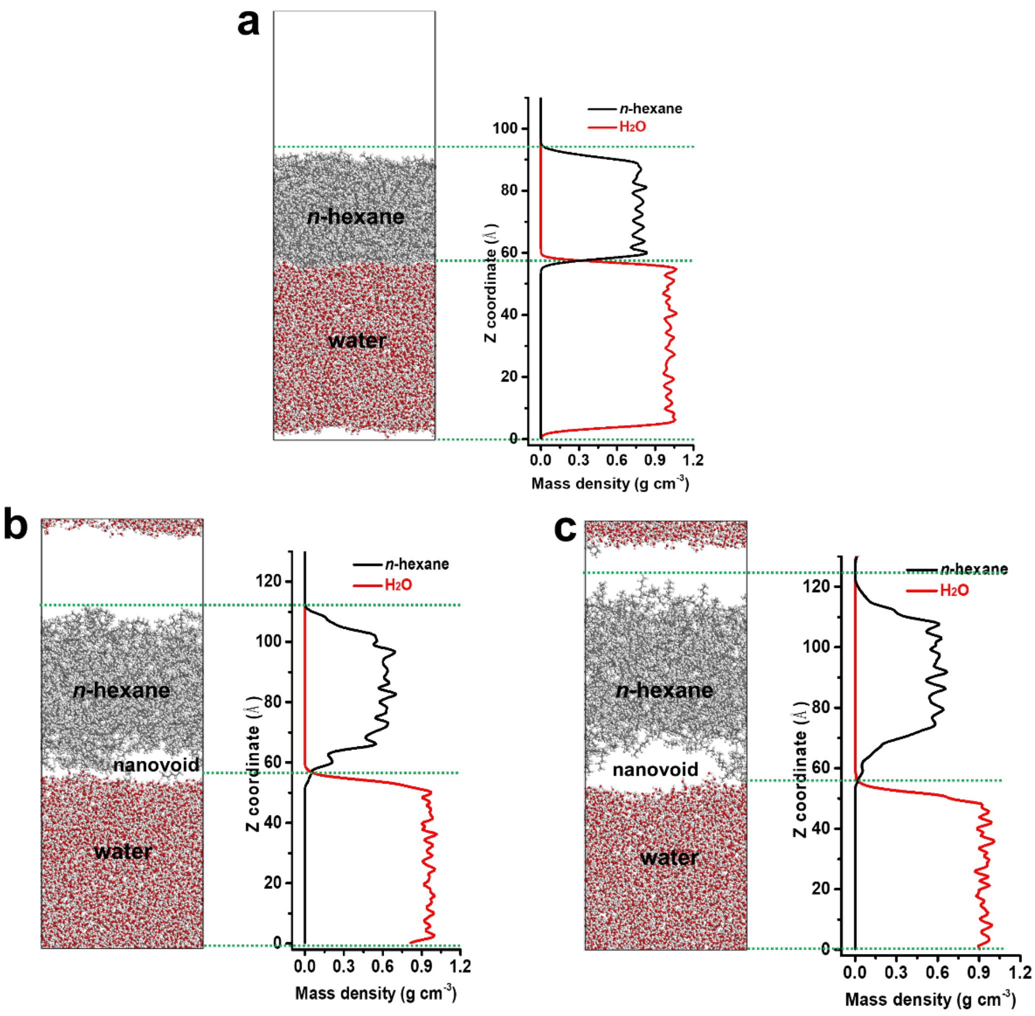

Fig. S18. Snapshots of the CIP heating systems (without MPD and TMC, left panel) and mass density of the system along *Z*-axis (right panel). (a) t = 0, at 298 K; (b) t = 20 ps, at 298 K; (c) t = 20 ps, at 318 K. The initial configuration consists of two phases, with *n*-hexane at the upper layer (containing 500 molecules of *n*-hexane, with an initial density of 0.66 g cm^{-3}) of the simulation box (dimensions 56 × 56× 150 $Å^3$), and water phase at the bottom (containing 5000 molecules of water, with an initial density of 1.0 g cm^{-3}). The interface is perpendicular to the *Z*-axis. Green dotted lines represent interfaces between different phases. The vacuum above the *n*-hexane phase was introduced to avoid possible interaction between the *n*-hexane molecules and the periodic image of the bottom layer of water molecules in the surface. White, grey and red balls represent H, C and O atoms, respectively.

It can be observed that at equilibrium state, the region between *n*-hexane surface and first layer of water molecules has a small density of sites (vacuum gap between them as shown in **Fig. S18b**)

and does not appear to be molecular sharp, because the low-density region is sufficiently large, ~ 10 Å, for the system to accommodate voids. The voids between water and *n*-hexane phases were also found in the literature (*71*). Heating is likely to accelerate the separation of the two immiscible liquids, since the two bare interfaces are repelled further from each other and a larger separation voids is generated between them (**Fig. S18c**). The water surface area and the *n*-hexane surface area are both larger than an equivalent planar system, due to surface roughness brought about by thermal fluctuations. Moreover, the voids between the two immiscible liquids can also be found at the existence of MPD molecules after equilibration (**Fig. S3a**), emphasizing the strong repulsion (interfacial tension) between water and *n*-hexane phases at molecular scale.

Despite large voids between water and *n*-hexane phases, there are small quantities of *n*-hexane molecules penetrated into water phase or water molecules buried in the *n*-hexane phase. The sites where close contact of *n*-hexane and water molecules occur constitute the nano-interfaces for initiating IP reaction because the aqueous reactive monomer (e.g., MPD in this work) can only react with TMC when the two monomers are in contact with each other. The distribution of voids and nano-interfaces are considered to be responsible for the ridge-and-valley surface structure of the CIP membranes, as the free energy cost of cavity formation necessary to solvate small molecules with size of the order of water (or *n*-hexane), i.e., MPD in the IP process, is therefore reduced at the interface. The interfacial width, or the region of voids and nano-interfaces, is most possible to be related with the roughness of the PA layer in CIP. The addition of a monolayer of SDS at the interface has substantially reduce the repulsion (interfacial tension) between water and *n*-hexane phases (**Fig. S19**), even when there are MPD molecules in the aqueous phase (equilibrated MLIP box in **Fig. S3b**), thus a smooth and stable PA layer is formed in MLIP.

In order to account for the partial molar volumes of mixing, NPT ensemble was also used to perform simulations at constant pressure. The volume changes for the two immiscible liquids are very small in comparison to the volume fluctuations presented in **Fig. S18**. Therefore, other influential factors are not explored further in this work.

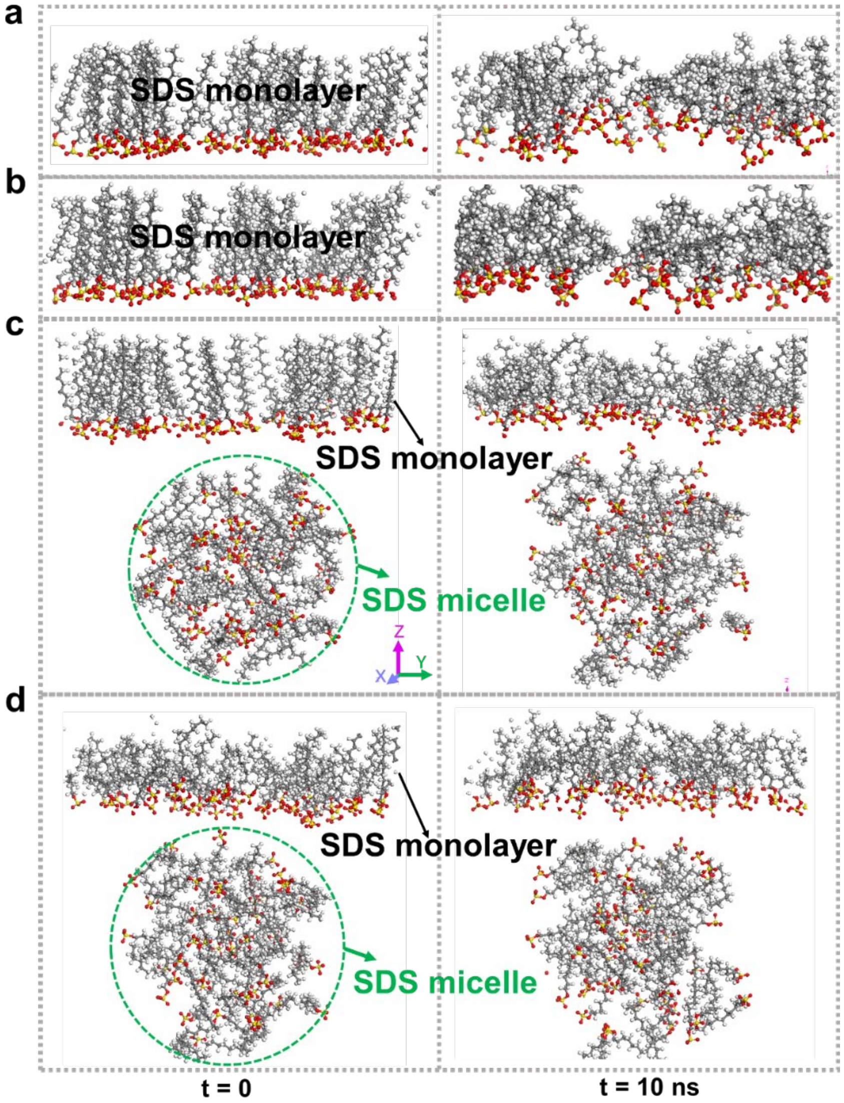

Fig. S19. Snapshots of the heating systems (without MPD and TMC). (a) MLIP system, at 298 K; (b) MLIP system, at 318 K; (c) MCIP system, at 298 K; (d) MCIP system, at 318 K. Left panel: at initial stage of the simulation (t = 0); right panel: after relaxation for 20 ps. Simulation settings are identical to those in **Figs. S3** and **S13**, except that there are no MPD molecules in the heating systems. White, grey, blue, red and yellow balls represent H, C, N, O and S atoms, respectively. The excessive water, *n*-hexane molecules and Na^+ ions are set as invisible for clarity.

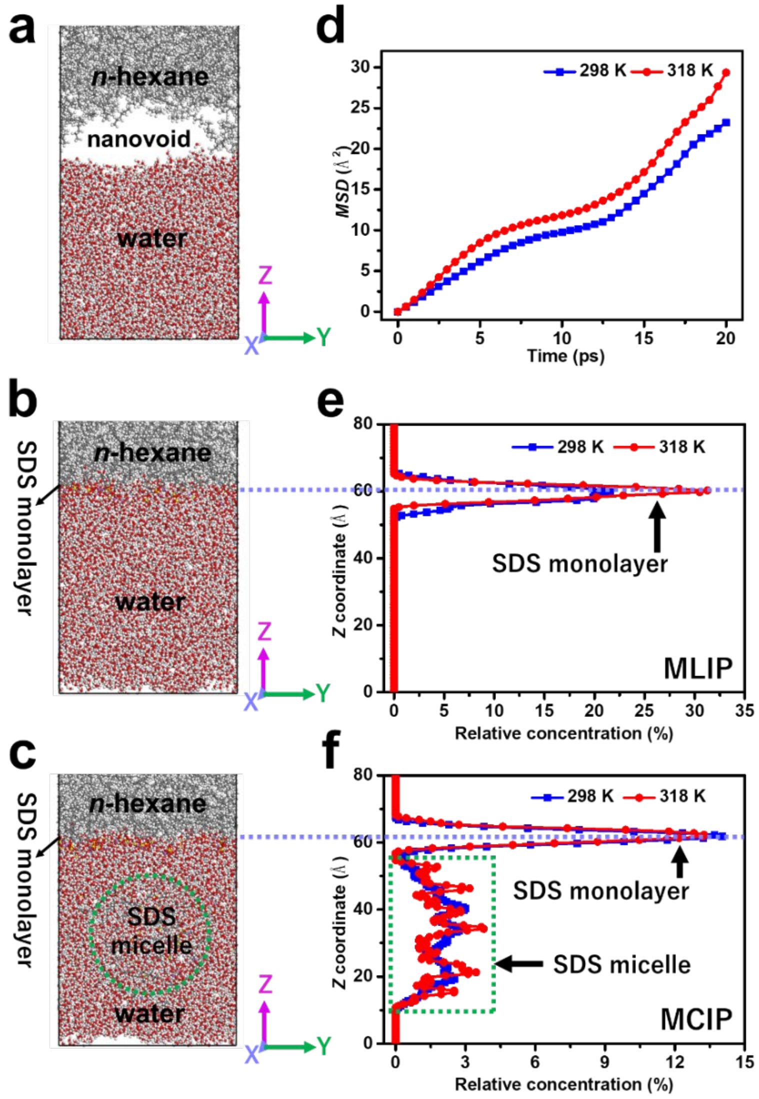

Fig. S20. Effect of heating on thermodynamic properties of the interfaces in free-standing CIP, MLIP and MCIP without reactive monomers. (a - c) snapshots of the heating systems at 318 K after equilibrium: (a) CIP system; (b) MLIP system; (c) MCIP system (green dotted circle indicates the position of a micelle). (d) *MSD* profiles of SDS molecules in MLIP system at 298 K and 318 K. (e, f) Relative concentration distributions of SDS across the interface (purple dotted line) along *Z*-axis at different temperatures in (e) MLIP system and (f) MCIP system (green dotted rectangle indicates the existence of a micelle). *Z*-axis is perpendicular to the interface.

The IP reaction is exothermic and vigorous. The heat release from the IP reaction of MPD and TMC is reported to be 7.0×10^{-7} kcal mol^{-1} ps^{-1} with an interfacial area of 16.77 nm^2, estimated from the bonding energy between MPD and TMC (*44*). To mimic the heating effect on the interface, we simplified this process by excluding MPD and TMC from the simulation systems and increased the temperature of the water/*n*-hexane/SDS system from 298 K to 318 K. Different from CIP systsem, where nanovoids and nano-interfaces of the water/*n*-hexane interface contribute to the roughness of the PA layer and are obviously responsive to heating (**Fig. 3e** and **S18**), the monolayers in both MLIP and MCIP systems are relatively stable (**Figs. S19** and **S20**). Interestingly, the diffusion of SDS is slightly enhanced by the heating, as indicated in the *MSD* curves in **Fig. 20d**, while the average concentration distribution of SDS along *Z*-axis (**Fig. 20e, f**) keeps almost unchanged for the two systems before and after heating. To verify the exact movement of SDS molecules, we checked the velocity profiles of the monolayers in MLIP and MCIP, respectively (**Fig. S21**). It is found that the heating seems to accelerate the translation and rotation of SDS molecules along the directions of *X* and *Y*-axes (parallel to the interface), especially at the water/monolayer and monolayer/*n*-hexane interfaces (**Fig. S21a, b** for MLIP; **Fig. S21d, e** for MCIP, respectively). However, the movement of SDS molecules along the *Z*-axis in an up-down motion (penetrating into *n*-hexane or dissolving in water) is less accelarated by heating (**Fig. S21c, f**). It can be explained that the heating may not be sufficient for SDS to overcome the energy penalty from water phase to *n*-hexane phase (*26*), or the heat dissipation is hindered by the monolayer (*44*). From this aspect, heating during the IP process, seems to be of insiginificant importance (or at most the secondary importance) to the thermodynamic states of the interfaces for MLIP and MCIP in this work. Therefore, the driving force of the fluctuations in the interface is primarily attributed to the electrostatic interactions among different species (i.e., MPD, monolayer and micelle) in MCIP system.

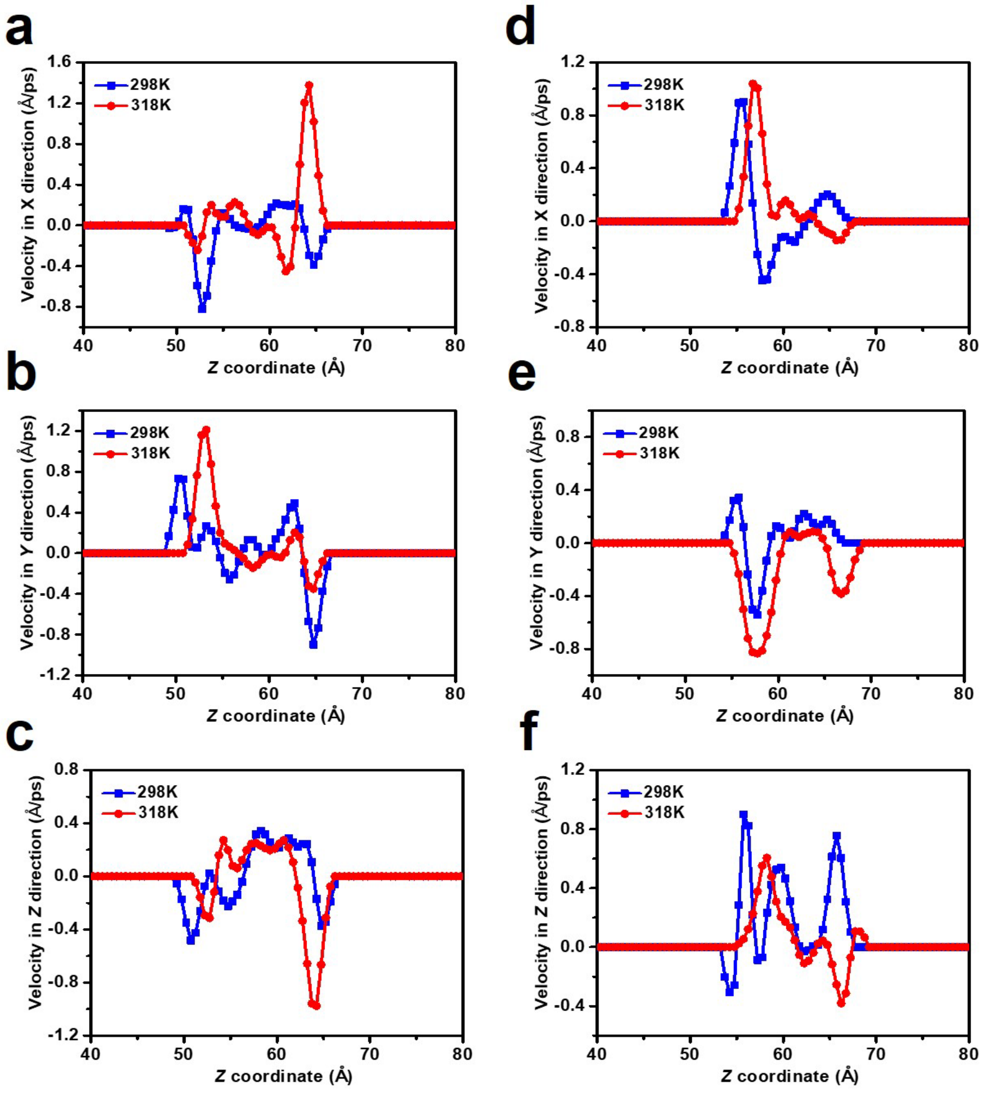

Fig. S21. Average velocity profiles in *X*, *Y*, *Z* directions of monolayer S atoms at different *Z*-coordinates in the simulation box. (a - c) Average velocity profile of monolayer S atoms in MLIP system: along the direction of (a) *X*-axis; (b) *Y*-axis; (c) *Z*-axis. (d - f) Average velocity profile of monolayer S atoms in MCIP system: along the direction of (d) *X*-axis; (e) *Y*-axis; (f) *Z*-axis. *X*, *Y*, *Z*-axises are defined similar to **Fig. S3**. The movement in *X* and *Y* directions are parellel to the interface, representing tranlation and/or rotation of the S atoms; the up-down motion in *Z* direction is perpendicular to the interface, representing the penetration into *n*-hexane phase (indicated by positive velocity in *Z* direction) and disolution in water phase (indicated by negative velocity in *Z* direction).

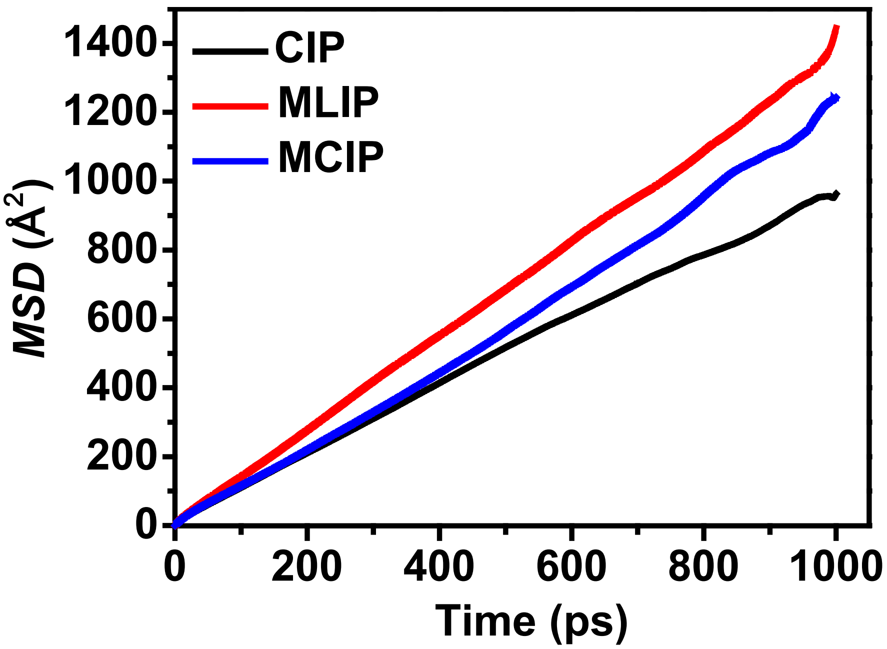

Fig. S22. ***MSD*** **profiles of MPD molecules in different systems.**

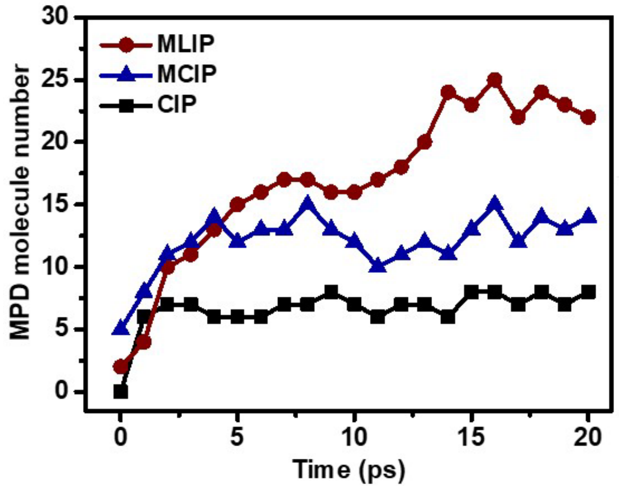

Fig. S23. Number of MPD molecules diffused into water/*n*-hexane interface along the simulation time in different systems.

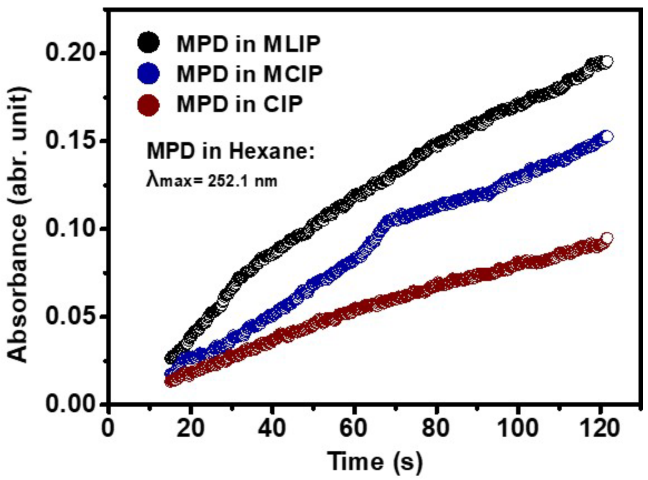

Fig. S24. Absorbance of MPD with time measured via fixed-wavelength UV-vis absorption.

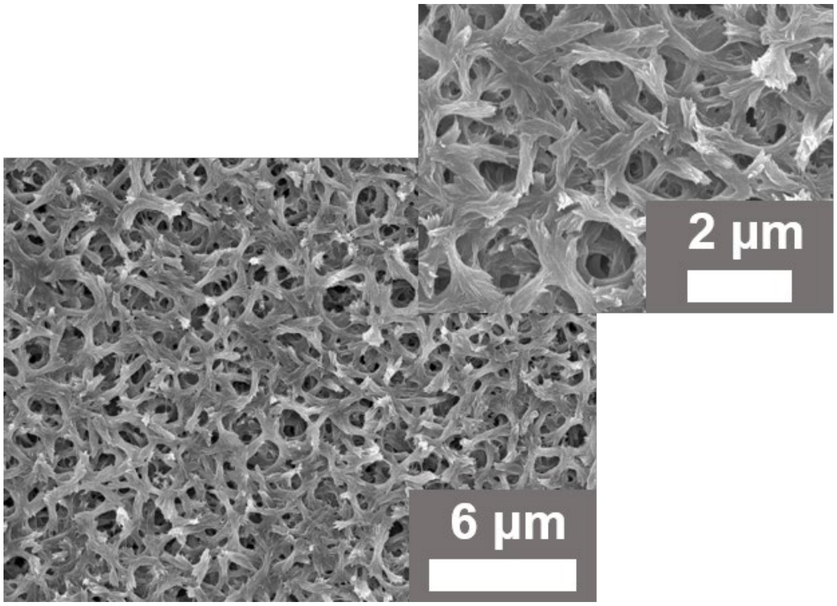

Fig. S25. Surface morphology FESEM images of the PK substrate.

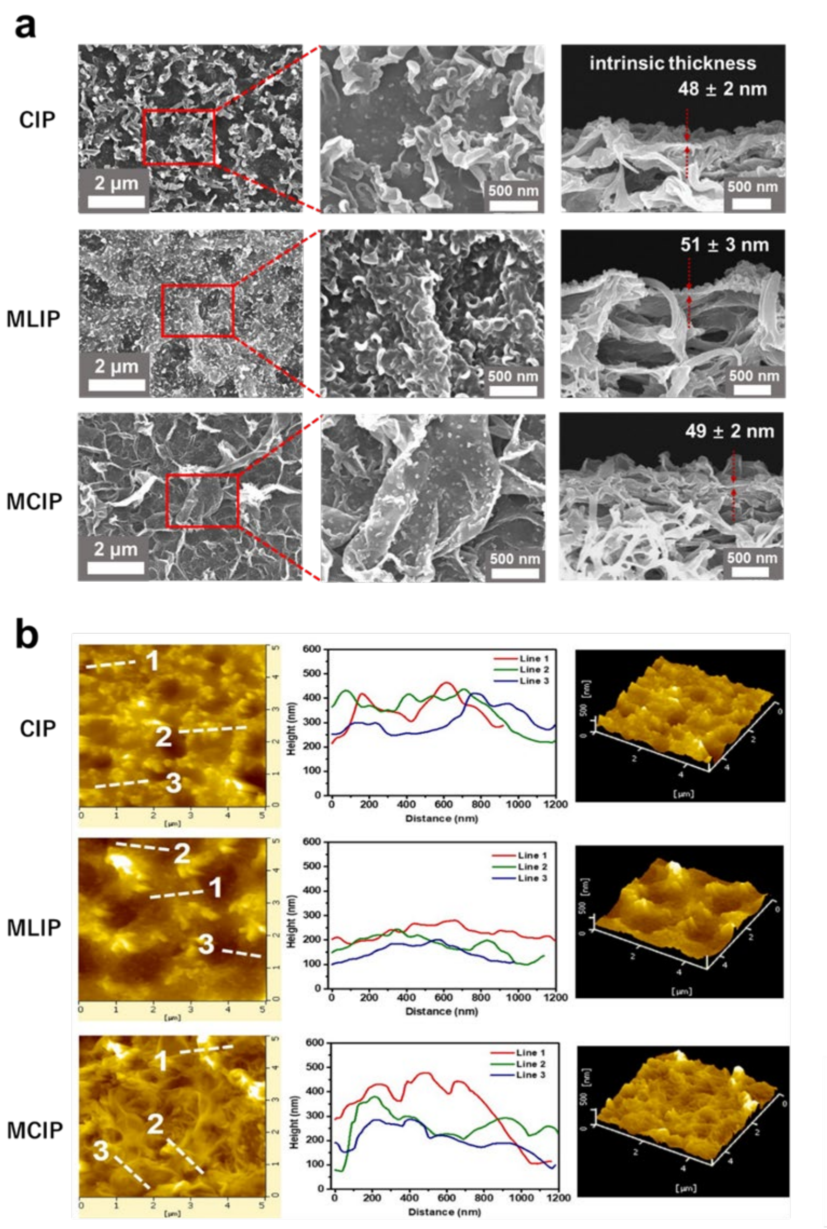

Fig. S26. Morphological properties of the PA TFC RO membranes in CIP, MLIP and MCIP. (a) FESEM images of surfaces (left panel and middle panel) and cross-sections (right panel) of the PA TFC membranes. (b) Surface roughness characterizations: 2D-AFM images (left panel), height distributions (roughness profiles, middle panel) and 3D-AFM images (right panel).

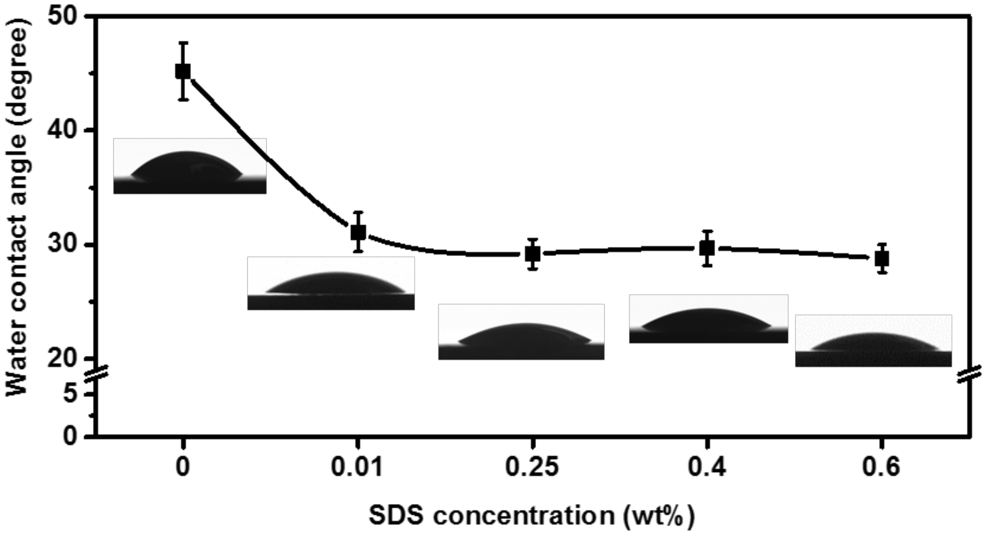

Fig. S27. Water contect angles of the PK substrates wetted by MPD aqueous solutions with different concentrations of SDS.

As shown in **Fig. S27**, MPD auqueous solution with 0.01 wt% SDS (CMC) shows a decreased static water contact angle on the PK substrate, compared with that of MPD auqueous solution without SDS, which means the SDS molecules improve the wetability of MPD auqueous solution on the substrate. However, MPD auqueous solution exhibits a nearly constant static water contact angle with a further increment in SDS concentration (above CMC). These results demonstrate that high-concentration SDS molecules can not further enhance the wetability of MPD auqueous solution on the substrate (*72, 73*).

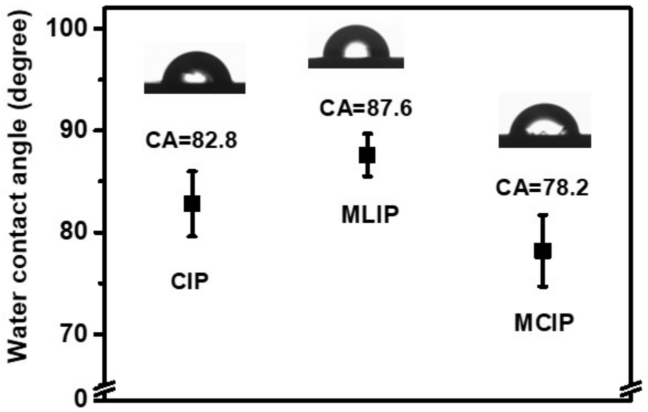

Fig. S28. Water contact angles of different PA TFC membranes.

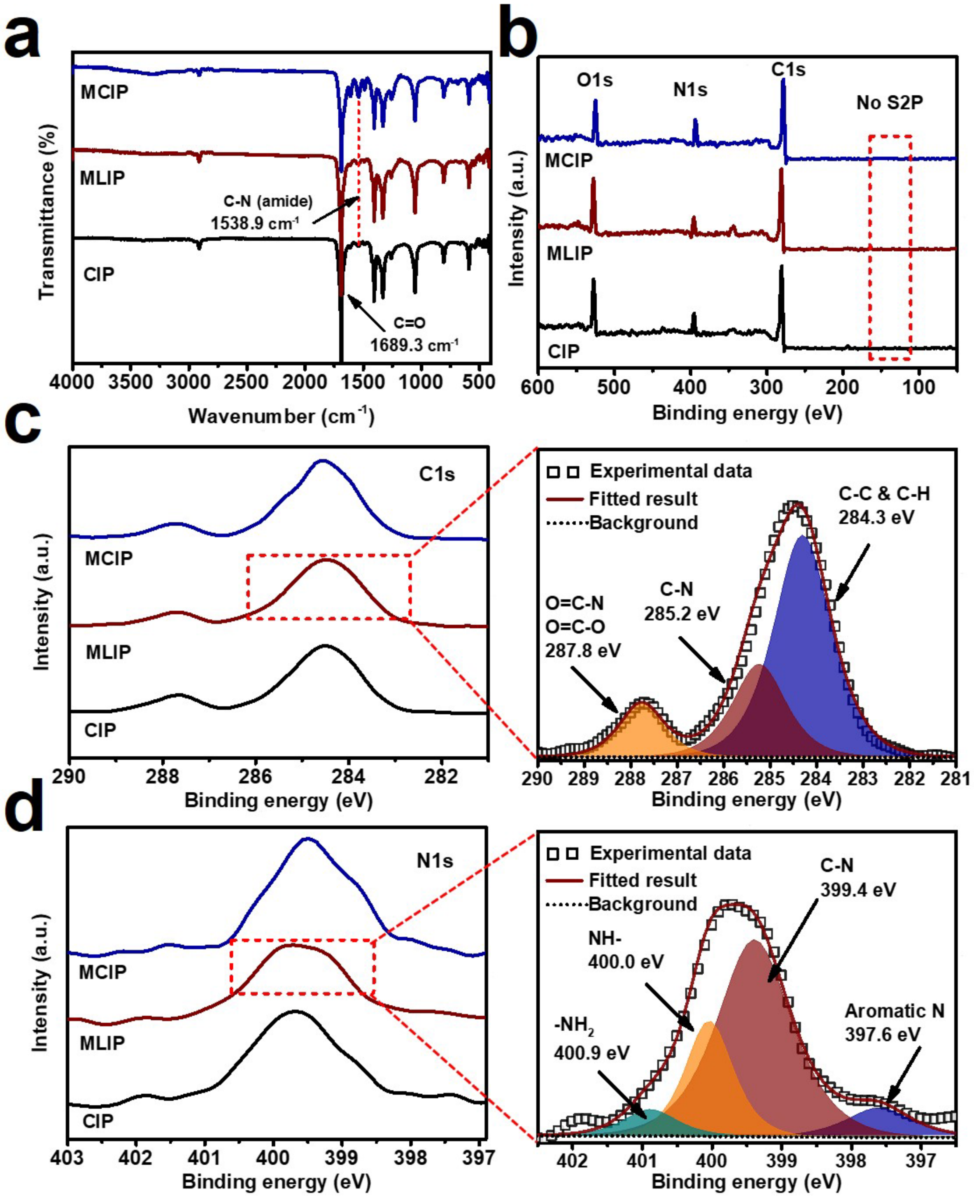

Fig. S29. FTIR and XPS characterizations of different PA TFC membranes. (a) FTIR spectra; (b) Full-scan XPS spectra; Core-level XPS spectra of C element (c) and N element (d).

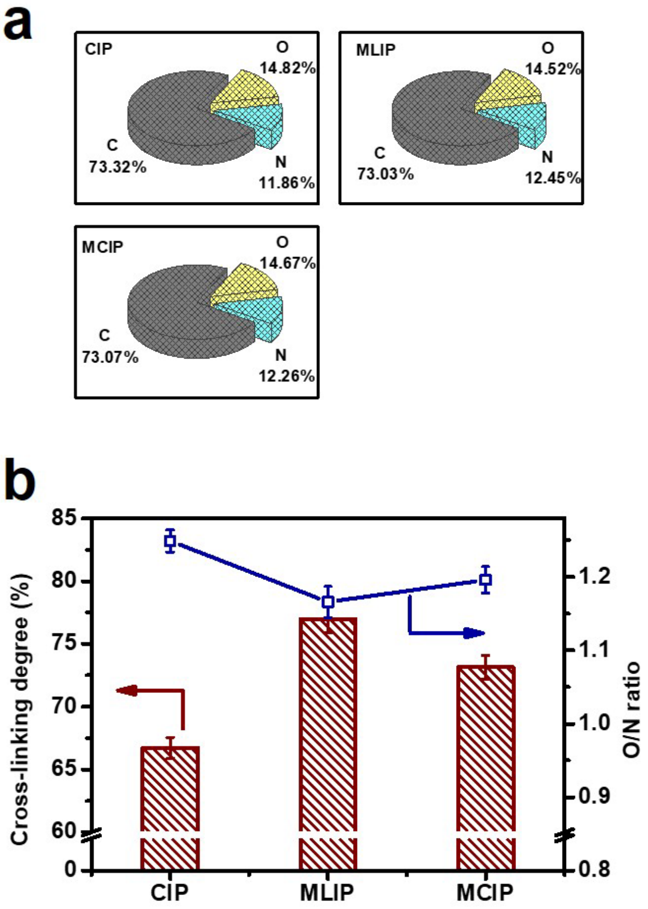

Fig. S30. Analysis of cross-linking degrees of different PA TFC membranes from XPS characterization. (a) Elemental composition; (b) O/N ratio and cross-linking degree.

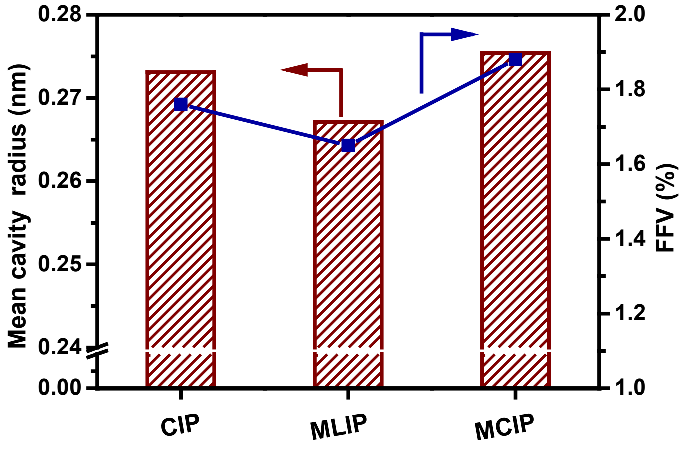

Fig. S31. Mean cavity radius and fractional free volume (FFV) of different PA TFC membranes calculated from PALS results.

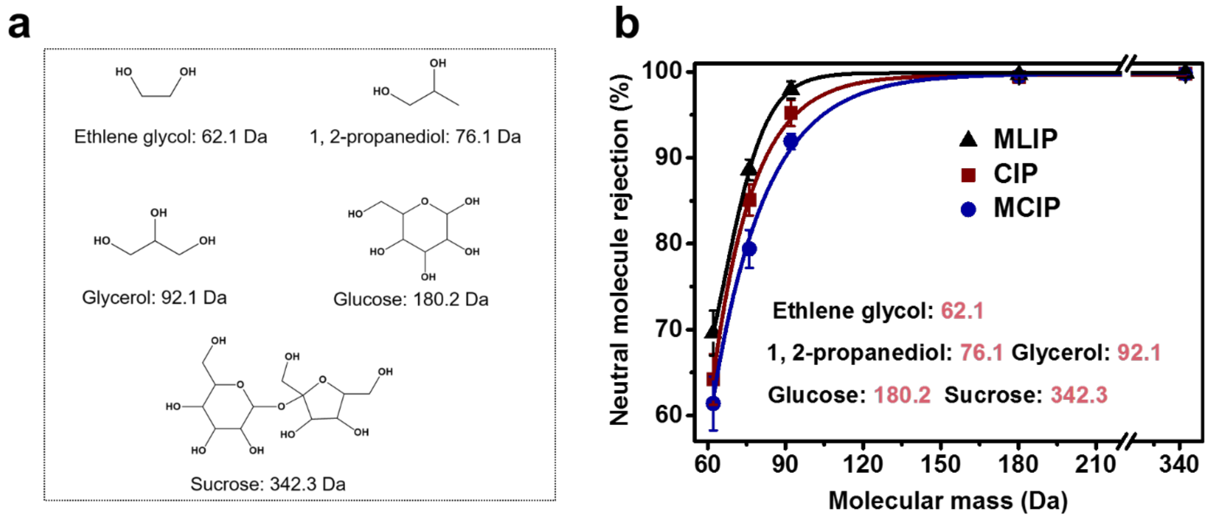

Fig. S32. Rejection to neutral molecules with different PA TFC membranes. (a) Chemical structures of the natural solutes with differnet molecular weights. (b) Rejection ratios of PA TFC membranes against neutral solutes with different molecular weights.

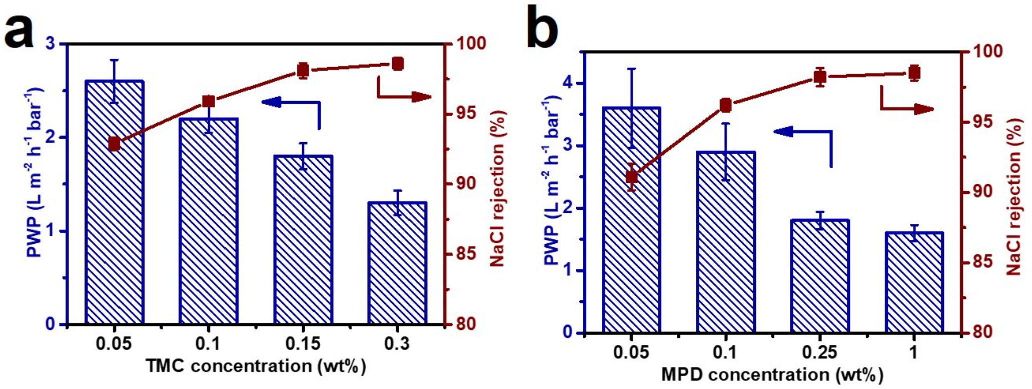

Fig. S33. Separation performance of PA TFC membranes via CIP prepared with different monomer concentrations in the reaction duration of 120 s. (a) MPD concentration fixed at 0.25 wt%; (b) TMC concentration fixed at 0.15 wt%.

The optimal condition of the IP process is selected as follows: 0.25 wt% of MPD in aqueous phase and 0.15 wt% of TMC in *n*-hexane phase.

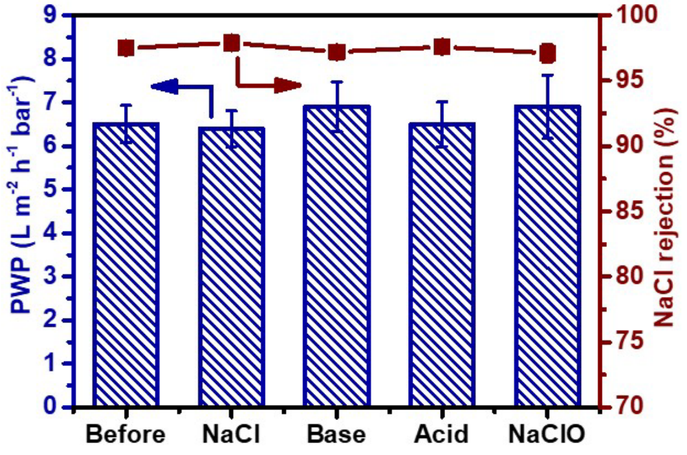

Fig. S34. Seperation performance of PA TFC membranes of the MCIP process treated with different solution samples for 8 h. Solution samples include NaCl solution (3.5 wt%), NaOH solution (pH = 12), HCl solution (pH = 1), and NaClO solution (0.1 wt%).

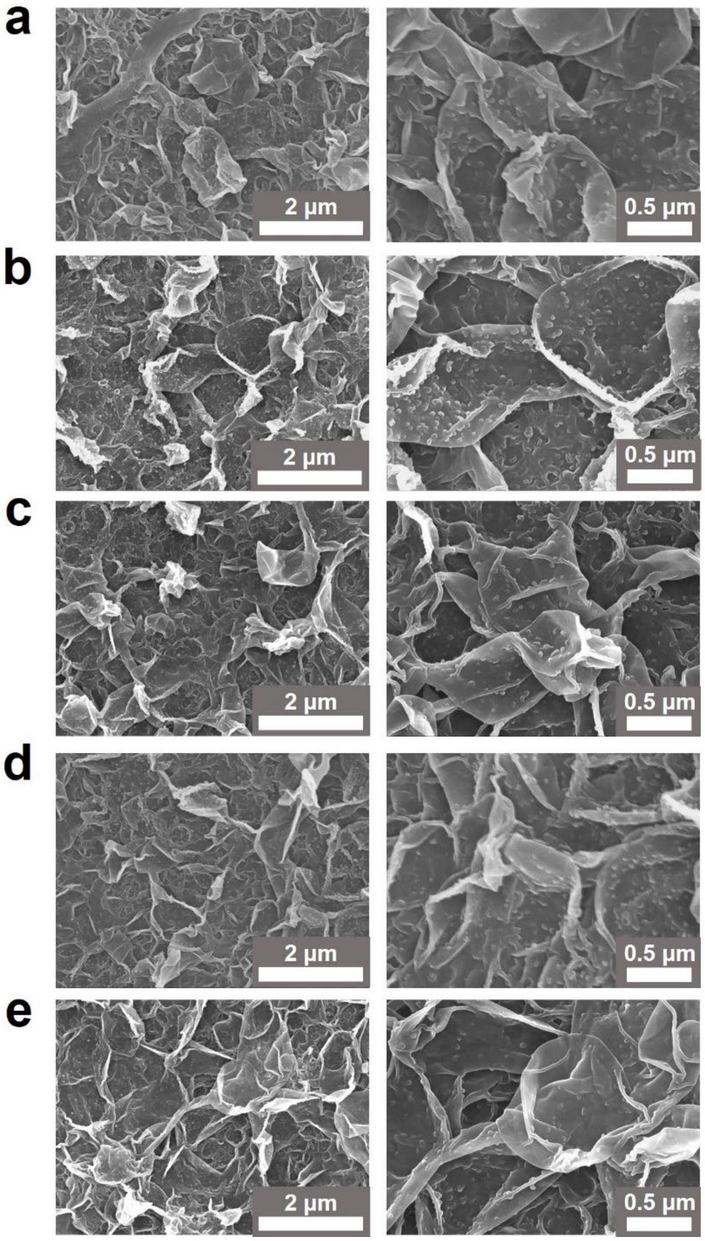

Fig. S35. Surface morphological FESEM images of PA TFC MCIP membranes treated with different solution samples for 8 h. (a) Before treatment; (b) NaCl solution (3.5 wt%); (c) NaOH solution (pH = 12); (d) HCl solution (pH = 1); (e) NaClO solution (0.1 wt%).

The performances of PA-TFC membranes from the three approaches by using 1, 6-hexanediamine as aqueous monomer are compared in Fig. S36. As a control, the PA TFC membrane formed via CIP had a water permeance of 0.9 $L \cdot m^{-2} \cdot h^{-1} \cdot bar^{-1}$, accompanied with a NaCl rejection ratio of 50.8 %. The MLIP PA TFC membrane showed an improved NaCl rejection ratio of 72.2 % and a decreased water permeance of 0.6 $L \cdot m^{-2} \cdot h^{-1} \cdot bar^{-1}$, the decrease in water permeance is attributed to the reduced effective surface area resulted from the smooth surface structures. In contrast, an enhancement in water permeance (1.2 $L \cdot m^{-2} \cdot h^{-1} \cdot bar^{-1}$) was observed for MCIP PA membranes, without sacrificing the NaCl rejection (67.6 %). The improvement in water permeance is attributed to the increased effective surface area resulted from the crumpled surface structures, as confirmed by experimental observations (Fig. 4). The change trend of the surficial morphology is consistent with our view of CIP, MLIP, and MCIP we made in the main text.

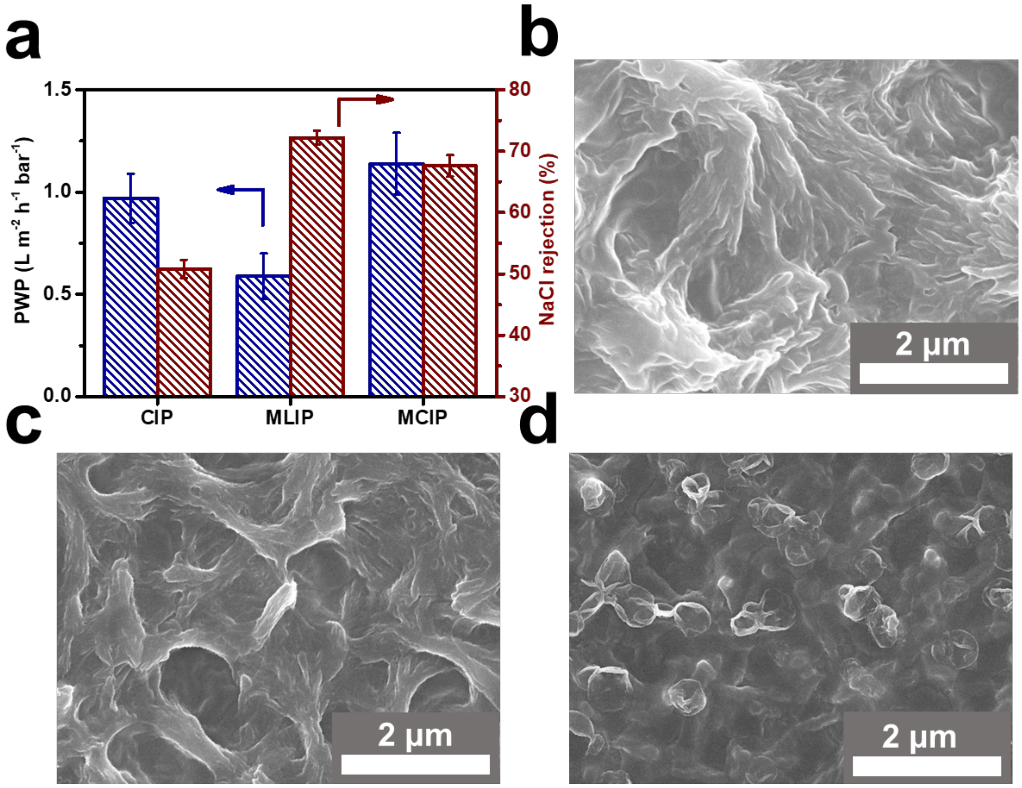

Fig. S36. Performances and morphological characterizations of different PA TFC membranes by using 0.27 wt% 1,6-hexanediamine as amine monomer. (a) Desalination performances; (b – d) Surface FESEM images: (b) CIP, (c) MLIP and (d) MCIP.

The performances of PA-TFC membranes from the three approaches by using toluene as organic phase solvent are compared in Fig. S37. The PA-TFC membranes formed via CIP and MLIP, respectively, showed similar desalination performances and surficial morphologies, which may be due to the relatively strong-polarity toluene with benzene ring structure facilitating MPD transport across the toluene-water interface, compared to the weak-polarity *n*-hexane containing aliphatic chain structure. The facilitating transport of MPD across interface induced by toluene could weaken the facilitating transport induced by SDS monolayer. In contrast, The PA-TFC membrane of MCIP exhibited an improved water permeance along with a slightly decreased NaCl rejection ratio, which is attributed to the increased effective surface area resulted from the crumpled surface structures. These crumpled surface structures can be derived from the thermodynamic fluctuations of the toluene-water interface induced by the micelles in the bulk aqueous solution, in turn shaping the initial pattern formation of the PA layer. In a word, SDS monolayer at the organic-water interface is easily influenced by organic phase, while SDS micelles in the bulk aqueous solution is more likely to maintain its own physicochemical properties facing with changes in the organic phase. As a result, the MCIP method has a wider range of applications compared to the MLIP method.

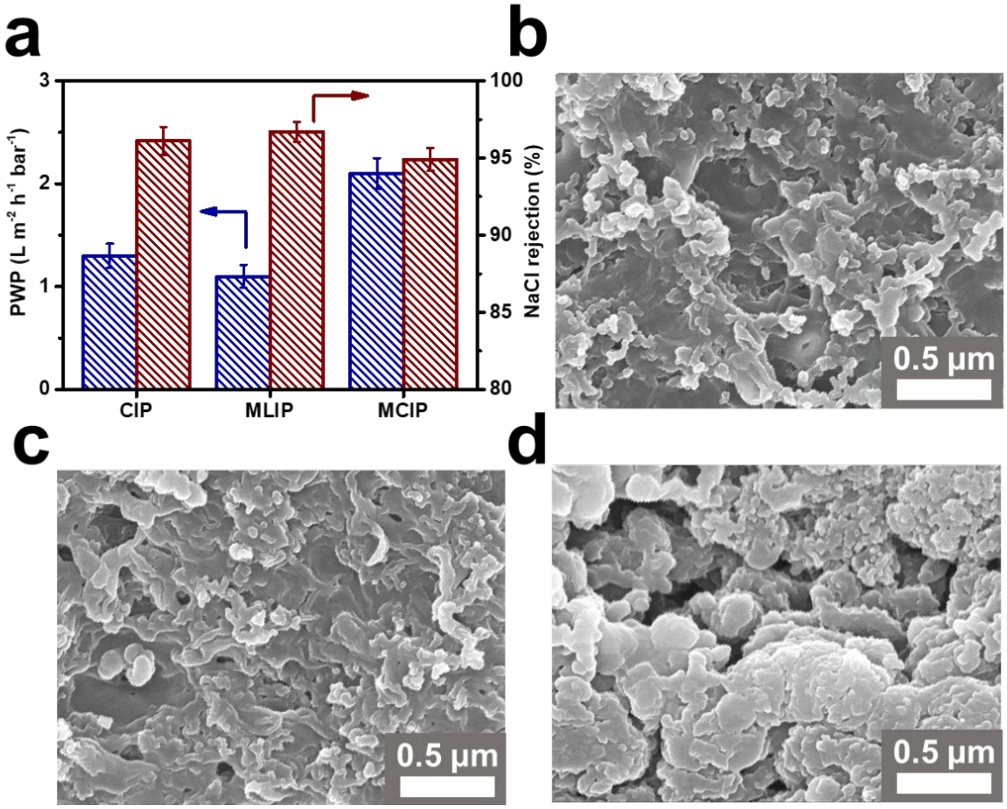

Fig. S37. Performances and morphological characterizations of different PA TFC membranes by using toluene as organic phase solvent. (a) Desalination performances; (b – d) Surface FESEM images: (b) CIP, (c) MLIP and (d) MCIP.

The performances of PA-TFC membranes from the three approaches were investigated by choosing cationic CTAB (Fig. S38 a) as surfactant. As shown in Fig. S38 b, the interfacial tension-concentration curve indicates that the CMC of CTAB in the aqueous solution is about 0.0025 wt% (interfacial tension of CMC is 1.9 mN/m, 25 °C). We used aqueous solution with 0.0025 wt% CTAB for MLIP since there was no micelle in this case, and 0.5 wt% CTAB solution (which is the same molar concentration as the SDS-based MCIP) for MCIP, respectively. As shown in Fig. S38 c, the CIP PA TFC membrane exhibited the highest water permeance, the MLIP TFC membrane showed the lowest water permeance, and the MCIP TFC membrane displayed the second highest water permeance among three kinds of nanofilms, due to the variations of surface area and density of nanofilm structure. Surprisingly, both MLIP and MCIP PA TFC membranes exhibited impaired salt rejection compared to the pristine CIP PA TFC membrane, probably caused by non-selective defects induced by CTAB. As shown in Fig. S38 d-f, the MLIP PA TFC membrane showed unconventional and smooth surficial structures which grew along the PK fibres, while the MCIP TFC membrane exhibited crumpled nodular structures, when compared to the pristine CIP PA TFC membrane. These structures of PA TFC membranes can be attributed to several reasons, as follows: when the CTAB concentration is lower than its CMC, the positive-charged CTAB is adsorbed on the surface of the negative-charged PK due to electrostatic attraction, which further led the MPD aqueous solution containing CTAB to be closely distributed on the surface structure of PK, resulting in the growth of PA along the surface of PK; when the CTAB concentration is higher than its CMC, the CTAB molecules adsorbed on PK surface repelled CTAB micelles and MPD molecules in aqueous solution, causing instability of reaction system and inducing PA to form fold morphology. In a word, CTAB monolayer and CTAB micelles can effectively regulate the IP reaction, endowing the PA nanofilms with different surficial structures and different desalination performances in the MLIP and MCIP processes, which verified that the effectiveness of MLIP and MCIP in regulating the IP reaction is not limited to the system of SDS surfactant.

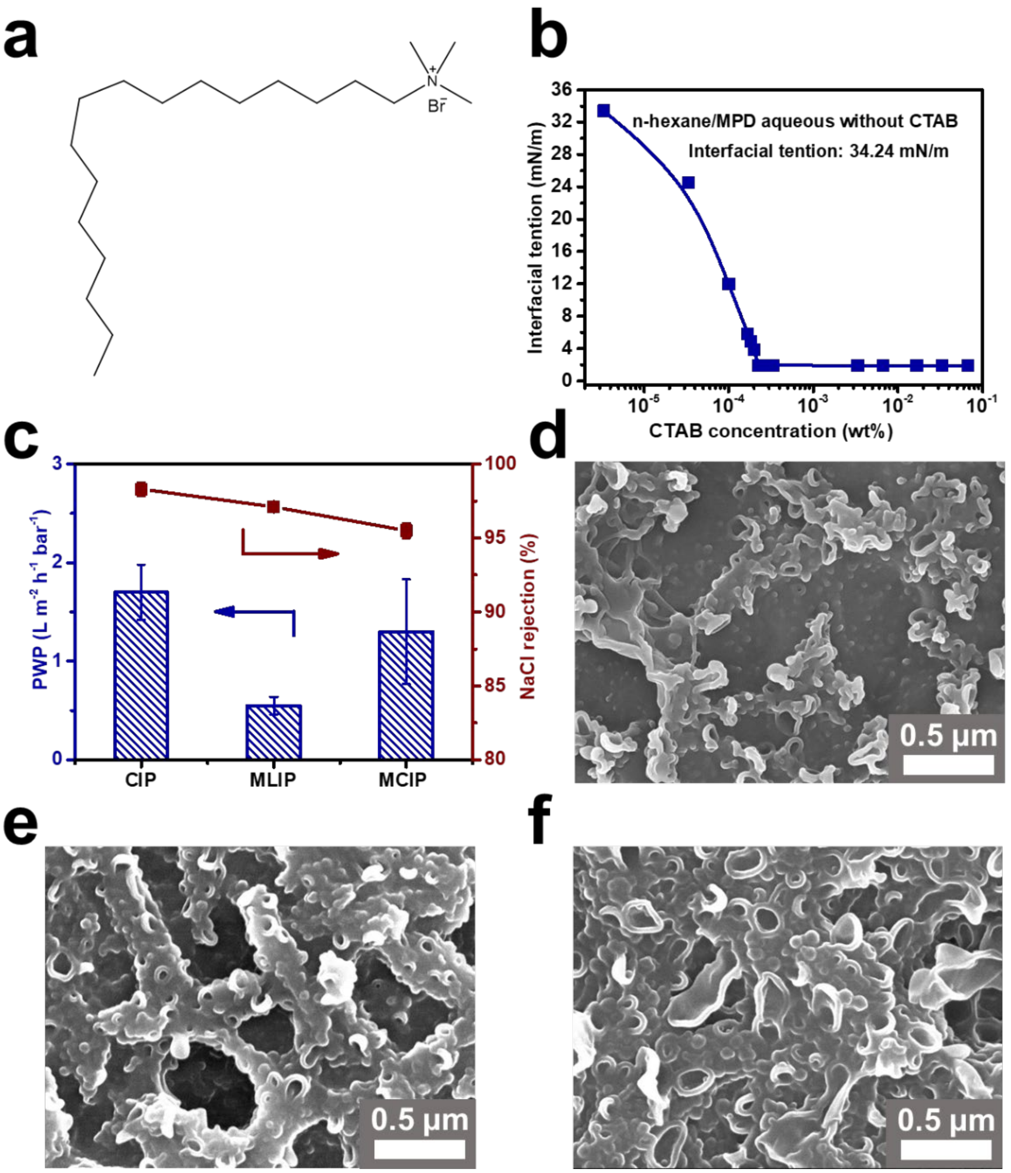

Fig. S38. **Performances and morphological characterizations of different PA TFC membranes by using CATB as surfactant.** (a) The chemical structural formula of CTAB; (b) Interfacial tension between *n*-hexane and aqueous solution (including 2 wt% TEA, 4 wt% CSA, and 0.25 wt% MPD) with different concentrations of CTAB; (c) Desalination performances; (d – f) Surface FESEM images: (d) CIP, (e) MLIP and (f) MCIP.

The performances of PA-TFC membranes from the three approaches were investigated by choosing nonionic Tween 80 (Fig. S39 a) as surfactant. As shown in Fig. S39 b, the interfacial tension-concentration curve indicates that the CMC of Tween 80 in the aqueous solution is about 0.006 wt% (interfacial tension of CMC is 13.7 mN/m, 25 °C). We used aqueous solution with 0.006 wt% Tween 80 for MLIP since there was no micelle in this case, and 1.2 wt% Tween 80 solution (which is the same molar concentration as the SDS-based MCIP) for MCIP, respectively. As shown in Fig. S39 c, the MLIP PA TFC membrane exhibited a decreased water permeance compared to the pristine CIP PA TFC membrane. In contrast, the MCIP PA TFC membrane showed an increased water permeance compared to the pristine CIP PA TFC membrane. Those variations in water permeance are mainly caused by the variations of surface area. Impressively, both MLIP and MCIP PA TFC membranes exhibited slightly decreased salt rejection compared to the pristine CIP PA TFC membrane, probably caused by non-selective defects induced by Tween 80. As shown in Fig. S39 d-f, the MLIP PA TFC membrane showed abundant small nodular structures, while the MCIP TFC membranes exhibited crumpled leaf-like structures, when compared to the pristine CIP PA TFC membrane. As shown in Fig. S39 a, there are many strongly negatively charged oxygen atoms in the structure of Tween 80, endowing Tween 80 surfactant with similar functionality to anionic SDS surfactant. As a result, Tween 80-based MLIP PA TFC membrane showed abundant small nodular structures, while the MCIP TFC membrane exhibited crumpled leaf-like structures, when compared to the pristine CIP PA TFC membrane. However, Tween 80 induced some defects in the PA nanofilm due to its large molecular structure, resulting in lower salt rejection with the PA membranes.

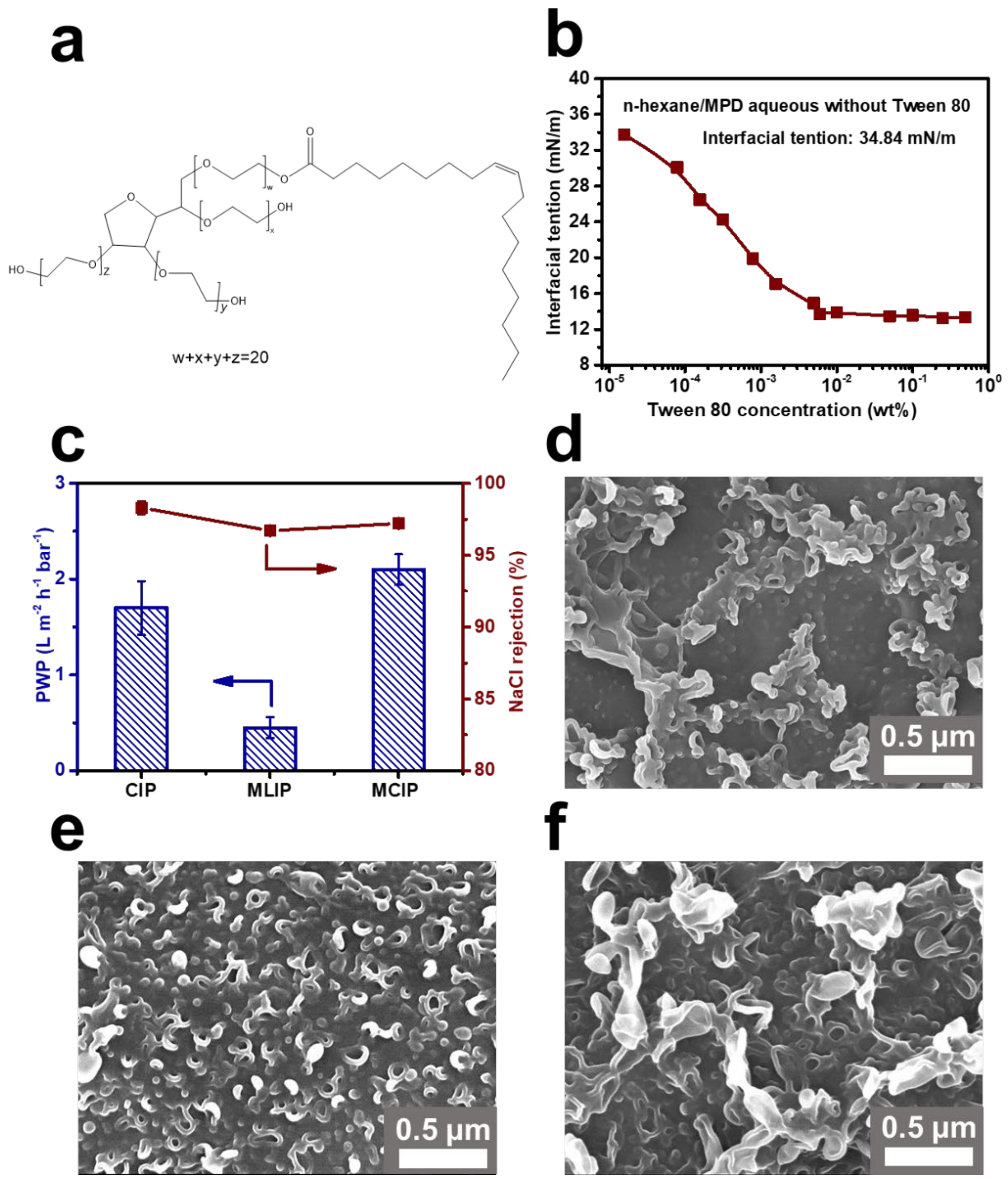

Fig. S39. Performances and morphological characterizations of different PA TFC membranes by using Tween 80 as surfactant. (a) The chemical structural formula of Tween 80; (b) Interfacial tension between *n*-hexane and aqueous solution (including 2 wt% TEA, 4 wt% CSA, and 0.25 wt% MPD) with different concentrations of Tween 80; (c) Desalination performances; (d – f) Surface FESEM images: (d) CIP, (e) MLIP and (f) MCIP.

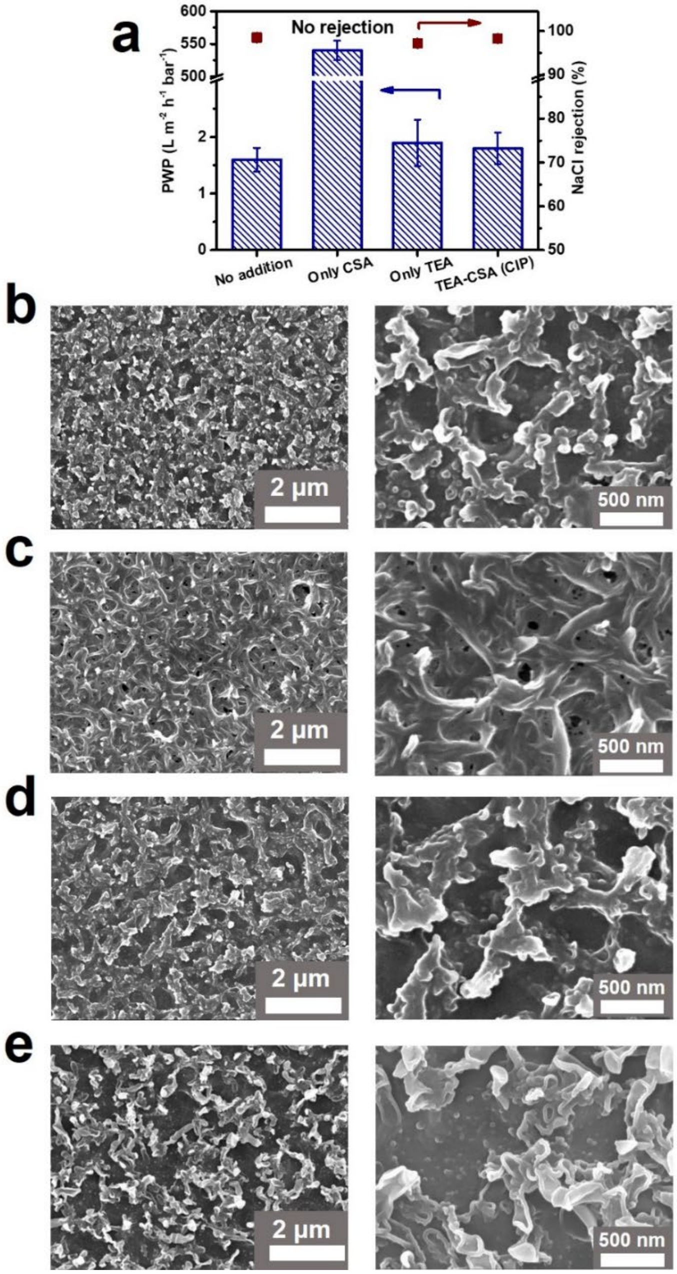

Fig. S40. Influence of chemical additives on PA TFC membranes. (a) Separation performance of different PA TFC membranes; Surface FESEM images of PA TFC membranes prepared (b) without TEA-CSA additives, (c) with 2 wt% TEA, (d) with 4 wt% CSA, and (e) with 2 wt% TEA and 4 wt% CSA.

The separation performance and FESEM images of PA membrane prepared without TEA-CSA additives, with 2 wt% TEA, with 4 wt% CSA, and with 2 wt% TEA and 4 wt% CSA were

presented in **Fig. S40**. Compared with PA membrane without any additions, PA membrane only with 4 wt% CSA showed non-selective separation performance due to many obvious surficial defects (**Figs. S40 a** and **S40 c**). CSA is a strong organic acid, and it can affect the pH in the aqueous amine solution, resulting in the protonation of amine groups in MPD, making it difficult for MPD molecules to diffuse into the organic phase, thus generate non-selective defect in the active layer (*74, 75*). PA membrane only with 2 wt% TEA exhibited slightly enhanced water permeance with negligible decreased salt rejection, and similar surficial FESEM image with those of PA membrane without any additions. These results impliedly demonstrated that excessive TEA (relative to 0.25 wt% MPD) as a catalyst may hinder the diffusion of MPD into the *n*-hexane phase, inducing some tiny defects. PA membrane with TEA-CSA organic salts displayed slightly increased water permeance without compromising salt rejection, compared to that of PA membrane without any additions, which can be attributed to the rougher surface morphology (**Fig. S40 e**). Those results demonstrated TEA and TEA-CSA organic salts, except for CSA additions, would not exert a notable effect on the separation performance and surface morphology. Therefore, TEA-CSA organic salts, which were used into the CIP, MLIP, and MCIP process, serve mainly to affect the CMC of SDS surfactant, without directly regulating the IP process.

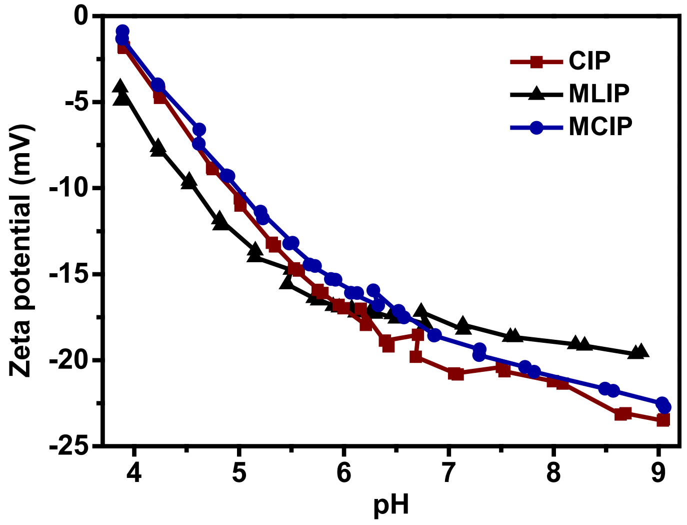

Fig. S41. Zeta potentials of different PA TFC membranes.

Table S1. Diffusion coefficients *D* (10^{-10} m^2 s^{-1}) of MPD in different systems estimated from MD simulations.

	CIP	MLIP	MCIP
D (10^{-10} m^2 s^{-1})	7.0	67.9	44.7

Table S2. PALS results of PA TFC membranes from three approaches.

Sample	τ_3 /ns	r/nm	V_3/nm^3
**CIP**	1.8650	0.2730	0.0851
**MLIP**	1.8461	0.2710	0.0833
**MCIP**	1.8810	0.2741	0.0872

τ_3: o-Ps lifetime; r: mean free-volume radius; V_3: mean free volume.

Table S3. Comparisons between this study and the reported RO membranes.

Samples	Pressure (bar)	PWP [a] LMHB[b]	NaCl rejection (%)	Year
0.10%TMC-2%MPD	15.0	0.76	93.4	2007(*76*)
0.15%TMC-3%MPD	15.0	0.69	96.0	2011(*77*)
0.1%TMC-2%MPD	16	1.18	96.0	2013(*78*)
0.15%TMC-2%MPD	15.0	1.2	96.0	2015(*79*)
0.1%TMC-2%MPD	15.0	1.3	98.1	2012(*80*)
0.1%TMC-2%MPD	20.7	1.2	90.0	2016(*81*)
0.1%TMC-3%MPD	15.5	0.87	91.0	2017(*82*)
0.1%TMC-3%MPD	15.5	0.59	99.3	2015(*83*)
2%TMC-1.2%MPD	15.0	1.12	93.5	2007(*84*)
0.1%TMC-2%MPD	20.0	0.71	99.0	2019(*85*)
0.15%TMC-2%MPD	15.5	0.97	98.8	2015(*86*)
0.15%TMC-2%MPD	15.0	1.1	98.4	2017(*87*)
0.15%TMC-2%MPD	15.0	~0.48	98.0	2017(*88*)
0.1%TMC-2%MPD	16.0	0.93	95.6	2014(*89*)
0.1%TMC-2%MPD	30.0	1.6	93.8	2014(*90*)
0.1%TMC-2%MPD	20.0	1.15	95.5	2019(*91*)
0.2%TMC-2%MPD	16.0	1.26	91.4	2013(*92*)
0.1%TMC-2%MPD	16.0	1.38	97.5	2012(*93*)
0.1%TMC-2%MPD	16.0	0.94	95.0	2014(*94*)
0.15%TMC-3%MPD	10.0	1.7	99.4	2020(*95*)
0.2%TMC-2%MPD	15.0	1.35	97.4	2017(*96*)
0.1%TMC-2%MPD	15.5	0.85	95.7	2017(*97*)
0.2%TMC-3%MPD	55.0	0.45	98.3	2017(*98*)
0.15%TMC-2%MPD	15.5	1.29	98.0	2015(*99*)
0.1%TMC-2%MPD	15.0	0.62	93.5	2019(*100*)
0.025%TMC-0.05%MPD	15.5	1.49	98.0	2018(*51*)
0.01%TMC-0.1%MPD	15.5	1.64	98.2	2017(*101*)
0.05%TMC-0.025%MPD	15.5	2.9	98.3	2020(*102*)
0.1%TMC-2%MPD	15.0	2.48	96.5	2019(*103*)
0.02%TMC-0.1%MPD	20.0	2.86	97.3	2020(*104*)
PAM (0.1 %TMC-1.5%MPD)	15.5	2.31	98.7	2021(*105*)
PAM-SAN2	15.5	4.37	98.3	2021(*105*)
0.15 %TMC-1.0%MPD	15.0	1.88	96.3	2020(*50*)
0.1 %TMC-2.0%MPD	15.5	3.23	99.5	2017(*106*)
0.1 %TMC-2.0%MPD	15.5	3.54	98.5	2017(*106*)
0.1 %TMC-2.0%MPD	15.0	2.78	99.0	2014(*107*)
0.12 %TMC-4.0%MPD	15.5	3.58	99.7	2019(*108*)
0.04 %TMC-5.6%MPD	15.5	3.22	99.2	2018(*109*)
0.1 %TMC-2.0%MPD	20.0	3.85	98.5	2020(*110*)
0.015%TMC-0.1%MPD	15.5	1.44	98.6	2022(*111*)
0.15 %TMC-2.0%MPD	15.0	1.92	99.2	2021(*112*)
0.15 %TMC-2.0%MPD	15.0	2.94	99.3	2021(*112*)
0.1 %TMC-2.0%MPD	16.0	3.80	98.6	2022(*113*)

0.2 %TMC-2.0%MPD	16.0	2.19	97.3	2021(*114*)
0.1 %TMC-2.0%MPD	55.0	0.6	98.9	2022(*115*)
0.1 %TMC-2.0%MPD	15.0	3.65	99.25	2022(*116*)
0.15 %TMC-2.0%MPD	15.0	4.83	98.3	2021(*117*)
0.1 %TMC-2.0%MPD	15.5	2.1	99.4	2021(*118*)
0.1 %TMC-2.0%MPD	15.5	3.0	99.4	2021(*118*)
0.1 %TMC-2.0%MPD	15.5	4.5	98.3	2021(*118*)
0.1 %TMC-2.0%MPD	15.5	6.3	95.2	2021(*118*)
0.15 %TMC-2.0%MPD	15.0	3.74	97.0	2020(*119*)
O_2-modified XLE RO	15.0	6.64	95.6	2021(*120*)
BW-30(commercial)	15.5	2.15	98.6	2015(*86*)
SW-30HR(commercial)	15.5	1.39	99.2	2018(*121*)
SW30XLE(commercial)	15.5	1.11	99.0	2017(*122*)
SWC4+(commercial)	15.5	1.29	99.4	2017(*97*)
DCWTT(commercial)	15.0	3.26	98.6	2011(*123*)
XLE RO (commercial)	15.0	8.31	91.4	2021(*120*)
TFN-COFs	15.0	1.68	99.2	2020(*124*)
TFN-SiO_2	15.0	3.55	99.1	2020(*124*)
TFN-Cu	27.5	2.97	98.3	2016(*125*)
TFN-CNT	15.5	3.03	97.7	2017(*126*)
TFN-GO	12.0	1.46	94.8	2018(*127*)
TFN-GO	15.5	2.58	98.6	2016(*128*)
TFN-N/S-d-GOQD	15.0	5.89	97.1	2020(*50*)
TFN-Silica-PEI	15.0	1.16	96.1	2017(*82*)
TFN-TiO_2	20.7	3.14	97.0	2018(*129*)
TFN-ZnO	15.5	2.06	98.0	2016(*130*)
TFN-Zeolite	15.5	4.78	98.8	2015(*131*)
TFN-MIL-101 (Cr)	16.0	2.20	99.0	2016(*132*)
TFN-ZIF-8	15.5	3.35	98.5	2015(*86*)
TFN-ZIF-8	15.5	3.64	98.6	2019(*133*)
TFN-PCNTs	15.5	3.31	98.5	2014(*134*)
TFN-AgNPs	20.0	2.49	95.9	2013(*135*)
TFN-CNT	15.5	2.77	99.0	2017(*136*)
TFN-CNT	15.0	3.87	96.2	2017(*137*)
TFN-CNT	15.5	2.86	95.7	2014(*138*)
TFN-TU10	20.0	3.61	98.7	2021(*139*)
TFN-ACGO	20.0	2.51	99.5	2021(*140*)
CIP	15.0	1.80	98.2	This study
MLIP	15.0	1.31	99.5	This study
MCIP	15.0	6.70	97.8	This study

Note: [a] Pure Water Permeance (PWP); [b] L m^{-2} h^{-1} bar^{-1} (LMHB).

Table S4. Conservative force parameter, a_{ij}, for interacting beads.

a_{ij}	C_3H_6	C_6H_{14}	C_6H_3	COCl	H_2N	O_4S	W
C_3H_6	25.00						
C_6H_{14}	51.28	25.00					
C_6H_3	36.11	27.07	25.00				
COCl	33.23	27.79	25.07	25.00			
H_2N	74.07	93.07	25.11	25.03	25.00		
O_4S	93.50	142.83	97.96	84.81	32.76	25.00	
W	47.44	162.30	111.79	96.55	28.08	32.66	25.00

REFERENCES AND NOTES

1. J.-M. Lehn, Toward self-organization and complex matter. *Science* **295**, 2400–2403 (2002).

2. G. M. Whitesides, B. Grzybowski, Self-assembly at all scales. *Science* **295**, 2418–2421 (2002).

3. M. Dergham, S. Lin, J. Geng, Supramolecular self-assembly in living cells. *Angew. Chem. Int. Ed.* **61**, e202114267 (2022).

4. H. Zhang, Z. Zhao, A. T. Turley, L. Wang, P. R. McGonigal, Y. Tu, Y. Li, Z. Wang, R. T. K. Kwok, J. W. Y. Lam, B. Z. Tang, Aggregate science: From structures to properties. *Adv. Mater.* **32**, e2001457 (2020).

5. Y. Tu, Z. Zhao, J. W. Y. Lam, B. Z. Tang, Aggregate science: Much to explore in the meso world. *Matter* **4**, 338–349 (2021).

6. H. Yu, X. Qiu, N. Moreno, Z. Ma, V. M. Calo, S. P. Nunes, K.-V. Peinemann, Self-assembled asymmetric block copolymer membranes: Bridging the gap from ultra- to nanofiltration. *Angew. Chem. Int. Ed. Engl.* **54**, 13937–13941 (2015).

7. B. Smit, P. A. J. Hilbers, K. Esselink, L. A. M. Rupert, N. M. van Os, A. G. Schlijper, Computer simulations of a water/oil interface in the presence of micelles. *Nature* **348**, 624–625 (1990).

8. X. Lu, M. Elimelech, Fabrication of desalination membranes by interfacial polymerization: History, current efforts, and future directions. *Chem. Soc. Rev.* **50**, 6290–6307 (2021).

9. F. Zhang, J.-B. Fan, S. Wang, Interfacial polymerization: From chemistry to functional materials. *Angew. Chem. Int. Ed. Engl.* **59**, 21840–21856 (2020).

10. M. Elimelech, W. A. Phillip, The future of seawater desalination: Energy, technology, and the environment. *Science* **333**, 712–717 (2011).

11. P. Chen, Y. Yang, B. Dong, Z. Huang, G. Zhu, Y. Cao, L.-T. Yan, Polymerization-induced interfacial self-assembly of Janus nanoparticles in block copolymers: Reaction-mediated entropy effects,

diffusion dynamics, and tailorable micromechanical behaviors. *Macromolecules* **50**, 2078–2091 (2017).

12. A. C. Balazs, T. Emrick, T. P. Russell, Nanoparticle polymer composites: Where two small worlds meet. *Science* **314**, 1107–1110 (2006).

13. D. L. Gin, R. D. Noble, Designing the next generation of chemical separation membranes. *Science* **332**, 674–676 (2011).

14. H. B. Park, J. Kamcev, L. M. Robeson, M. Elimelech, B. D. Freeman, Maximizing the right stuff: The trade-off between membrane permeability and selectivity. *Science* **356**, eaab0530 (2017).

15. J. R. Werber, C. O. Osuji, M. Elimelech, Materials for next-generation desalination and water purification membranes. *Nat. Rev. Mater.* **1**, 16018 (2016).

16. G. Belfort, Membrane filtration with liquids: A global approach with prior successes, new developments and unresolved challenges. *Angew. Chem. Int. Ed. Engl.* **58**, 1892–1902 (2019).

17. S. Karan, Z. Jiang, A. G. Livingston, Sub–10 nm polyamide nanofilms with ultrafast solvent transport for molecular separation. *Science* **348**, 1347–1351 (2015).

18. Z. Tan, S. Chen, X. Peng, L. Zhang, C. Gao, Polyamide membranes with nanoscale Turing structures for water purification. *Science* **360**, 518–521 (2018).

19. M. F. Jimenez-Solomon, Q. Song, K. E. Jelfs, M. Munoz-Ibanez, A. G. Livingston, Polymer nanofilms with enhanced microporosity by interfacial polymerization. *Nat. Mater.* **15**, 760–767 (2016).

20. B. Mi, Graphene oxide membranes for ionic and molecular sieving. *Science* **343**, 740–742 (2014).

21. L. Chen, G. Shi, J. Shen, B. Peng, B. Zhang, Y. Wang, F. Bian, J. Wang, D. Li, Z. Qian, G. Xu, G. Liu, J. Zeng, L. Zhang, Y. Yang, G. Zhou, M. Wu, W. Jin, J. Li, H. Fang, Ion sieving in graphene oxide membranes via cationic control of interlayer spacing. *Nature* **550**, 380–383 (2017).

22. C. Klaysom, S. Hermans, A. Gahlaut, S. Van Craenenbroeck, I. F. J. Vankelecom, Polyamide/polyacrylonitrile (PA/PAN) thin film composite osmosis membranes: Film optimization, characterization and performance evaluation. *J. Membr. Sci.* **445**, 25–33 (2013).

23. Y. Cui, X.-Y. Liu, T.-S. Chung, Enhanced osmotic energy generation from salinity gradients by modifying thin film composite membranes. *Chem. Eng. J.* **242**, 195–203 (2014).

24. R. Dai, H. Zhou, T. Wang, Z. Qiu, L. Long, S. Lin, C. Y. Tang, Z. Wang, Nanovehicle-assisted monomer shuttling enables highly permeable and selective nanofiltration membranes for water purification. *Nat. Water* **1**, 281–290 (2023).

25. Y. Liang, Y. Zhu, C. Liu, K.-R. Lee, W.-S. Hung, Z. Wang, Y. Li, M. Elimelech, J. Jin, S. Lin, Polyamide nanofiltration membrane with highly uniform sub-nanometre pores for sub-1 Å precision separation. *Nat. Commun.* **11**, 2015 (2020).

26. P. Sarkar, S. Modak, S. Karan, Ultraselective and highly permeable polyamide nanofilms for ionic and molecular nanofiltration. *Adv. Funct. Mater.* **31**, 2007054 (2021).

27. Q. Gan, L. E. Peng, Z. Yang, P.-F. Sun, L. Wang, H. Guo, C. Y. Tang, Demystifying the role of surfactant in tailoring polyamide morphology for enhanced reverse osmosis performance: Mechanistic insights and environmental implications. *Environ. Sci. Technol.* **57**, 1819–1827 (2023).

28. Z. Qiu, H. Han, T. Wang, R. Dai, Z. Wang, Nanofoaming by surfactant tunes morphology and performance of polyamide nanofiltration membrane. *Desalination* **552**, 116457 (2023).

29. T. E. Culp, B. Khara, K. P. Brickey, M. Geitner, T. J. Zimudzi, J. D. Wilbur, S. D. Jons, A. Roy, M. Paul, B. Ganapathysubramanian, A. L. Zydney, M. Kumar, E. D. Gomez, Nanoscale control of internal inhomogeneity enhances water transport in desalination membranes. *Science* **371**, 72–75 (2021).

30. M. Kotelyanskii, N. J. Wagner, M. E. Paulaitis, Atomistic simulation of water and salt transport in the reverse osmosis membrane FT-30. *J. Membr. Sci.* **139**, 1–16 (1998).

31. E. Harder, D. E. Walters, Y. D. Bodnar, R. S. Faibish, B. Roux, Molecular dynamics study of a polymeric reverse osmosis membrane. *J. Phys. Chem. B* **113**, 10177–10182 (2009).

32. R. Oizerovich-Honig, V. Raim, S. Srebnik, Simulation of thin film membranes formed by interfacial polymerization. *Langmuir* **26**, 299–306 (2010).

33. Y. Luo, E. Harder, R. S. Faibish, B. Roux, Computer simulations of water flux and salt permeability of the reverse osmosis FT-30 aromatic polyamide membrane. *J. Membr. Sci.* **384**, 1–9 (2011).

34. M. Ding, A. Ghoufi, A. Szymczyk, Molecular simulations of polyamide reverse osmosis membranes. *Desalination* **343**, 48–53 (2014).

35. M. Ding, A. Szymczyk, F. Goujon, A. Soldera, A. Ghoufi, Structure and dynamics of water confined in a polyamide reverse-osmosis membrane: A molecular-simulation study. *J. Membr. Sci.* **458**, 236–244 (2014).

36. T. Wei, L. Zhang, H. Zhao, H. Ma, M. S. J. Sajib, H. Jiang, S. Murad, Aromatic polyamide reverse-osmosis membrane: An atomistic molecular dynamics simulation. *J. Phys. Chem. B* **120**, 10311–10318 (2016).

37. H. F. Ridgway, J. Orbell, S. Gray, Molecular simulations of polyamide membrane materials used in desalination and water reuse applications: Recent developments and future prospects. *J. Membr. Sci.* **524**, 436–448 (2017).

38. H. Zhang, M. S. Wu, K. Zhou, A. W.-K. Law, Molecular insights into the composition–structure–property relationships of polyamide thin films for reverse osmosis desalination. *Environ. Sci. Technol.* **53**, 6374–6382 (2019).

39. P. J. Hoogerbrugge, J. M. V. A. Koelman, Simulating microscopic hydrodynamic phenomena with dissipative particle dynamics. *EPL* **19**, 155 (1992).

40. R. D. Groot, P. B. Warren, Dissipative particle dynamics: Bridging the gap between atomistic and mesoscopic simulation. *J. Chem. Phys.* **107**, 4423–4435 (1997).

41. S. Yuan, G. Zhang, J. Zhu, N. Mamrol, S. Liu, Z. Mai, P. Van Puyvelde, B. Van der Bruggen, Hydrogel assisted interfacial polymerization for advanced nanofiltration membranes. *J. Mater. Chem. A* **8**, 3238–3245 (2020).

42. S. Han, Z. Mai, Z. Wang, X. Zhang, J. Zhu, J. Shen, J. Wang, Y. Wang, Y. Zhang, Covalent organic framework-mediated thin-film composite polyamide membranes toward precise ion sieving. *ACS Appl. Mater. Interfaces* **14**, 3427–3436 (2022).

43. X. You, K. Xiao, H. Wu, Y. Li, R. Li, J. Yuan, R. Zhang, Z. Zhang, X. Liang, J. Shen, Z. Jiang, Electrostatic-modulated interfacial polymerization toward ultra-permselective nanofiltration membranes. *iScience* **24**, 102369 (2021).

44. Y. Wen, R. Dai, X. Li, X. Zhang, X. Cao, Z. Wu, S. Lin, C. Y. Tang, Z. Wang, Metal-organic framework enables ultraselective polyamide membrane for desalination and water reuse. *Sci. Adv.* **8**, eabm4149 (2022).

45. Q. Shen, Y. Lin, P. Zhang, J. Segawa, Y. Jia, T. Istirokhatun, X. Cao, K. Guan, H. Matsuyama, Development of ultrathin polyamide nanofilm with enhanced inner-pore interconnectivity via graphene quantum dots-assembly intercalation for high-performance organic solvent nanofiltration. *J. Membr. Sci.* **635,** 119498 (2021).

46. K. Guan, Y. Sasaki, Y. Jia, R. R. Gonzales, P. Zhang, Y. Lin, Z. Li, H. Matsuyama, Interfacial polymerization of thin film selective membrane layers: Effect of polyketone substrates. *J. Membr. Sci.* **640**, 119801 (2021).

47. B. Khorshidi, T. Thundat, D. Pernitsky, M. Sadrzadeh, A parametric study on the synergistic impacts of chemical additives on permeation properties of thin film composite polyamide membrane. *J. Membr. Sci.* **535**, 248–257 (2017).

48. S. Shao, F. Zeng, L. Long, X. Zhu, L. E. Peng, F. Wang, Z. Yang, C. Y. Tang, Nanofiltration membranes with crumpled polyamide films: A critical review on mechanisms, performances, and environmental applications. *Environ. Sci. Technol.* **56,** 12811–12827 (2022).

49. Z. Yang, H. Guo, Z.-K. Yao, Y. Mei, C. Y. Tang, Hydrophilic silver nanoparticles induce selective nanochannels in thin film nanocomposite polyamide membranes. *Environ. Sci. Technol.* **53**, 5301–5308 (2019).

50. Q. Shen, Y. Lin, Y. Kawabata, Y. Jia, P. Zhang, N. Akther, K. Guan, T. Yoshioka, H. Shon, H. Matsuyama, Engineering heterostructured thin-film nanocomposite membrane with functionalized graphene oxide quantum dots (GOQD) for highly efficient reverse osmosis. *ACS Appl. Mater. Interfaces* **12**, 38662–38673 (2020).

51. Z. Jiang, S. Karan, A. G. Livingston, Water transport through ultrathin polyamide nanofilms used for reverse osmosis. *Adv. Mater.* **30**, e1705973 (2018).

52. S. Tacioğlu, Micellar solutions as reaction media. *Tetrahedron* **52**, 11113–11152 (1996).

53. L. Yao, Z. Qin, Q. Chen, M. Zhao, H. Zhao, W. Ahmad, L. Fan, L. Zhao, Insights into the nanofiltration separation mechanism of monosaccharides by molecular dynamics simulation. *Sep. Purif. Technol.* **205**, 48–57 (2018).

54. J. Hu, Z. Lv, Y. Xu, X. Zhang, L. Wang, Fabrication of a high-flux sulfonated polyamide nanofiltration membrane: Experimental and dissipative particle dynamics studies. *J. Membr. Sci.* **505**, 119–129 (2016).

55. M. J. Rosen, J. T. Kunjappu, *Surfactants and Interfacial Phenomena* (John Wiley & Sons, 2012).

56. T. Shintani, A. Shimazu, S. Yahagi, H. Matsuyama, Characterization of methyl-substituted polyamides used for reverse osmosis membranes by positron annihilation lifetime spectroscopy and MD simulation. *J. Appl. Polym. Sci.* **113**, 1757–1762 (2009).

57. Q. D. Y. C. Jean, T. T. Nguyen, Free-volume hole properties in thermosetting plastics probed by positron annihilation spectroscopy: Chain extension chemistry. *Macromolecules* **28**, 8840–8844 (1995).

58. M. L. Williams, R. F. Landel, J. D. Ferry, The temperature dependence of relaxation mechanisms in amorphous polymers and other glass-forming liquids. *J. Am. Chem. Soc.* **77**, 3701–3707 (1955).

59. R. Xia, X. Cao, M. Gao, P. Zhang, M. Zeng, B. Wang, L. Wei, Probing sub-nano level molecular packing and correlated positron annihilation characteristics of ionic cross-linked chitosan membranes using positron annihilation spectroscopy. *Phys. Chem. Chem. Phys.* **19**, 3616–3626 (2017).

60. Y. C. Jean, W.-S. Hung, C.-H. Lo, H. Chen, G. Liu, L. Chakka, M.-L. Cheng, D. Nanda, K.-L. Tung, S.-H. Huang, K.-R. Lee, J.-Y. Lai, Y.-M. Sun, C.-C. Hu, C.-C. Yu, Applications of positron annihilation spectroscopy to polymeric membranes. *Desalination* **234**, 89–98 (2008).

61. M. I. Baig, P. G. Ingole, J.-D. Jeon, S. U. Hong, W. K. Choi, H. K. Lee, Water vapor transport properties of interfacially polymerized thin film nanocomposite membranes modified with graphene oxide and GO-TiO_2 nanofillers. *Chem. Eng. J.* **373**, 1190–1202 (2019).

62. G. S. Lai, W. J. Lau, P. S. Goh, A. F. Ismail, Y. H. Tan, C. Y. Chong, R. Krause-Rehberg, S. Awad, Tailor-made thin film nanocomposite membrane incorporated with graphene oxide using novel interfacial polymerization technique for enhanced water separation. *Chem. Eng. J.* **344**, 524–534 (2018).

63. B. Ukrainsky, G. Z. Ramon, Temperature measurement of the reaction zone during polyamide film formation by interfacial polymerization. *J. Membr. Sci.* **566**, 329–335 (2018).

64. R. D. Groot, K. L. Rabone, Mesoscopic simulation of cell membrane damage, morphology change and rupture by nonionic surfactants. *Biophys. J.* **81**, 725–736 (2001).

65. S.-P. Sun, T.-S. Chung, K.-J. Lu, S.-Y. Chan, Enhancement of flux and solvent stability of Matrimid® thin-film composite membranes for organic solvent nanofiltration. *AIChE J.* **60**, 3623–3633 (2014).

66. A. K. Ghosh, B.-H. Jeong, X. Huang, E. M. V. Hoek, Impacts of reaction and curing conditions on polyamide composite reverse osmosis membrane properties. *J. Membr. Sci.* **311**, 34–45 (2008).

67. P. Joos, D. Vollhardt, M. Vermeulen, Interfacial tension of sodium dodecyl sulfate solutions at the hexane-water interface. *Langmuir* **6**, 524–525 (1990).

68. M. Yamanaka, M. Aratono, H. Iyota, K. Motomura, R. Matuura, Temperature effect on the adsorption of ionic surfactants at water/hexane interface from micellar solutions. *Bull. Chem. Soc. Jpn.* **55**, 2744–2748 (1982).

69. B. Naskar, A. Dey, S. P. Moulik, Counter-ion effect on micellization of ionic surfactants: A comprehensive understanding with two representatives, sodium dodecyl sulfate (SDS) and dodecyltrimethylammonium bromide (DTAB). *J. Surfactants Deterg.* **16**, 785–794 (2013).

70. I.-C. Kim, B.-R. Jeong, S.-J. Kim, K.-H. Lee, Preparation of high flux thin film composite polyamide membrane: The effect of alkyl phosphate additives during interfacial polymerization. *Desalination* **308**, 111–114 (2013).

71. I. L. Carpenter, W. J. Hehre, A molecular dynamics study of the hexane/water interface. *J. Phys. Chem.* **94**, 531–536 (1990).

72. N. Guanhua, S. Qian, X. Meng, W. Hui, X. Yuhang, C. Weimin, W. Gang, Effect of NaCl-SDS compound solution on the wettability and functional groups of coal. *Fuel* **257**, 116077 (2019).

73. K. Jarrahian, O. Seiedi, M. Sheykhan, M. V. Sefti, S. Ayatollahi, Wettability alteration of carbonate rocks by surfactants: A mechanistic study. *Colloids Surf. A Physicochem. Eng. Asp.* **410**, 1–10 (2012).

74. V. Vatanpour, M. Sheydaei, M. Esmaeili, Box-Behnken design as a systematic approach to inspect correlation between synthesis conditions and desalination performance of TFC RO membranes. *Desalination* **420**, 1–11 (2017).

75. R. R. Gonzales, T. Shintani, S. Sunami, Y. Sasaki, K. Nakagawa, T. Yoshioka, H. Matsuyama, Monoamine-modified thin film composite nanofiltration membrane for permselective separation of fermentation bioproducts. *J. Appl. Polym. Sci.* **139**, e52460 (2022).

76. B.-H. Jeong, E. M. V. Hoek, Y. Yan, A. Subramani, X. Huang, G. Hurwitz, A. K. Ghosh, A. Jawor, Interfacial polymerization of thin film nanocomposites: A new concept for reverse osmosis membranes. *J. Membr. Sci.* **294**, 1–7 (2007).

77. M. Fathizadeh, A. Aroujalian, A. Raisi, Effect of added NaX nano-zeolite into polyamide as a top thin layer of membrane on water flux and salt rejection in a reverse osmosis process. *J. Membr. Sci.* **375**, 88–95 (2011).

78. M. Bao, G. Zhu, L. Wang, M. Wang, C. Gao, Preparation of monodispersed spherical mesoporous nanosilica–polyamide thin film composite reverse osmosis membranes via interfacial polymerization. *Desalination* **309**, 261–266 (2013).

79. A. Peyki, A. Rahimpour, M. Jahanshahi, Preparation and characterization of thin film composite reverse osmosis membranes incorporated with hydrophilic SiO_2 nanoparticles. *Desalination* **368**, 152–158 (2015).

80. J. Yin, E.-S. Kim, J. Yang, B. Deng, Fabrication of a novel thin-film nanocomposite (TFN) membrane containing MCM-41 silica nanoparticles (NPs) for water purification. *J. Membr. Sci.* **423–424**, 238–246 (2012).

81. M. Zargar, Y. Hartanto, B. Jin, S. Dai, Hollow mesoporous silica nanoparticles: A peculiar structure for thin film nanocomposite membranes. *J. Membr. Sci.* **519**, 1–10 (2016).

82. M. Zargar, Y. Hartanto, B. Jin, S. Dai, Polyethylenimine modified silica nanoparticles enhance interfacial interactions and desalination performance of thin film nanocomposite membranes. *J. Membr. Sci.* **541**, 19–28 (2017).

83. H.-R. Chae, J. Lee, C.-H. Lee, I.-C. Kim, P.-K. Park, Graphene oxide-embedded thin-film composite reverse osmosis membrane with high flux, anti-biofouling, and chlorine resistance. *J. Membr. Sci.* **483**, 128–135 (2015).

84. T. Shintani, H. Matsuyama, N. Kurata, Development of a chlorine-resistant polyamide reverse osmosis membrane. *Desalination* **207**, 340–348 (2007).

85. R. Rajakumaran, V. Boddu, M. Kumar, M. S. Shalaby, H. Abdallah, R. Chetty, Effect of ZnO morphology on GO-ZnO modified polyamide reverse osmosis membranes for desalination. *Desalination* **467**, 245–256 (2019).

86. J. Duan, Y. Pan, F. Pacheco, E. Litwiller, Z. Lai, I. Pinnau, High-performance polyamide thin-film-nanocomposite reverse osmosis membranes containing hydrophobic zeolitic imidazolate framework-8. *J. Membr. Sci.* **476**, 303–310 (2015).

87. I. H. Aljundi, Desalination characteristics of TFN-RO membrane incorporated with ZIF-8 nanoparticles. *Desalination* **420**, 12–20 (2017).

88. I. W. Azelee, P. S. Goh, W. J. Lau, A. F. Ismail, M. Rezaei-DashtArzhandi, K. Wong, M. Subramaniam, K. C. Wong, M. N. Subramaniam, Enhanced desalination of polyamide thin film nanocomposite incorporated with acid treated multiwalled carbon nanotube-titania nanotube hybrid. *Desalination* **409**, 163–170 (2017).

89. G. N. B. Baroña, J. Lim, M. Choi, B. Jung, Interfacial polymerization of polyamide-aluminosilicate SWNT nanocomposite membranes for reverse osmosis. *Desalination* **325**, 138–147 (2013).

90. P. Gorgojo, M. F. Jimenez-Solomon, A. G. Livingston, Polyamide thin film composite membranes on cross-linked polyimide supports: Improvement of RO performance via activating solvent. *Desalination* **344**, 181–188 (2014).

91. F. Wang, T. Zheng, R. Xiong, P. Wang, J. Ma, Strong improvement of reverse osmosis polyamide membrane performance by addition of ZIF-8 nanoparticles: Effect of particle size and dispersion in selective layer. *Chemosphere* **233**, 524–531 (2019).

92. H. Huang, X. Qu, H. Dong, L. Zhang, H. Chen, Role of NaA zeolites in the interfacial polymerization process towards a polyamide nanocomposite reverse osmosis membrane. *RSC Adv.* **3**, 8203–8207 (2013).

93. G. N. B. Baroña, J. Lim, B. Jung, High performance thin film composite polyamide reverse osmosis membrane prepared via *m*-phenylenediamine and 2,2′-benzidinedisulfonic acid. *Desalination* **291**, 69–77 (2012).

94. H. Zhao, S. Qiu, L. Wu, L. Zhang, H. Chen, C. Gao, Improving the performance of polyamide reverse osmosis membrane by incorporation of modified multi-walled carbon nanotubes. *J. Membr. Sci.* **450**, 249–256 (2014).

95. M. G. Shin, S. J. Kwon, H. Park, Y.-I. Park, J.-H. Lee, High-performance and acid-resistant nanofiltration membranes prepared by solvent activation on polyamide reverse osmosis membranes. *J. Membr. Sci.* **595**, 117590 (2020).

96. J. Farahbakhsh, M. Delnavaz, V. Vatanpour, Investigation of raw and oxidized multiwalled carbon nanotubes in fabrication of reverse osmosis polyamide membranes for improvement in desalination and antifouling properties. *Desalination* **410**, 1–9 (2017).

97. S.-J. Park, W. Choi, S.-E. Nam, S. Hong, J. S. Lee, J.-H. Lee, Fabrication of polyamide thin film composite reverse osmosis membranes via support-free interfacial polymerization. *J. Membr. Sci.* **526**, 52–59 (2017).

98. H. Karimi, M. B. Bajestani, S. A. Mousavi, R. M. Garakani, Polyamide membrane surface and bulk modification using humid environment as a new heat curing medium. *J. Membr. Sci.* **523**, 129–137 (2017).

99. J. Duan, E. Litwiller, I. Pinnau, Preparation and water desalination properties of POSS-polyamide nanocomposite reverse osmosis membranes. *J. Membr. Sci.* **473**, 157–164 (2015).

100. M. Fathizadeh, H. N. Tien, K. Khivantsev, Z. Song, F. Zhou, M. Yu, Polyamide/nitrogen-doped graphene oxide quantum dots (N-GOQD) thin film nanocomposite reverse osmosis membranes for high flux desalination. *Desalination* **451**, 125–132 (2019).

101. X. Song, S. Qi, C. Y. Tang, C. Gao, Ultra-thin, multi-layered polyamide membranes: Synthesis and characterization. *J. Membr. Sci.* **540**, 10–18 (2017).

102. C. Jiang, L. Zhang, P. Li, H. Sun, Y. Hou, Q. J. Niu, Ultrathin film composite membranes fabricated by novel in situ free interfacial polymerization for desalination. *ACS Appl. Mater. Interfaces* **12**, 25304–25315 (2020).

103. R. Ma, Y.-L. Ji, Y.-S. Guo, Y.-F. Mi, Q.-F. An, C.-J. Gao, Fabrication of antifouling reverse osmosis membranes by incorporating zwitterionic colloids nanoparticles for brackish water desalination. *Desalination* **416**, 35–44 (2017).

104. B. Gan, S. Qi, X. Song, Z. Yang, C. Y. Tang, X. Cao, Y. Zhou, C. Gao, Ultrathin polyamide nanofilm with an asymmetrical structure: A novel strategy to boost the permeance of reverse osmosis membranes. *J. Membr. Sci.* **612**, 118402 (2020).

105. Y.-L. Ji, H.-H. Lu, B.-X. Gu, R.-F. Ye, Y. Zhou, Q.-F. An, C.-J. Gao, Tailoring the asymmetric structure of polyamide reverse osmosis membrane with self-assembled aromatic nanoparticles for high-efficient removal of organic micropollutants. *Chem. Eng. J.* **416**, 129080 (2021).

106. Y. Li, S. Li, J. Zhu, A. Volodine, B. Van der Bruggen, Controllable synthesis of a chemically stable molecular sieving nanofilm for highly efficient organic solvent nanofiltration. *Chem. Sci.* **11**, 4263–4271 (2020).

107. T. Kamada, T. Ohara, T. Shintani, T. Tsuru, Controlled surface morphology of polyamide membranes via the addition of co-solvent for improved permeate flux. *J. Membr. Sci.* **467**, 303–312 (2014).

108. Y. Zhang, Y. Wan, G. Pan, X. Wei, Y. Li, H. Shi, Y. Liu, Preparation of high performance polyamide membrane by surface modification method for desalination. *J. Membr. Sci.* **573**, 11–20 (2019).

109. M. Shi, Z. Wang, S. Zhao, J. Wang, P. Zhang, X. Cao, A novel pathway for high performance RO membrane: Preparing active layer with decreased thickness and enhanced compactness by incorporating tannic acid into the support. *J. Membr. Sci.* **555**, 157–168 (2018).

110. X. Shan, S.-L. Li, W. Fu, Y. Hu, G. Gong, Y. Hu, Preparation of high performance TFC RO membranes by surface grafting of small-molecule zwitterions. *J. Membr. Sci.* **608**, 118209 (2020).

111. Q. Shen, Y. Lin, T. Ueda, P. Zhang, Y. Jia, T. Istirokhatun, Q. Song, K. Guan, T. Yoshioka, H. Matsuyama, The underlying mechanism insights into support polydopamine decoration toward ultrathin polyamide membranes for high-performance reverse osmosis. *J. Membr. Sci.* **646**, 120269 (2022).

112. Q. Zhao, D. L. Zhao, M. H. Nai, S. B. Chen, T.-S. Chung, Nanovoid-enhanced thin-film composite reverse osmosis membranes using ZIF-67 nanoparticles as a sacrificial template. *ACS Appl. Mater. Interfaces* **13**, 33024–33033 (2021).

113. D. Chen, Q. Liang, F. Gao, T. Liu, Y. Wu, Z. Zheng, J. Kang, R. Xu, Y. Cao, M. Xiang, Design of high-performance biomimetic reverse osmosis membranes by introducing loose liposome as an artificial water channel. *Chem. Eng. J.* **431**, 133878 (2022).

114. W.-x. Li, Z. Yang, W.-l. Liu, Z.-h. Huang, H. Zhang, M.-p. Li, X.-h. Ma, C. Y. Tang, Z.-l. Xu, Polyamide reverse osmosis membranes containing 1D nanochannels for enhanced water purification. *J. Membr. Sci.* **618**, 118681 (2021).

115. J.-A.-D. Sharabati, S. Erkoc-Ilter, S. Guclu, D. Y. Koseoglu-Imer, S. Unal, Y. Z. Menceloglu, I. Ozturk, I. Koyuncu, Zwitterionic polysiloxane-polyamide hybrid active layer for high performance and chlorine resistant TFC desalination membranes. *Sep. Purif. Technol.* **282**, 119965 (2022).

116. J. Wang, S.-L. Li, Y. Guan, C. Zhu, G. Gong, Y. Hu, Novel RO membranes fabricated by grafting sulfonamide group: Improving water permeability, fouling resistance and chlorine resistant performance. *J. Membr. Sci.* **641**, 119919 (2022).

117. Y. Qin, S. Yu, Q. Zhao, G. Kang, H. Yu, Y. Jin, Y. Cao, New insights into tailoring polyamide structure for fabricating highly permeable reverse osmosis membranes. *Desalination* **499**, 114840 (2021).

118. M. G. Shin, J. Y. Seo, H. Park, Y.-I. Park, J.-H. Lee, Overcoming the permeability-selectivity trade-off of desalination membranes via controlled solvent activation. *J. Membr. Sci.* **620**, 118870 (2021).

119. Z. Zhang, Y. Qin, G. Kang, H. Yu, Y. Jin, Y. Cao, Tailoring the internal void structure of polyamide films to achieve highly permeable reverse osmosis membranes for water desalination. *J. Membr. Sci.* **595**, 117518 (2020).

120. Y. S. Khoo, W. J. Lau, Y. Y. Liang, M. Karaman, M. Gürsoy, G. S. Lai, A. F. Ismail, Rapid and eco-friendly technique for surface modification of TFC RO membrane for improved filtration performance. *J. Environ. Chem. Eng.* **9**, 105227 (2021).

121. S.-J. Park, S. J. Kwon, H.-E. Kwon, M. G. Shin, S.-H. Park, H. Park, Y.-I. Park, S.-E. Nam, J.-H. Lee, Aromatic solvent-assisted interfacial polymerization to prepare high performance thin film

composite reverse osmosis membranes based on hydrophilic supports. *Polymer* **144**, 159–167 (2018).

122. W. Choi, S. Jeon, S. J. Kwon, H. Park, Y.-I. Park, S.-E. Nam, P. S. Lee, J. S. Lee, J. Choi, S. Hong, Thin film composite reverse osmosis membranes prepared via layered interfacial polymerization. *J. Membr. Sci.* **527**, 121–128 (2017).

123. M. Liu, Z. Chen, S. Yu, D. Wu, C. Gao, Thin-film composite polyamide reverse osmosis membranes with improved acid stability and chlorine resistance by coating *N*-isopropylacrylamide-*co*-acrylamide copolymers. *Desalination* **270**, 248–257 (2011).

124. C. Li, S. Li, J. Zhang, C. Yang, B. Su, L. Han, X. Gao, Emerging sandwich-like reverse osmosis membrane with interfacial assembled covalent organic frameworks interlayer for highly-efficient desalination. *J. Membr. Sci.* **604**, 118065 (2020).

125. M. Ben-Sasson, X. Lu, S. Nejati, H. Jaramillo, M. Elimelech, In situ surface functionalization of reverse osmosis membranes with biocidal copper nanoparticles. *Desalination* **388**, 1–8 (2016).

126. T. H. Lee, M. Y. Lee, H. D. Lee, J. S. Roh, H. W. Kim, H. B. Park, Highly porous carbon nanotube/polysulfone nanocomposite supports for high-flux polyamide reverse osmosis membranes. *J. Membr. Sci.* **539**, 441–450 (2017).

127. H. Mahdavi, A. Rahimi, Zwitterion functionalized graphene oxide/polyamide thin film nanocomposite membrane: Towards improved anti-fouling performance for reverse osmosis. *Desalination* **433**, 94–107 (2018).

128. H.-R. Chae, C.-H. Lee, P.-K. Park, I.-C. Kim, J.-H. Kim, Synergetic effect of graphene oxide nanosheets embedded in the active and support layers on the performance of thin-film composite membranes. *J. Membr. Sci.* **525**, 99–106 (2017).

129. A. Al Mayyahi, TiO_2 polyamide thin film nanocomposite reverses osmosis membrane for water desalination. *Membranes* **8**, 66–76 (2018).

130. A. S. Al-Hobaib, J. El Ghoul, I. Ghiloufi, L. El Mir, Synthesis and characterization of polyamide thin-film nanocomposite membrane reached by aluminum doped ZnO nanoparticles. *Mat. Sci. Semicon. Proc.* **42**, 111–114 (2016).

131. H. Dong, L. Zhao, L. Zhang, H. Chen, C. Gao, W. S. Winston Ho, High-flux reverse osmosis membranes incorporated with NaY zeolite nanoparticles for brackish water desalination. *J. Membr. Sci.* **476**, 373–383 (2015).

132. Y. Xu, X. Gao, X. Wang, Q. Wang, Z. Ji, X. Wang, T. Wu, C. Gao, Highly and stably water permeable thin film nanocomposite membranes doped with MIL-101 (Cr) nanoparticles for reverse osmosis application. *Materials (Basel)* **9**, 870 (2016).

133. Z. Zhai, N. Zhao, W. Dong, P. Li, H. Sun, Q. J. Niu, In situ assembly of a zeolite imidazolate framework hybrid thin-film nanocomposite membrane with enhanced desalination performance induced by noria–polyethyleneimine codeposition. *ACS Appl. Mater. Interfaces* **11**, 12871–12879 (2019).

134. H. D. Lee, H. W. Kim, Y. H. Cho, H. B. Park, Experimental evidence of rapid water transport through carbon nanotubes embedded in polymeric desalination membranes. *Small* **10**, 2653–2660 (2014).

135. J. Yin, Y. Yang, Z. Hu, B. Deng, Attachment of silver nanoparticles (AgNPs) onto thin-film composite (TFC) membranes through covalent bonding to reduce membrane biofouling. *J. Membr. Sci.* **441**, 73–82 (2013).

136. A. S. Al-Hobaib, K. M. Al-Sheetan, M. R. Shaik, M. S. Al-Suhybani, Modification of thin-film polyamide membrane with multi-walled carbon nanotubes by interfacial polymerization. *Appl. Water Sci.* **7**, 4341–4350 (2017).

137. V. Vatanpour, M. Safarpour, A. Khataee, H. Zarrabi, M. E. Yekavalangi, M. Kavian, A thin film nanocomposite reverse osmosis membrane containing amine-functionalized carbon nanotubes. *Sep. Purif. Technol.* **184**, 135–143 (2017).

138. H. J. Kim, K. Choi, Y. Baek, D.-G. Kim, J. Shim, J. Yoon, J.-C. Lee, High-performance reverse osmosis CNT/polyamide nanocomposite membrane by controlled interfacial interactions. *ACS Appl. Mater. Interfaces* **6**, 2819–2829 (2014).

139. D. L. Zhao, Q. Zhao, T.-S. Chung, Fabrication of defect-free thin-film nanocomposite (TFN) membranes for reverse osmosis desalination. *Desalination* **516**, 115230 (2021).

140. M. R. Islam, P. Khurana, P. Srikrishnarka, A. Nagar, M. Jash, S. K. Jenifer, M. A. Ganayee, M. Kumar, T. Pradeep, Aminoclay-graphene oxide composite for thin-film composite reverse osmosis membranes with unprecedented water flux and fouling resistance. *Adv. Mater. Interfaces* **8**, 2100533 (2021).
